# Synthesis, Characterization, and Screening Anticancer—Antibiofilm Activities of Theophylline Derivatives Containing CF_3_/OCF_3_ Moiety

**DOI:** 10.3390/biology14091180

**Published:** 2025-09-02

**Authors:** Serpil Demir Düşünceli, Kübra Açıkalın Coşkun, Murat Kaloğlu, Elvan Üstün, Reyhan Çalışkan, Yusuf Tutar

**Affiliations:** 1 Department of Chemistry, Faculty of Science and Arts, Inönü University, 44280 Malatya, Türkiye; serpil.demir@inonu.edu.tr (S.D.D.); murat.kaloglu@inonu.edu.tr (M.K.); 2 Department of Basic Medical Sciences, Division of Biology, Faculty of Medicine, Istanbul Aydın University, 34295 Istanbul, Türkiye; kubraacikalincoskun@aydin.edu.tr; 3 Department of Chemistry, Faculty of Science and Art, Ordu University, 52200 Ordu, Türkiye; elvanustun@odu.edu.tr; 4 Department of Medical Microbiology, Faculty of Medicine, Samsun University, 55030 Samsun, Türkiye; reyhan.caliskan@samsun.edu.tr; 5 Department of Basic Medical Sciences, Division of Biochemistry, Faculty of Medicine, Recep Tayyip Erdoğan University, 53350 Rize, Türkiye

**Keywords:** substituted theophylline, molecular docking, anticancer activity, antimicrobial, antibiofilm

## Abstract

Background: Theophylline, which is biologically important and found in tea, coffee, and cocoa beans, can be synthesized chemically or by direct extraction and concentration from natural sources. Theophylline derivatives have garnered attention in recent years for their potential therapeutic effects on *Mycobacterium tuberculosis*, antihistaminic, anti-inflammatory, and anticancer. Also, trifluoromethyl (CF_3_) group has also been widely used in drug and agrochemical design. Methods: In this study, a series of new theophylline derivatives containing substituted trifluoromethyl and trifluoromethoxy groups were synthesized. The structures of these new compounds were confirmed by NMR, FT-IR, and elemental analyses. Additionally, the anticancer activities of the molecules were analyzed against VEGFR-2, CYP P450, and estrogen receptor by molecular docking method. Furthermore, in vitro biological effects of the compounds were comprehensively evaluated in cancer (A549 and HeLa) and normal (BEAS-2B) cells. Cell viability was assessed by MTT assay, and selectivity index (SI) values were calculated to determine tumor-specific toxicity. Results: N(7)-substituted theophyllines were prepared by the reaction of 1,3-dimethyl-3,7-dihydro-1*H*-purine-2,6-dione (theophylline) and trifluoromethyl substituted benzyl halide compounds. The synthesized N(7)-substituted theophyllines were obtained as white powder in high yield. The structure of synthesized compounds was confirmed by various spectroscopic techniques such as ^1^H, ^13^C, ^19^F NMR, and FT-IR spectroscopy, and elemental analysis. The highest interaction was recorded as −5.69 kcal/mol for 3-CF_3_ substituted against VEGFR-2 structure while the best binding affinity was determined for 4-OCF_3_ substituted with −6.69 kcal/mol against Human Cytochrome P450 with in silico analysis. The in vitro anticancer activities of the molecules were also evaluated against A549 and HeLa cells, and displayed considerably higher cytotoxicity with 2-CF_3_, 3-CF_3,_ and 4-CF_3_ substituted molecules in Hela and A549 cell line. To elucidate the molecular mechanism, apoptosis-related gene expression changes were analyzed by RT-qPCR in A549 and HeLa cells treated with compound 2-CF_3_. Significant upregulation of pro-apoptotic markers and downregulation of anti-apoptotic genes were observed. Consistently, ELISA-based quantification confirmed increased protein levels of Caspase-3, BAX, and Cytochrome C, and decreased BCL-2, validating the apoptotic mechanism at the protein level. Also, the antibacterial and antibiofilm activity details of the molecules were evaluated against DNA Gyrase, and SarA crystal structures by molecular docking method. The highest interaction was recorded as −5.56 kcal/mol for 2-CF_3_ substituted with H-bonds with Asn46, Val71, Asp73, and Thr165 against DNA Gyrase crystal structure while 3-CF_3_ substituted has the best binding affinity against SarA. The in vitro antimicrobial effects of the molecules were also evaluated. Conclusions: The synthesized molecules may provide insight into the development of potential therapeutic agents to the increasing antimicrobial resistance and biofilm-forming capacity of microorganisms. Additionally, compound 2-CF_3_ substituted exhibited promising and selective anticancer activity through apoptosis induction, supported by gene and protein level evidence.

## Simple Summary

New theophylline derivatives with trifluoromethyl and trifluoromethoxy groups were synthesized and characterized using NMR, FT-IR, and elemental analyses. In vitro anticancer activities showed that 2-CF_3_, 3-CF_3_, and 4-CF_3_ substituted compounds exhibited higher cytotoxicity in Hela and A549 cell lines. The anticancer activities of the molecules were also evaluated by molecular docking against VEGFR-2, CYP P450, and estrogen receptor. Further, antibacterial activities of the compounds were determined for standard bacterial strains *Staphylococcus aureus*, *Enterococcus faecalis*, *Escherichia coli*, *Pseudomonas aeruginosa*, and yeast *Candida albicans* and analyzed against DNA Gyrase and SarA crystal structures by molecular docking as well. The theophylline derivatives provide promising drug templates against cervical carcinoma and lung carcinoma as well as the development of potential therapeutic agents to the increasing antimicrobial resistance and biofilm-forming capacity of microorganisms.

## 1. Introduction

In the modern age, cancer is still one of the most pressing issues in medicine, which means new and effective treatments must be developed. Despite major progress in the development of anticancer drugs over the past fifty years, cancer remains a major cause of death around the World [1]. The rapid increase in cancer cases worldwide has made research into innovative, highly effective, low-toxicity, easily obtainable anticancer agents increasingly important [2]. Scientists have always been interested in nitrogen-containing heterocycles because of the different forms they can take and their importance in biology. For this reason, numerous studies have been conducted into the anticancer properties of nitrogen-containing heterocyclic structures [3,4,5,6,7]. Over the centuries, bacteria and fungi have developed antimicrobial resistance to drugs, resulting in infectious diseases becoming more difficult to treat. For this reason, as in the field of cancer research, scientists are working to develop new, powerful antimicrobial agents [8]. The number and types of drugs used to treat infections caused by microorganisms has increased steadily in recent years. The focus of the research is on new drug candidates that can affect a wide range of microorganisms with the smallest possible dose, while causing minimal harm to humans. Among the molecules that stand out in antimicrobial agents are heterocyclic compounds containing nitrogen atoms. Heterocyclic compounds containing nitrogen atoms are among the molecules that stand out in antimicrobial studies [9,10,11,12].

**Figure 1:**
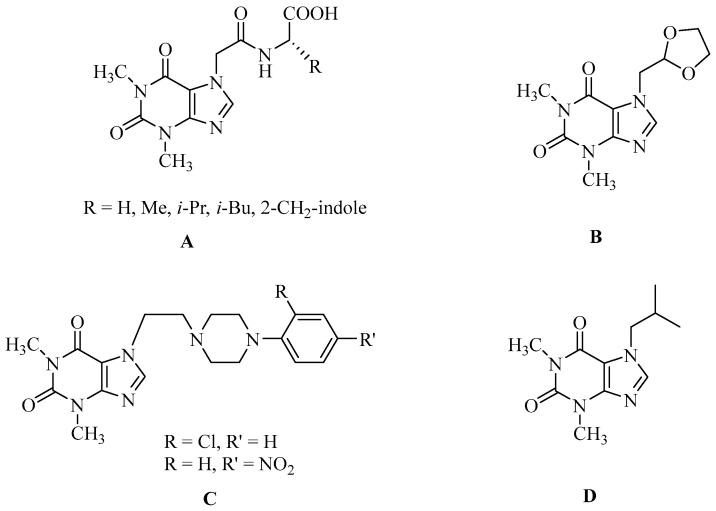
Some biologically active theophylline derivatives. (**A**) Theophylline 7-acetamides with substituents (R = H, Me, i-Pr, i-Bu, 2-CH_2_-indole); (**B**) doxofylline; (**C**) KMUP-1, KMUP-3; (**D**) isbufylline.

A significant number of bioactive compounds have purine as part of their chemical composition, making it the most widespread nitrogen-containing heterocyclic nucleus [13]. The medical field frequently uses purines and their derivatives to treat a wide range of disorders because of their numerous biological functions. Bronchiectasis antiasthma drugs include theophylline, diprophylline, proxyphylline, bamifylline, and other derivatives [14]. The pharmaceutical company classifies theophylline as a member of the xanthine group of chemical substances. These are purine derivatives with ketone groups conjugated at the purine’s carbons 2 and 6. Recent reports in the literature indicate that theophylline derivatives are a notable class of potential drug candidates. They are used to treat *Mycobacterium tuberculosis* (MTB) [15]. It was discovered by Yulian Voynikov and colleagues that amino acids derived from theophylline 7-acetamides with substituents (R = H, Me, *i*-Pr, *i*-Bu, 2-CH_2_-indole) (A) modified at the α-position are very active against MTB bacilli (H37Rv) [16]. Some N(7) substituted theophylline derivatives were found to possess an antihistaminic effect and can be used in the treatment of bronchospasm including B (doxofylline), C (KMUP-1, KMUP-3), and D (isbufylline). These compounds were found to exhibit an immunosuppressant effect that may be used for autoimmune diseases [17,18,19] (Figure 1).

The trifluoromethyl (CF_3_) group has also been widely used in drug and agrochemical design because it significantly influences their lipophilicity, chemical and metabolic stability, and binding selectivity [20]. Fluoroalkoxy compounds have attracted the interest of researchers and are increasingly used in the pharmaceutical industry, materials science, and agrochemistry [21,22]. Pretomanid [23], delamanid [24], riluzole [25], triflumuron [26], thrifluzamide [27], and flurprimidol [28] are trifluoromethoxy (OCF_3_) groups containing drugs that are now registered and used in a range of therapies. Thus, CF_3_/OCF_3_ containing substituted theophyllines have enormous potential as new biological compounds and developing a simple and practical strategy for their synthesis is of great significance.

Recently, the potential benefits of theophylline-type molecules on anticancer treatments took the attention of the researchers [29]. In early study, theophylline with 3-isobutyl-1-methylxanthine effects on MDA-MB-231 human breast cancer cells were evaluated by Slotkin and Seidler and they recorded worthwhile results for potential chemotherapeutic use [30]. Wu et al. synthesized theophylline derivative molecules with 1,2,3-triazole groups to create a possible inhibitor for IDO1 which is associated with tumor immune evasion and presented these new molecules as potential therapeutic applications [31]. Gordon et al. analyzed the anticancer activity of copper and manganese complexes with theophylline-type ligands and recorded remarkable activity against copper complexes [32].

Molecular docking method contributes to drug discovery research by identification and optimization of drug candidates and provides useful insight into the molecular interaction of certain biological processes [33]. The selection of the target molecules is one of the important parts of docking performances. VEGFR-2 plays crucial roles in the proliferation and migration of endothelial cells, therefore it is an important target molecule for the suppression of angiogenesis in diseases such as cancer [34]. Also, P450 17α-hydroxylase, which is a member of the CYP family, catalyzes androgen biosynthesis in humans and therefore, it is an important target molecule for the treatment of ovarian, colorectal, breast, and prostate cancers [35]. Additionally, since many breast cancer tumors have estrogen receptors, modulation of these receptors can be used as a treatment procedure in breast cancer cells [36]. Therefore, these three target molecules should be good for analyzing the details of the possible mechanism of the anticancer effects of drug candidates.

*Staphylococcus aureus*, *Enterococcus faecalis*, *Candida albicans*, *Pseudomonas aeruginosa*, and *Escherichia coli* possess various resistance genes and have strong biofilm-forming capacities [37,38]. Therefore, there is a clear need for the development of new therapeutic agents with both antimicrobial and antibiofilm activities. DNA gyrase affects DNA replication and maintenance of bacteria by both introducing negative supercoils into DNA and relieving positive supercoils from the replication fork [39]. Since it is an important target molecule for analyzing antibacterial activity. Moreover, SarA (Staphylococcal accessory regulator A) which is a global regulatory protein in *Staphylococcus aureus*, regulates virulence factors and promotes biofilm development. Also, it contributes to the antibiotic resistance and modulates bacterial communication by interaction with quorum-sensing system [40]. All these functions make SarA an important target for antibiotic design studies.

Based on the above, we considered it expedient to carry out the synthesis of N(7) substituted theophyllines containing trifluoromethyl and trifluoromethoxy benzyl groups. The structure of synthesized compounds was confirmed by various spectroscopic techniques such as ^1^H, ^13^C, ^19^F NMR and FT-IR spectroscopy, and elemental analysis. The molecules were evaluated in vitro anticancer activities and antimicrobial effects and additionally analyzed against VEGFR-2, CYP P450, Estrogen Receptor, DNA Gyrase, and SarA by molecular docking method.

## 2. Materials and Methods

### 2.1. Chemistry

All compounds were synthesized under open-air conditions. 1,3-dimethyl-3,7-dihydro-1*H*-purine-2,6-dione, trifluoromethyl substituted benzyl halide compounds, K_2_CO_3_, and all solvents and other reagents were commercially available and used as received without further purification. Melting point measurements of the synthesized compounds were performed in open capillary tubes with a Cole-Parmer^®^ MP-250D-F standard digital melting point instrument (Shanghai, China). Elemental analysis measurements were performed on an LECO CHNS-932 elemental analyzer (St. Joseph, MI, USA). ^1^H NMR, ^13^C NMR, and ^19^F NMR spectra were recorded at 298 K with a Bruker Avance III 400 MHz NMR spectrometer (Billerica, MA, USA) operating at 400, 101, and 376 MHz, respectively. Chemical shifts (δ) for ^1^H NMR and ^13^C NMR were given in parts per million (ppm) relative to CDCl_3_ (δ = 7.26 ppm for ^1^H NMR, δ = 77.16 ppm for ^13^C NMR). Tetramethylsilane (TMS) was used as internal standard for NMR measurements. Binding constants (*J* values) were given in Hertz. FT-IR spectra of the compounds were recorded in the range 400–4000 cm^−1^ with a Perkin Elmer Spectrum Two Spectrometer in the GladiATR unit (Attenuated Total Reflection).

General Synthesis of N(7)-Substituted Theophyllines (**1a**–**1e**)

1,3-Dimethyl-3,7-dihydro-1*H*-purine-2,6-dione and K_2_CO_3_ were added to a Schlenk tube under open-air conditions. To this mixture, 10 mL DMF was added, then trifluoromethyl substituted benzyl halide derivative (1.0 eq.) was added to the reaction mixture. The resulting white suspension was stirred at 70 °C overnight. After cooling to room temperature, 20 mL of distilled water was added. The white precipitate that formed immediately upon addition of water was filtered and washed several times with about 20 mL of water. The product was obtained as a white powder after drying. The synthesized N(7)-substituted theophyllines (**1a**–**1e**) were obtained in high yields (81–93%).

1,3-Dimethyl-7-(2-(trifluoromethyl)benzyl)-3,7-dihydro-1*H*-purine-2,6-dione (**1a**)

1,3-Dimethyl-3,7-dihydro-1*H*-purine-2,6-dione, K_2_CO_3_, 2-(trifluoromethyl)benzyl chloride, DMF (10 mL). Product: 0.467 g, yield 92% (white solid). M.p.: 214–215 °C. ^1^H NMR (400 MHz, CDCl_3_, 25 °C, TMS): δ (ppm) = 3.42 (s, 3H, N-CH_3_); 3.63 (s, 3H, N-CH_3_); 5.78 (s, 2H, ArCH_2_-); 7.14 (d, *J* = 7.6 Hz, 1H, H-5′); 7.44–7.56 (m, 2H, H-4′ and H-6′); 7.53 (s, 1H, H-8); 7.76 (d, *J* = 7.2 Hz, 1H, H-3′). ^13^C NMR (101 MHz, CDCl_3_, 25 °C, TMS): δ (ppm) = 28.0 (N-CH_3_); 29.8 (N-CH_3_); 46.4 (q, *J =* 3.0 Hz, ArCH_2_-); 107.1 (C-5); 124.1 (q, ^1^*J*_CF_ = 273.8 Hz, CF_3_); 127.8 (q, ^2^*J*_CF_ = 30.7 Hz, C-2′); 128.6 (C-5′); 129.0 (C-6′); 132.8 (q, ^4^*J*_CF_ = 0.7 Hz, C-4′); 133.9 (q, ^3^*J_CF_* =1.4 Hz, C-1′ and C-3′); 141.5 (C-8); 148.8 (C-4); 151.7 (C-2); 155.3 (C-6). ^19^F NMR (376 MHz, CDCl_3_, 25 °C, TMS): δ (ppm) = −59.50 (CF_3_). FT-IR (ATR, cm^−1^): 3108 (=C-H), 2953 (C-H), 1704 (C=O), 1650 (C=O), 1550 (C=N), 1426 (C-H). Elemental analysis calcd. (%) for C_15_H_13_F_3_N_4_O_2_: C 53.26, H 3.87, N 16.56; found (%): C 53.55, H 3.99, N 16.44. (For the ^19^F NMR, ^1^H NMR, ^13^C NMR, and FT-IR spectrum of the compound **1a**, see Supplementary Materials, Figures S1 and S2).

1,3-Dimethyl-7-(3-(trifluoromethyl)benzyl)-3,7-dihydro-1*H*-purine-2,6-dione (**1b**)

The compound **1b** was prepared as previously reported in the literature [41]. 1,3-Dimethyl-3,7-dihydro-1*H*-purine-2,6-dione, K_2_CO_3_, 3-(trifluoromethyl)benzyl chloride, DMF (10 mL). Product: 0.411 g, yield 81% (white solid). M.p.: 128–129 °C. ^1^H NMR (400 MHz, CDCl_3_, 25 °C, TMS): δ (ppm) = 3.42 (s, 3H, N-CH_3_); 3.61 (s, 3H, N-CH_3_); 5.58 (s, 2H, ArCH_2_); 7.49–7.63 (m, 3H, H-2′, H-4′, H-6′); 7.58 (s, 1H, H-8); 7.64 (s, 1H, H-5′). ^13^C NMR (101 MHz, CDCl_3_, 25 °C, TMS): δ (ppm) = 27.0 (N-CH_3_); 28.8 (N-CH_3_); 48.6 (ArCH_2_); 105.8 (C-5); 121.3 (q, ^1^*J_C_*_F_ = 272.5 Hz, CF_3_), 123.5 (q, ^3^*J_C_*_F_ = 3.8 Hz, C-4′); 124.5 (q, ^3^*J_C_*_F_ = 3.8 Hz, C-2′); 128.7 (C-6′); 130.3 (q, ^4^*J_C_*_F_ = 1.0 Hz, C-5′); 130.5 (q, ^2^*J_C_*_F_ = 32.6 Hz, C-3′); 135.5 (C-1′); 139.8 (C-8); 147.9 (C-4); 150.6 (C-2); 154.2 (C-6). ^19^F NMR (376 MHz, CDCl_3_, 25 °C, TMS): δ (ppm) = −62.67 (CF_3_). FT-IR (ATR, cm^−1^): 3107 (=C-H), 2955 (C-H), 1694 (C=O), 1644 (C=O), 1547 (C=N), 1456 (C-H). Elemental analysis calcd. (%) for C_15_H_13_F_3_N_4_O_2_: C 53.26, H 3.87, N 16.56; found (%): C 53.41, H 4.02, N 16.39. (For the ^19^F NMR, ^1^H NMR, ^13^C NMR, and FT-IR spectrum of the compound **1b**, see Supplementary Materials, Figures S3 and S4).

1,3-Dimethyl-7-(4-(trifluoromethyl)benzyl)-3,7-dihydro-1*H*-purine-2,6-dione (**1c**)

The compound **1c** was prepared as previously reported in the literature [42]. 1,3-Dimethyl-3,7-dihydro-1*H*-purine-2,6-dione, K_2_CO_3_, 4-(trifluoromethyl)benzyl chloride, DMF (10 mL). Product: 0.441 g, yield 87% (white solid). M.p.: 196–197 °C. ^1^H NMR (400 MHz, CDCl_3_, 25 °C, TMS): δ (ppm) = 3.32 (s, 3H, N-CH_3_); 3.52 (s, 3H, N-CH_3_); 5.49 (s, 2H, ArCH_2_); 7.36 (d, *J* = 8.1 Hz, 2H, H-2′, H-6′); 7.55 (d, *J* = 8.1 Hz, 2H, H-3′, H-5′); 7.56 (s, 1H, H-8). ^13^C NMR (101 MHz, CDCl_3_, 25 °C, TMS): δ (ppm) = 28.0 (N-CH_3_); 29.8 (N-CH_3_); 49.7 (ArCH_2_); 106.9 (C-5); 123.8 (q, ^1^*J_C_*_F_ = 272.3 Hz, CF_3_); 126.1 (q, ^3^*J_C_*_F_ = 3.7 Hz, C-3′ and C-5′); 128 (C-2′ and C-6′); 130.9 (q, ^2^*J_C_*_F_ = 32.6 Hz, C-4′); 139.4 (C-1′); 140.9 (C-8); 149.0 (C-4); 151.6 (C-2); 155.2 (C-6). ^19^F NMR (376 MHz, CDCl_3_, 25 °C, TMS): δ (ppm) = −62.75 (CF_3_). FT-IR (ATR, cm^−1^): 3093 (=C-H), 2893 (C-H), 1703 (C=O), 1654 (C=O), 1548 (C=N), 1472 (C-H). Elemental analysis calcd. (%) for C_15_H_13_F_3_N_4_O_2_: C 53.26, H 3.87, N 16.56; found (%): C 53.34, H 3.93, N 16.52. (For the ^19^F NMR, ^1^H NMR, ^13^C NMR, and FT-IR spectrum of the compound **1c**, see Supplementary Materials, Figures S5 and S6).

1,3-Dimethyl-7-(3,5-bis(trifluoromethyl)benzyl)-3,7-dihydro-1*H*-purine-2,6-dione (**1d**)

1,3-Dimethyl-3,7-dihydro-1*H*-purine-2,6-dione, K_2_CO_3_, 3,5-bis(trifluoromethyl)benzyl bromide, DMF (10 mL). Product: 0.567 g, yield 93% (white solid). M.p.: 157–158 °C. ^1^H NMR (400 MHz, CDCl_3_, 25 °C, TMS): δ (ppm) = 3.40 (s, 3H, N-CH_3_); 3.61 (s, 3H, N-CH_3_); 5.63 (s, 2H, ArCH_2_); 7.69 (s, 1H, H-8); 7.79 (s, 2H, H-2′, H-6′); 7.86 (s, 1H, H-4′). ^13^C NMR (101 MHz, CDCl_3_, 25 °C, TMS): δ (ppm) = 28.0 (N-CH_3_); 29.9 (N-CH_3_); 49.2 (ArCH_2_); 106.7 (C-5); 122.9 (q, ^1^*J_C_*_F_ = 273 Hz, CF_3_); 122.7 (hept., ^3^*J_C_*_F_ = 3.8 Hz, C-4′); 128 (q, ^3^*J_C_*_F_ = 2.7 Hz, C-2′ and C-6′); 132.6 (q, ^2^*J_C_*_F_ = 33.7 Hz, C-3′ and C-5′); 138.1 (C-1′); 140.7 (C-8); 149.1 (C-4); 151.6 (C-2); 155.2 (C-6). ^19^F NMR (376 MHz, CDCl_3_, 25 °C, TMS): δ (ppm) = −62.90 (CF_3_). FT-IR (ATR, cm^−1^): 3107 (=C-H), 2957 (C-H), 1703 (C=O), 1645 (C=O), 1551 (C=N), 1471 (C-H). Elemental analysis calcd. (%) for C_16_H_12_F_6_N_4_O_2_: C 47.30, H 2.98, N 13.79; found (%): C 47.48, H 3.11, N 13.76. (For the ^19^F NMR, ^1^H NMR, ^13^C NMR, and FT-IR spectrum of the compound **1d**, see Supplementary Materials, Figures S7 and S8).

1,3-Dimethyl-7-(4-(trifluoromethoxy)benzyl)-3,7-dihydro-1*H*-purine-2,6-dione (**1e**)

1,3-Dimethyl-3,7-dihydro-1*H*-purine-2,6-dione, K_2_CO_3_, 4-(trifluoromethoxy)benzyl bromide, DMF (10 mL). Product: 0.478 g, yield 90% (white solid). M.p.: 177–178 °C. ^1^H NMR (400 MHz, CDCl_3_, 25 °C, TMS): δ (ppm) = 3.34 (s, 3H, N-CH_3_); 3.52 (s, 3H, N-CH_3_); 5.43 (s, 2H, ArCH_2_); 7.14 (d, *J* = 8.0 Hz, 2H, H-3′, H-5′); 7.31 (d, *J* = 8.0 Hz, 2H, H-2′, H-6′); 7.54 (s, 1H, H-8). ^13^C NMR (101 MHz, CDCl_3_, 25 °C, TMS): δ (ppm) = 28.0 (N-CH_3_); 29.8 (N-CH_3_); 49.5 (ArCH_2_); 106.9 (C-5); 120.4 (q, ^1^*J_C_*_F_ = 258.6 Hz, OCF_3_); 121.5 (C-3′ and C-5′); 129.5 (C-2′ and C-6′); 134.1 (C-1′); 149.3 (q, ^3^*J*_CF_ = 2.0 Hz, C-4′); 140.7 (C-8); 148.9 (C-4); 151.6 (C-2); 155.3 (C-6). ^19^F NMR (376 MHz, CDCl_3_, 25 °C, TMS): δ (ppm) = −57.87 (OCF_3_). FT-IR (ATR, cm^−1^): 3103 (=C-H), 2950 (C-H), 1703 (C=O), 1656 (C=O), 1547 (C=N), 1423 (C-H). Elemental analysis calcd. (%) for C_15_H_13_F_3_N_4_O_3_: C 50.85, H 3.70, N 15.81; found (%): C 50.99, H 3.82, N 15.84. (For the ^19^F NMR, ^1^H NMR, ^13^C NMR, and FT-IR spectrum of the compound **1e**, see Supplementary Materials, Figures S9 and S10).

### 2.2. Molecular Docking Method

All the molecules were optimized with the ORCA package program version 4.0 by using BP86 functional with a def2-TZVP, def2-TZVP/j basis set, tightscf, and grid4 restrictions before the docking performances [43,44]. The docking calculations were performed against VEGFR-2 (pdb:1ywn) [45], Human Cytochrome P450 (pdb:3ruk) [46], Estrogen Receptor (pdb:3ert) [47], DNA Gyrase (pdb:1kzn) [48], and SarA (pdb:2fnp) [49] with AutoDockTools 4.2 [50]. RCSB protein data bank (https://www.rcsb.org/) was used for downloading the crystal structures of target molecules. All the target molecules and molecules were saved in pdbqt format with the assistance of AutodockTools 4.2 [51]. Kollman charges were considered for target molecules with only polar hydrogens and the water in the macromolecules were removed. Genetic algorithm population was considered as Lamarckian Genetic Algorithms as 150. The performances started with random position and Gasteiger charges were considered for ligands [52]. All the illustrations were also performed with Discovery Studio 4.1.0.

### 2.3. Evaluation of Anticancer Activity and Selectivity Index

The anticancer potential of substituted theophylline derivatives was investigated using human lung carcinoma (A549, CCL-185) and cervical cancer (HeLa, CCL-2) cell lines obtained from the American Type Culture Collection (ATCC). To evaluate the selectivity of the tested compounds, cytotoxicity was also assessed in a healthy human bronchial epithelial cell line (BEAS-2B, ATCC CRL-9609). Cells were maintained in DMEM (Euroclone S.p.A., Pero, Italy supplemented with 10% fetal bovine serum (Sigma-Aldrich, St. Louis, MO, USA) and 1% penicillin–streptomycin (1%, Capricorn Scientific GmbH, Ebsdorfergrund, Germany at 37 °C in a humidified atmosphere containing 5% CO_2_. The theophylline-derived compounds (**1a**–**1e**) were dissolved in DMSO to prepare 100 mM stock solutions, which were then diluted with DMEM to obtain final working concentrations ranging from 3.125 µM to 100 µM.

To evaluate cell viability, the MTT colorimetric assay was employed. Upon reaching logarithmic growth, A549, HeLa and BEAS-2B cells were seeded at a density of 7000 cells per well in 96-well microplates and allowed to attach for 24 h. Following this, cells were treated with different concentrations of the compounds and incubated for 48 h.

After treatment, 100 µL of fresh medium and 20 µL of MTT reagent (5 mg/mL, Bio Basic Inc., Markham, ON, Canada) were added to each well, and cells were incubated for an additional 3 h. The resulting formazan crystals were solubilized with DMSO. Cell viability was calculated as the percentage of absorbance relative to untreated controls, and IC_50_ values were determined by nonlinear regression analysis using GraphPad Prism software, version 8.0 (GraphPad Software, San Diego, CA, USA. All experiments were performed in at least three independent replicates, and results are reported as mean ± SD.

The percentage of inhibition was calculated based on the reduction in metabolic activity of the cells following compound treatment, as assessed by the MTT assay. The absorbance was measured at 570 nm using a microplate reader. The inhibition (%) was calculated using the following formula:

*Inhibition* %=[1 − (*A_sample_* − *A_blank_*)/(*A_sample_* − *A_blank_*)] × 100

To assess the tumor selectivity of the tested theophylline derivatives, the selectivity index (SI) was calculated for each compound. SI is defined as the ratio of the IC_50_ value in healthy (non-tumorigenic) cells to the IC_50_ value in cancer cells. In this study, IC_50_ values were determined for BEAS-2B (non-tumorigenic human bronchial epithelial cells) as a healthy control and for A549 (lung adenocarcinoma) and HeLa (cervical carcinoma) as tumor models. The SI was calculated according to the following equation:

SI = [IC_50_, healthy/IC_50_, cancer]

SI value greater than 2 is generally considered indicative of preferential cytotoxicity toward cancer cells and is widely used as a benchmark for tumor selectivity in anticancer drug discovery.

### 2.4. Gene Expression Analysis by RT-qPCR

To elucidate the molecular mechanisms underlying the anticancer activity of the most potent compound (**1a**), quantitative real-time PCR (RT-qPCR) was performed to analyze gene expression profiles in A549 and HeLa cells. Both cell lines were treated with compound **1a** at IC_50_ concentrations for 48 h. Total RNA was isolated from cells using the RNeasy Mini Kit (Qiagen GmbH, Hilden, Germany) according to the manufacturer’s instructions. RNA quality and quantity were evaluated spectrophotometrically. Subsequently, cDNA synthesis was performed using the iScript™ cDNA Synthesis Kit (Bio-Rad Laboratories, Hercules, CA, USA).

RT-qPCR reactions (LightCycler® 480 Instrument II (Roche Diagnostics, Mannheim, Germany) were carried out using SYBR™ Green PCR Master Mix (Applied Biosystems, Foster City, CA, USA) and gene-specific primers targeting pro-apoptotic genes (CASP3, CASP8, CASP9, BAX, BAK1, PUMA, NOXA, CYCS), anti-apoptotic genes (BCL2, BCL-XL, MCL1), cell cycle/stress-related genes (TP53, CDKN1A/p21, GADD45A, ATF4), and the reference gene GAPDH. The primer sequences used for each gene are presented in the Supplementary File (Figure S66).

All reactions were conducted in triplicate, and negative controls (no-template controls, NTC) were included in each run. Relative gene expression levels were analyzed using the 2^−ΔΔCt^ method, and results were normalized to control samples (control expression set as ‘1’) and expressed as fold change relative to controls.

### 2.5. Quantification of Apoptosis-Related Proteins by ELISA

To validate the gene expression changes observed in the RT-qPCR analysis at the protein level, ELISA assays were conducted to measure the protein levels in A549 and HeLa cells. Cells were treated with compound 1a at its IC_50_ concentration for 48 h. Following incubation, cells were washed with cold PBS and lysed using RIPA buffer supplemented with protease and phosphatase inhibitors. Cell was removed by centrifugation at 14,000× *g* for 10 min.

Total protein concentration in the cell lysates was determined spectrophotometrically using the BCA Protein Assay Kit (Thermo Scientific, Waltham, MA, USA). Protein concentrations were standardized to equal amounts across all samples prior to ELISA analysis. Commercially available Human ELISA Kit (Abcam, Cambridge, UK) were used to quantify Caspase-3, BAX, BCL-2, and Cytochrome C levels, and all assays were performed according to the manufacturers’ protocols.

Absorbance was measured at 450 nm using a microplate reader. All experiments were conducted in triplicate. The results were normalized to the control group (control = 1) and expressed as fold change relative to untreated controls.

### 2.6. Determination of Antimicrobial Activity

The antibacterial activities of the compounds were determined using the broth microdilution method, as per the advice of international standards [53,54]. The minimum inhibitory concentration (MIC) values of the compounds **1a**–**1e** for the yeast and bacterial strains were investigated using the method. Standard bacterial strains *Staphylococcus aureus* (ATCC 29213), *Enterococcus faecalis* (ATCC 29212), *Escherichia coli* (ATCC 25922), *Pseudomonas aeruginosa* (ATCC 27853), and yeast *Candida albicans* (ATCC 10231), were previously stored at −80 °C then revived at room temperature. Microorganisms were inoculated using overnight broth cultures. Stock solutions of the samples studied at a concentration of 1000 µg/mL were prepared with dimethyl sulfoxide (DMSO). An amount of 100 µL of Mueller Hinton Broth (Liofilchem, Italy) were dispensed into all wells of 96-well microplates. MIC range was set as 0.244–1000 µg/mL by adding 1000 µg/mL serial dilutions of the prepared solutions to the first wells of the microplates. Serial dilutions of 0.00048–1% DMSO were made into a column of the microplate to determine its antimicrobial activity. 0.5 McFarland standard in physiological saline from each microorganism strains were prepared and 20 µL of the prepared suspension was added to the wells. An amount of 100 µL Mueller Hinton Broth in a column of the microplate served as a negative control and another column of the microplate containing 100 µL of Mueller Hinton Broth with 20 µL of each microorganism added served as a growth control. As positive controls, ciprofloxacin was applied against bacterial strains at a concentration range of 0.03125–64 µg/mL, and fluconazole was used against *Candida albicans* at a concentration range of 0.3125–640 µg/mL. The plates were covered with parafilm, incubated at 36.5 °C in an aerobic environment for 24 h. After incubation, the optical density was measured at 620 nm. The first concentration without microbial growth was determined as MIC and experiments were repeated twice.

### 2.7. Reduction in Biofilm Formation

The well plate assay was utilized to test the effectiveness of compounds in inhibiting biofilms made by microorganisms [53]. An amount of 100 µL of Brucella broth (Remel, Lenexa, KS, USA) containing 1% and 20% glucose (Biolife, Milan, Italy), were dispensed into the test wells. In the next step, 100 µL of compounds ranging from 0.244 to 1000 µg/mL were poured into the wells. Serial dilutions of 0.00048–1% DMSO were made into a column of the microplate to determine its antibiofilm activity. Then, 0.5 McFarland standard in physiological saline from each microorganism strain were prepared and 20 µL of the prepared suspension was added to the wells. A column of the microplate containing 100 µL Brucella broth served as a negative control and another column of the microplate containing 100 µL Brucella with 20 µL of each microorganism added served as a growth control. As positive controls, ciprofloxacin was applied against bacterial strains at a concentration range of 0.03125–64 µg/mL, and fluconazole was used against *Candida albicans* at a concentration range of 0.3125–640 µg/mL. Plates were left to incubate at 36.5 °C for a day. The plates were emptied and then washed three times with 300 µL of deionized water. The wells were left to dry for half an hour. The next step was staining with 0.1% (*w*/*v*) violet dye (Norateks, Türkiye), cleaning three times with deionized water, and drying at room temperature. The crystal violet adhering to the wall of the wells was dissolved with 95% ethanol, and the optical density was measured at 570 nm. The test was run two times, and the mean value was calculated. Biofilm preventing (%) = {(OD_b_ − OD_c_)/OD_b_} × 100.

## 3. Results and Discussion

### 3.1. Preparation and Characterization of N(7)-Substituted Theophyllines (***1a***–***1e***)

**Scheme 1:**
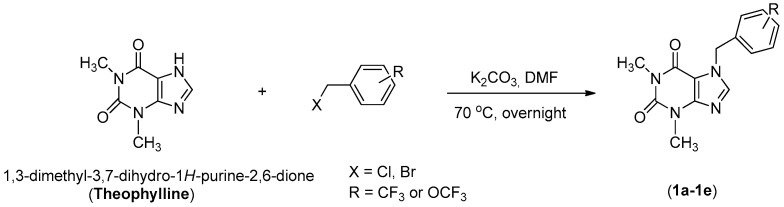

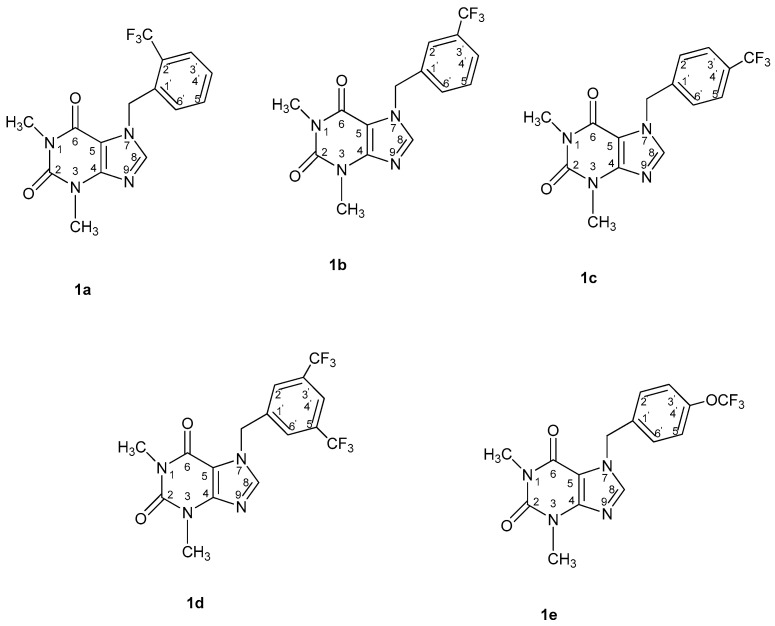
The general synthetic pathway and molecular structures of N(7)-substituted theophyllines **1a**–**1e**.

N(7)-substituted theophyllines (**1a**–**1e**) were prepared by the reaction of 1,3-dimethyl-3,7-dihydro-1*H*-purine-2,6-dione (theophylline) and trifluoromethyl substituted benzyl halide compounds. The synthesized N(7)-substituted theophyllines were obtained as white powder in high yield. The general synthetic pathway and molecular structures for the N(7)-substituted theophyllines are displayed in Scheme 1.

The structures of the synthesized N(7)-substituted theophyllines were fully characterized by ^1^H NMR, ^13^C NMR, ^19^F NMR, and FT-IR spectroscopy and elemental analysis. Some selected physical and spectroscopic data of the prepared compounds **1a**–**1e** are summarized in Table 1.

NMR spectroscopy is one of the most effective and useful methods for the characterization of organic compounds. ^1^H NMR, ^13^C NMR, and ^19^F NMR data for the characterization of the prepared compounds can be summarized as follows.

When the ^1^H NMR spectra of compounds **1a**–**1e** were examined, benzylic -CH_2_-Ph protons of trifluoromethyl substituted benzyl groups were observed as singlet peaks at δ = 5.78, 5.58, 5.59, 5.63, and 5.43 ppm, respectively. The presence of the CF_3_ group at the ortho-, meta-, or para- positions moves the signals of the -CH_2_-Ph protons to the downfield for compounds **1a**–**1c**. Methyl hydrogens at the N(1) and N(3) positions of the theophylline ring were observed as singlet peaks in the range δ = 3.32–3.63 ppm. Due to trifluoromethyl substituents at the ortho-, meta-, or para- positions of the substituted benzyl group, the expected signal cleavage at the down-field resonances for the aromatic ring hydrogens of the benzyl group were in agreement with the position of the trifluoromethyl substituent. The hydrogen of acidic character at the C(8)-position of theophylline was observed as singlet peaks at δ = 7.53, 7.58, 7.56, 7.69, and 7.54 ppm for compounds **1a**–**1e**, respectively. When the ^13^C NMR spectra of compounds **1a**–**1e** were examined, it was observed that the benzylic carbon -CH_2_-Ph resonances of the substituted benzyl group gave signals at δ = 46.44, 48.65, 49.68, 49.18, and 49.47 ppm, respectively. Carbons of the trifluoromethyl substituent in the benzyl group for the compounds **1a**–**1e** gave signals at δ = 133.95, 135.44, 139.41, 138.07, and 134.14 ppm, respectively. Methyl carbons at N(1) and N(3) positions of the theophylline ring gave a signal in the range of δ = 27.01–29.86 ppm. The signals of the carbonyl carbon at the C(2) and C(6) positions of the theophylline ring were observed approximately in the range of δ = 150.59–151.66 and 154.22–155.29 ppm for all compounds. The C(8) carbon resonance of the theophylline ring gave signals at δ = 141.50, 139.78, 140.88, 140.71, and 140.74 ppm for compounds **1a**–**1e**, respectively. In addition, in the ^19^F NMR spectra of compounds **1a**–**1e**, it was observed that the fluorine atoms of the trifluoromethyl substituent in the substituted benzyl group gave signals at δ = −59.50, −62.67, −62.75, −62.90, and −57.87 ppm, respectively.

In ^13^C NMR, characteristic signal splits originating from the CF_3_ group were observed as expected. The CF_3_ carbon in compounds **1a**–**1e** gave signals at δ = 124.1, 121.3, 123.8, 122.9, and 120.4 ppm, respectively, as quartets. The coupling constants for these signals were in the range of ^1^*J*_CF_ = 258–274 Hz. Similarly, the aromatic carbons bearing the CF_3_ group in the benzene ring were observed as quartets at δ = 127.8, 130.5, 130.9, and 132.6 ppm, respectively, for compounds **1a**–**1d**. The coupling constants for these signals were in the range of ^2^*J*_CF_ = 30–34 Hz. These quartet signals originating from the CF_3_ group support the proposed structure. Additionally, the data presented in previously reported studies in the literature are consistent with these characteristic signals [41,42]. (For further details, refer to the ^13^C NMR spectra of compounds **1a**–**1e** in the Supplementary Materials File Figures S11–S20)

FT-IR spectroscopy is another important characterization method used for the recognition of functional groups in organic synthesis. The data of the characterization of the functional groups of compounds **1a**–**1e** can be summarized as follows.

FT-IR spectra of the prepared compounds clearly showed that compounds **1a**–**1e** exhibited characteristic signals for their functional groups. For example, C(2)=O and C(6)=O carbonyl vibrations in the theophylline ring were observed as sharp signals between 1644 and 1704 cm^−1^ frequencies. However, the characteristic C(8)=N vibrational frequencies of compounds **1a**–**1e** gave sharp signals at 1550, 1547, 1548, 1551, and 1547 cm^−1^, respectively. The other signals observed in the FT-IR spectra are in agreement with the structures of compounds **1a**–**1e**.

Elemental analysis method was also used to confirm the proposed structure of the prepared compounds. The % components calculated for all compounds **1a**–**1e** and the % components found are in agreement with the proposed structures.

Compounds **1a**–**1e** were highly stable to air and moisture in the solid state. The solubility of these compounds in a number of solvents was also tested. They exhibited high solubility in a wide range of solvents of organic origin such as ethyl alcohol, isopropyl alcohol, dichloromethane, chloroform, acetonitrile, N-methylpyrrolidone, diethylcarbonate, dimethylformamide, dimethylsulfoxide, and toluene. In contrast, their solubility in diethyl ether was relatively poor. However, they were almost insoluble in apolar solvents such as *n*-pentane and *n*-hexane and in inorganic solvents such as water.

### 3.2. Molecular Docking

Molecular docking computationally predicts the orientations or binding modes of a molecule to a target macromolecule. This method contributes to drug discovery research by identification and optimization of drug candidates and can be used to predict the three-dimensional structure of protein–ligand complexes which experimentally are not available [55]. Additionally, the molecular docking can make estimations about how the structural changes in the small molecules alter the pharmaceutical properties. Therefore, the method not only accelerates the drug discovery studies but also provides useful insight into the molecular interaction of certain biological processes [56].

**Figure 2:**
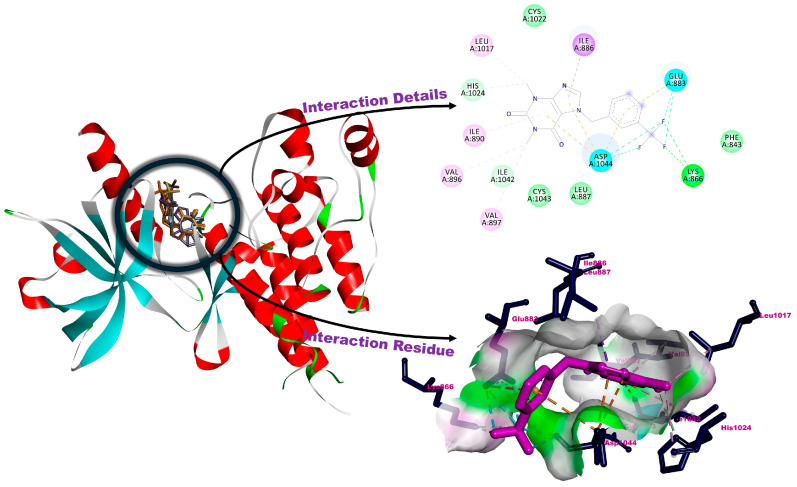
Interaction residue and details of **1b** against VEGFR−2.

Angiogenesis refers to the development and renewal of new vessels from pre-existing vessels. Physiological angiogenesis which has strongly occurred till adolescence, continues very slowly in adults during wound healing, menstrual cycles, and pregnancy. On the other hand, pathological angiogenesis can emerge in such inflammation, diabetic retinopathy, atherosclerosis, and tumor formation. One of the main regulators of angiogenesis is Vascular Endothelial Growth Factor (VEGF) [57]. The VEGF family, which consists of seven members and has a glycoprotein structure, exhibits its biological effects by binding to specific receptors. These receptors are transmembrane tyrosine kinase receptors and are called VEGFR-1, VEGFR-2, VEGFR-3, and neuropilin (NP-1 and NP-2). VEGFR-2 plays crucial roles in the proliferation and migration of endothelial cells, therefore it is an important target molecule for the suppression of angiogenesis in diseases such as cancer. VEGFR2 inhibitors are used in the recent treatment procedures of various types of cancer [58]. The studies for searching out new VEGFR-2 inhibitors are still ongoing. Al-Hazmy et al. analyzed the antiproliferative activity of some coumarin derivatives and confirmed their potential with molecular docking method. They used VEGFR-2 crystal structure as an indication of the activity against colon carcinoma cell lines, recorded H-bonds with Asn921, Cys917, and pi-interactions with Val914, Leu1038 [59]. Serdaroğlu et al., analyzed the anticancer activities of new benzimidazole-type N-heterocyclic carbene molecule and its silver complex in vitro and in silico methods including molecular docking. They evaluated the details by molecular docking method against VEGFR-2, which recorded stronger interactions for Ag(I)-NHC complex with −7.59 kcal/mol binding affinity than the precursor salt with the interactions through Asp1044, Glu883, Ile886, Val896, Cys1022, and His1024 [60]. In another recent study, Pinki and Chaudhary analyzed the anticancer activity of novel macrocyclic Zn(II) complexes, using VEGFR-2 target structure for analyzing drug candidates against breast and colon cancer cells. They recorded H-bonds with Lys866 in addition to hydrophobic interactions Leu838, Cys917, Arg1030, and Asp1044 [61]. In this study, in order to have an idea about the possible mechanisms of anticancer activity, the interactions of molecules with VEGFR-2 were analyzed by molecular docking method. All molecules interacted with the same region of the target molecule and this interaction residue is consistent with the reference molecule 4-amino-furo [2,3-d]pyrimidine (AAFP) and previous studies. Root mean-square distance (RMSD) of the obtained poses was recorded ≤2 Å [62]. The overlaps of the determined poses for compounds **1a**–**1e** are given in Supplementary Materials File Figures S22, S23, S25, S27, and S29. AAFP was used as a positive control molecule, −5.61 kcal/mol binding constant was determined with H-bonds through Glu883, Glu915, Cys917, and Asp1044 in addition to many other interactions presented in Table 2. H-bonds were detected for all molecules. The highest interaction was recorded as −5.69 kcal/mol for 1b. H-bonds with Lys866, His1024, and Ile1042, pi-interaction with Ile886, and alkylic interactions with Ile890, Val896, Val897, and Leu1017, in addition to van der Waals interactions, can contribute to the calculated binding energy of **1b**. Additionally, the contributions of the halogenic interactions of CF_3_ substituents should also be remarkable. According to the calculated binding affinity values, the possible effects of the molecules can occur in the order **1b** > **1e** > **1c** > **1d** > **1a**. All the details of the molecular docking performances are presented in Table 2. The interaction residue and details of compound **1b** against VEGFR−2 are shown in Figure 2. (For the interaction residue and details of other compounds against VEGFR−2, see Supplementary Materials File Figures S21, S24, S26, and S28).

**Figure 3:**
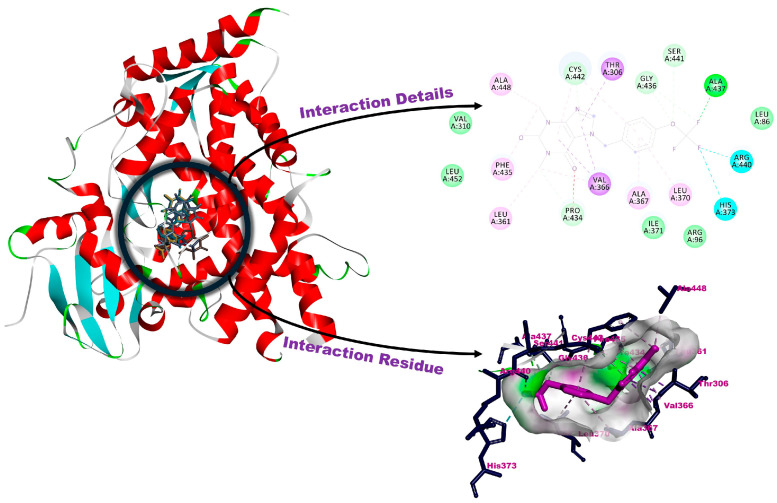
Interaction residue and details of **1e** against Human Cytochrome P450.

Cytochrome 450 (CYPs) is an enzyme superfamily involved in the metabolism of xenobiotics such as drugs, industrial chemicals, pesticides. Many macromolecules such as steroids, prostaglandins, and fatty acids are physiological substrates of these Cytochrome 450-type enzymes [63]. The changes in the expression levels of these enzymes due to gene polymorphisms and structural changes may affect the metabolism of xenobiotics and the tolerance against these xenobiotics. P450 17α-hydroxylase, which is a member of CYP family, catalyzes androgen biosynthesis in humans and therefore it is an important target molecule for the treatment of ovarian, colorectal, breast, and prostate cancers [64]. Dhawale et al. analyzed the activity of some phytoconstituents and enzymes against CYP450 by molecular docking method. They recorded good interactions for Peonidin, Pelargonidin, Malvidin, and Berberine and the binding affinity was recorded as −7.574 kcal/mol with remarkable H-bonding interactions [65]. Bahzad et al. analyzed the Cu(II) complexes with new mixed ligands for anticancer potential and confirmed the activity by molecular docking method against CYP450. They recorded a broad range binding affinity of −5.60 kcal/mol and −7.91 kcal/mol [66]. Tajiani et al. performed molecular docking for some natural flavanols on PC-3 cell line with CYP450 crystal structure. Abiraterone had the best binding properties with −10.3 kcal/mol with the residue including Val483, Glu305, Arg239, and Cys442 [67]. In this study, with an idea about the possible mechanisms of anticancer activity, the interactions of molecules against Human Cytochrome P450 were analyzed by molecular docking method. All molecules interacted with the same region of the target molecule and this interaction residue is consistent with the reference molecule Abiraterone and previous studies. Root mean-square distance (RMSD) of the obtained poses was recorded as ≤2 Å. The overlaps of the determined poses for compounds **1a**–**1e** are given in Supplementary Materials File Figures S31, S33, S35, S37, and S39. Abiraterone was used as a positive control molecule, −9.01 kcal/mol binding constant was determined with H-bond through Asn202 in addition to many other interactions presented in Table 2. H-bonds were detected for all molecules. The highest interaction was recorded as −6.69 kcal/mol for **1e**. H-bonds with Pro434, Gly436, Ala437, Ser441, and Cys442, pi-interactions with Thr306 and Val366, alkylic interactions with Leu361, Ala367, Leu370, Phe435, and Ala448, in addition to van der Waals interactions, can contribute to the calculated binding energy of **1e**. Additionally, the contributions of the halogenic interactions of CF_3_ substituents should also be remarkable. According to the calculated binding affinity values, the possible effects of the molecules can occur in the order **1e** > **1c** > **1b** > **1a** > **1d**. All the details of the molecular docking performances are presented in Table 2. The interaction residue and details of compound **1e** against Human Cytochrome P45 are shown in Figure 3. (For the interaction residue and details of other compounds against Human Cytochrome P45, see Supplementary Materials File Figures S30, S32, S34, S36, and S38).

**Figure 4:**
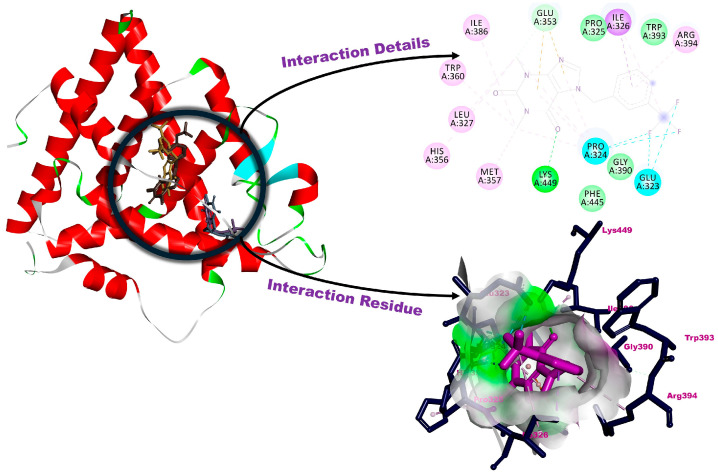
Interaction residue and details of **1b** against estrogen receptor.

Estrogen receptors, members of the nuclear receptor superfamily, are specialized cell surface proteins. Estrogen hormones bind to these receptors to exert their physiological effects. In this way, the differentiation and maintenance of nervous, skeletal, cardiovascular, and reproductive tissues are regulated. Since many breast cancer tumors have estrogen receptors, the growth of these tumors is supported by estrogen. Therefore, modulation of these receptors can be used as a treatment procedure in breast cancer cells. A similar treatment procedure is also used in the treatment of some osteoporosis and cardiovascular diseases [68]. As example, Kumar et al. synthesized new quinoline derivative molecules for possible activity against breast cancer cells and analyzed the details against estrogen receptors. They recorded docking score ranges between −8.04 and −9.39 kcal/mol and determined hydrogen bonds on amino acid residue including Thr347, Glu353, and Arg394 [69]. In another study, Sarkar and Maiti analyzed the organosulfur and the flavonoid ingredients of garlic for possible therapy against Estrogen Receptor with molecular docking methods. Among the flavonoids, they recorded the best binding affinity for kaempferol as −8.0 kcal/mol [70]. In a recent study, Sehrawat et al. analyzed several chlorogenic acid derivatives for possible treatment by in silico methods including molecular docking. Most active ligand showed hydrogen bonds with Asp351 in addition to pi-interactions with Tyr526 and many hydrophobic interactions [71]. In this study, with an idea about the possible mechanisms of anticancer activity, the interactions of molecules against Estrogen Receptor were analyzed by molecular docking method. All molecules interacted with the same region of the target molecule and this interaction residue is consistent with the reference molecule 4-Hydroxytamoxifen (HT) and previous studies. Root mean-square distance (RMSD) of the obtained poses was recorded as ≤2 Å. The overlaps of the determined poses for compounds **1a**–**1e** are given in Supplementary Materials File Figures S40, S41, S43, S45, S46, and S47. Hydroxytamoxifen was used as a positive control molecule, −10.35 kcal/mol binding constant was determined with H-bond through Asp351, Glu353, and Arg394 in addition to many other interactions presented in Table 2. H-bonds were detected for all molecules. The highest interaction was recorded as −6.70 kcal/mol for **1b**. H-bonds with Glu353 and Lys449, pi-interaction with Ile326, and alkylic interactions with Leu327, His356, Met357, Trp360, Ile386, and Arg394, in addition to van der Waals interactions, can contribute to the calculated binding energy of 1b. Additionally, the contributions of the halogenic interactions of CF3 substituents should also be remarkable. According to the calculated binding affinity values, the possible effects of the molecules can occur in the order **1b** > **1a** > **1e** > **1d** > **1c**. All the details of the molecular docking performances are presented in Table 2. The interaction residue and details of compound **1b** against estrogen receptor are shown in Figure 4. (For the interaction residue and details of other compounds against estrogen receptor, see Supplementary Materials File Figures S39, S42, S44, and S46).

**Figure 5:**
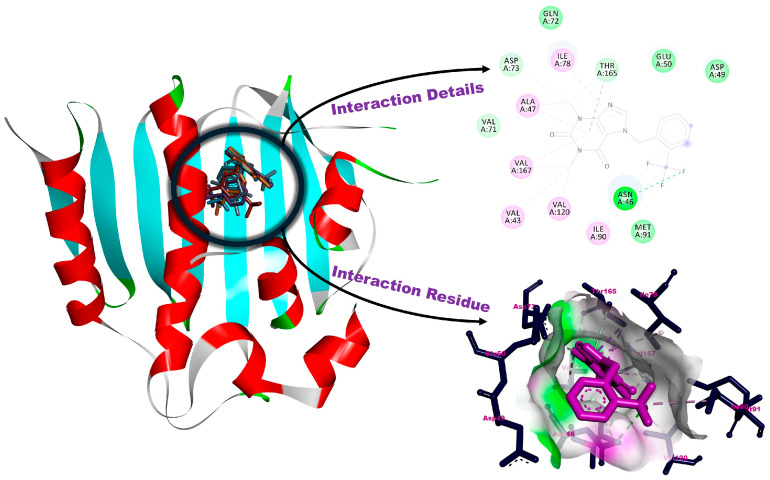
Interaction residue and details of **1a** against DNA Gyrase.

DNA gyrase is a Type II topoisomerase and affects DNA replication and maintenance of bacteria. This enzyme both introduces negative supercoils into DNA and relieves positive supercoils from the replication fork. Since it is crucial for the survival of bacteria, some antibacterial agents such as fluoroquinolones and aminocoumarins try to breach the action mechanism of this enzyme [72]. The studies for searching out new DNA Gyrase inhibitors are still ongoing. In a recent study, Bhukal et al. evaluated pyrazoline Spiro-oxindole tethered 1,2,3-triazole molecules and recorded H-bond with Asn46, pi-interactions with Ile78, Ile90, and some hydrophobic interactions with Glu50, Arg76, and Thr165 [73]. Üstün et al. analyzed a new series of silver complexes for their antimicrobial activity while they detailed the interaction against DNA Gyrase by molecular docking method. The binding energies were determined in the range −6.99 to −8.53 kcal/mol and the strongest interacted molecules had H-bond with Ala96 in addition to pi-interactions with Ile78, Ile90, Asn46, and many others such as alkylic and van der Waals interactions [74]. In another study, Gowthami et al. green synthesized by using *Mollugo oppositifolia* extract and characterized CeO_2_ NPs for analyzing the antimicrobial activity. They also detailed the molecular docking method against DNA Gyrase and determined −8.3 kcal/mol with H-bonds with His95, Ala96, and Val120 [75]. In this study, having an idea about the possible mechanisms of antibacterial activity, the interactions of molecules against DNA Gyrase were analyzed by molecular docking method. All molecules interacted with the same region of the target molecule and this interaction residue is consistent with the reference molecule Clorobiocin and previous studies. Root mean-square distance (RMSD) of the obtained poses was recorded as ≤2 Å. The overlaps of the determined poses for compounds **1a**–**1e** are given in Supplementary Materials File Figures S48, S50, S52, S54, and S56. Clorobiocin was used as a positive control molecule, −10.35 kcal/mol binding constant was determined with H-bond through Asn46, Asp73, and Arg136 in addition to many other interactions presented in Table 2. H-bonds were detected for all molecules. The highest interaction was recorded as −5.56 kcal/mol for **1a**. H-bonds with Asn46, Val71, Asp73, and Thr165, alkylic interactions with Val43, Ala47, Ile78, Ile90, Val120, and Val167, in addition to van der Waals interactions, can contribute to the calculated binding energy of **1a**. According to the calculated binding affinity values, the possible effects of the molecules can occur in the order **1a** > **1e** > **1b** > **1c** > **1d**. All the details of the molecular docking performances are presented in Table 2. The interaction residue and details of compound **1a** against DNA Gyrase are shown in Figure 5. (For the interaction residue and details of other compounds against DNA Gyrase, see Supplementary Materials File Figures S49, S51, S53, and S55).

**Figure 6:**
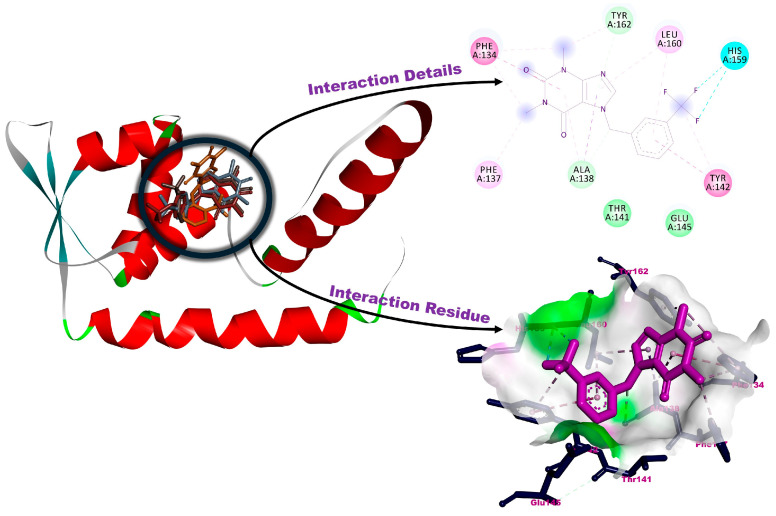
Interaction residue and details of **1b** against SarA.

*Staphylococcus aureus* which can cause a wide spectrum of infections such as pneumonia, endocarditis, and sepsis is a versatile bacterium. SarA (Staphylococcal accessory regulator A) is a global regulatory protein in *Staphylococcus aureus*. SarA not only regulates virulence factors but also promotes biofilm development. Additionally, SarA contributes to the antibiotic resistance and modulates bacterial communication by interaction with quorum-sensing system [76]. All these functions make SarA an important target for antibiotic design studies. The antimicrobial activity of the new 5,6-dimethylbenzimidazolium ligand and their silver complexes were analyzed, and the interaction details were also analyzed molecular docking methods against SarA. They recorded H-bonds with Lys123 and Tyr162 for silver complexes while Asn212, Tyr162, and Thr141 had the better activity for interaction with the benzimidazole-type precursors [77]. In another study, Kathiresan et al. determined the Anti-quorum sensing mediated anti-infective efficacy of pentadecanoic acid in vitro and in silico method. Their molecular docking performance revealed the H-bond with Asn212 while many other interactions were recorded with the certain residue of macromolecule including Lys123, Ile126, Phe137, and Ile215 [78]. In a very recent study, the essential oil from Artemisia herba-alba analyzed for possible bioactive ingredients and the antimicrobial activity of each sample were analyzed with molecular docking method. They presented the best score for α-thujone and columellarin as −7.5 kcal/mol and −7.2 kcal/mol with the H-bonds with Asn146, His159, and Tyr142, respectively [79]. In this study, with an idea about the possible mechanisms of antibacterial and anti-biofilm activity, the interactions of molecules against SarA were analyzed by molecular docking method. All molecules interacted with the same region of the target molecule and this interaction residue is consistent with previous studies. Root mean-square distance (RMSD) of the obtained poses was recorded as ≤2 Å. The overlaps of the determined poses for compounds **1a**–**1e** are given in Supplementary Materials File Figures S58, S59, S61, S63, and S65. H-bonds were detected for all molecules. The highest interaction was recorded as −4.90 kcal/mol for **1b**. H-bonds with Ala138, Tyr162, pi-interactions with Phe134, Tyr142, alkylic interactions with Phe137 and Leu160, in addition to van der Waals interactions, can contribute to the calculated binding energy of **1b**. Additionally, the contributions of the halogenic interaction of CF_3_ substituents should also be remarkable. According to the calculated binding affinity values, the possible effects of the molecules can occur in the order **1b** > **1c** > **1d** > **1a** > **1e**. All the details of the molecular docking performances are presented in Table 2. The interaction residue and details of compound **1b** against SarA are shown in Figure 6. (For the interaction residue and details of other compounds against SarA, see Supplementary Materials File Figures S57, S60, S62, and S64).

### 3.3. Anticancer Activity and Selectivity of Substituted Theophylline Derivatives

Theophylline derivatives have garnered attention in recent years due to their potential therapeutic efficacy against various cancer cell lines. This study investigates the anticancer properties and selectivity profiles of compounds **1a**–**1e** containing substituted theophylline ligands on A549 and HeLa cancer cells, as well as BEAS-2B non-tumorigenic bronchial epithelial cells.

**Figure 7:**
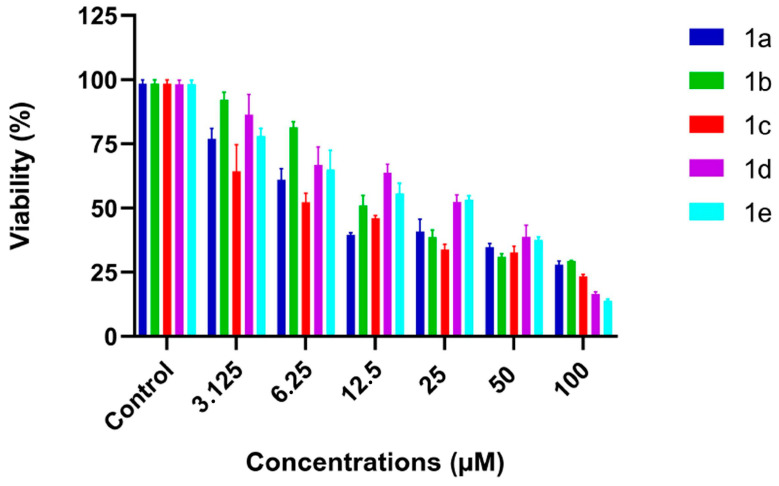
Cell viability of Hela cells treated with theophylline derivatives (**1a**–**1e**) for 48 h.

**Figure 8:**
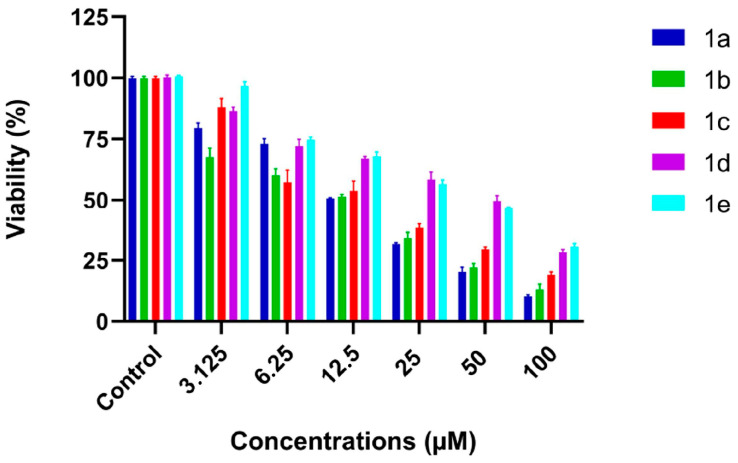
Cell viability of A549 cells treated with theophylline derivatives (**1a**–**1e**) for 48 h.

To evaluate the cytotoxic potential of the compounds in a dose-dependent manner, MTT assays were performed following 48-h incubation periods at concentrations ranging from 3.125 μM to 100 μM. IC_50_ values were subsequently calculated (Table 3). In HeLa cells, compounds **1a**, **1b**, and **1c** exhibited significantly greater cytotoxicity than compounds **1d** and **1e**, with IC_50_ values of 11.5 ± 1.2 μM, 10.8 ± 0.8 μM, and 14.7 ± 0.7 μM compared to 32.4 ± 0.85 μM and 34.5 ± 1.25 μM, respectively (Figure 7). These compounds displayed clear dose-dependent cytotoxic activity. Similarly, in A549 cells, IC_50_ values were recorded as 15.7 ± 0.6 μM, 12.4 ± 0.7 μM, and 13.6 ± 0.9 μM for compounds **1a**, **1b**, and **1c**, respectively, while compounds **1d** and **1e** showed reduced cytotoxicity with IC_50_ values of 29.1 ± 0.5 μM and 31.4 ± 1.0 μM (Figure 8).

**Figure 9:**
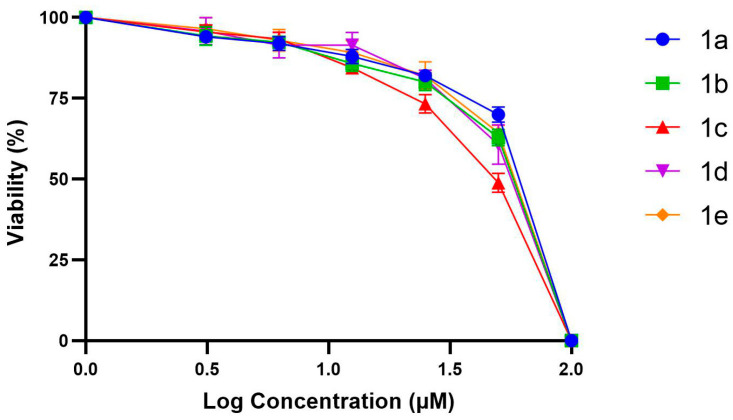
Cell viability of BEAS-2B cells treated with theophylline derivatives (**1a**–**1e**) for 48 h.

Selectivity index (SI) analyses were performed by comparing cytotoxic effects on non-tumorigenic BEAS-2B cells. All tested compounds demonstrated substantially lower cytotoxicity in BEAS-2B cells compared to A549 and HeLa cancer cells, reflected by high SI values (>4). Notably, compound 1a displayed the highest tumor selectivity, with SI values of 8.06 ± 0.03 and 9.26 ± 0.01 for A549 and HeLa cells, respectively (Table 3). Furthermore, cell viability profiles of all compounds in BEAS-2B cells were illustrated by dose–response curves obtained from MTT assays (Figure 9), confirming minimal cytotoxicity at concentrations of up to 100 μM. These data further support the selectivity and safety potential of the tested molecules.

The presence of fluorine atoms or fluorine-containing groups within the molecular structures contributed to their biological activity due to advantages in size, electronegativity, and metabolic stability. Specifically, fluorine substitution modulates lipophilicity, enhancing cellular absorption and transportation. Compounds **1a**, **1b**, and **1c**, bearing a single trifluoromethyl (CF_3_) group, maintained moderate lipophilicity facilitating efficient cellular uptake, whereas compound **1d**, having two CF_3_ groups, exhibited excessive hydrophobicity, adversely affecting solubility and bioavailability. Additionally, the introduction of an oxygen atom bridging the CF_3_ group to the phenyl ring in compound **1c** significantly enhanced cell viability and bioavailability, attributed to improved lipophilicity and absorption, compared to compound **1e** which lacked this bridging oxygen [80]. In conclusion, the tested substituted theophylline derivatives, particularly compound **1a**, showed potent anticancer activity coupled with a favorable selectivity profile, underscoring their therapeutic potential for cancer treatment. 

### 3.4. Apoptosis-Related Gene Expression Analysis by RT-qPCR

**Figure 10:**
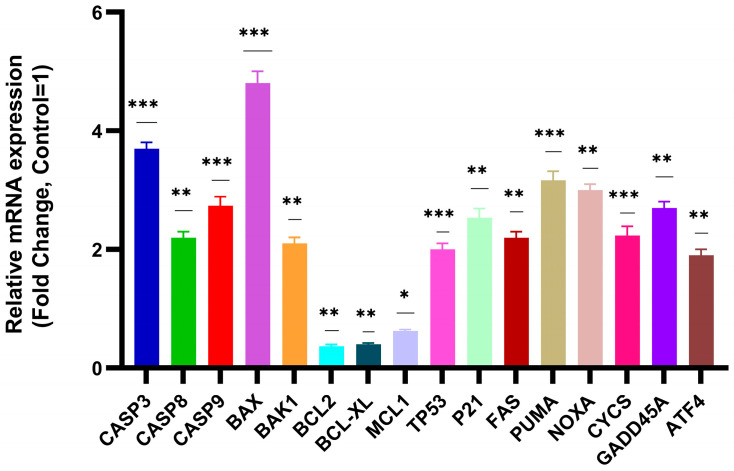
Quantitative RT-qPCR analysis of apoptosis-related genes in A549 cells treated with compound **1a** at IC_50_ concentrations for 48 h. Data are presented as fold change relative to untreated controls and expressed as mean ± SD (*n* = 3). Statistical significance was indicated as * *p* < 0.05, ** *p* < 0.01, and *** *p* < 0.001 compared to control.

**Figure 11:**
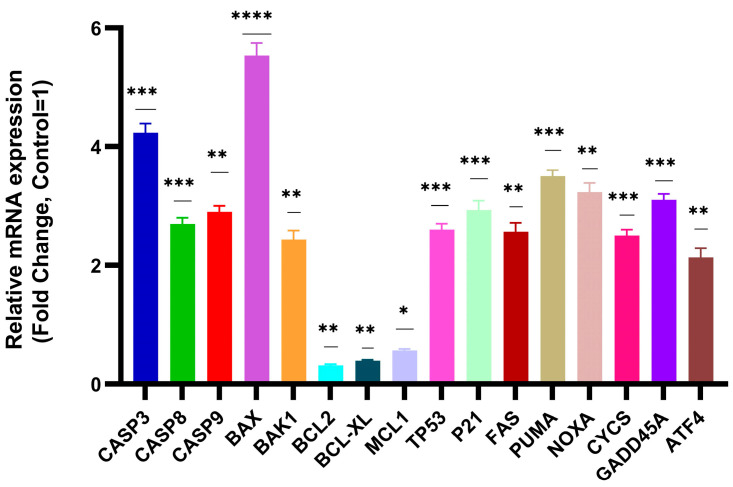
Quantitative RT-qPCR analysis of apoptosis-related genes in Hela cells treated with compound **1a** at IC_50_ concentrations for 48 h. Data are presented as fold change relative to untreated controls and expressed as mean ± SD (*n* = 3). Statistical significance was indicated as * *p* < 0.05, ** *p* < 0.01, *** *p* < 0.001, and **** *p* < 0.0001 compared to control.

To investigate the molecular mechanism underlying the anticancer activity of compound **1a**, quantitative RT-qPCR analyses were conducted to evaluate the expression levels of key apoptosis-related genes in A549 and HeLa cells. After treatment with compound **1a** at IC_50_ concentrations for 48 h, significant upregulation was observed in the expression levels of pro-apoptotic genes including CASP3, CASP8, CASP9, BAX, BAK1, PUMA, NOXA, and CYCS, as well as genes related to cell cycle and cellular stress responses such as TP53, CDKN1A (p21), FAS, GADD45A, and ATF4. Conversely, treatment with compound **1a** resulted in a notable downregulation of anti-apoptotic genes, specifically BCL2, BCL-XL, and MCL1 in both cell lines (Figure 10 and Figure 11). The marked induction of pro-apoptotic pathways and simultaneous suppression of anti-apoptotic signals strongly supports apoptosis as a primary mechanism for the observed anticancer activity of compound **1a**.

### 3.5. Protein-Level Characterization of Apoptotic Pathways

**Figure 12:**
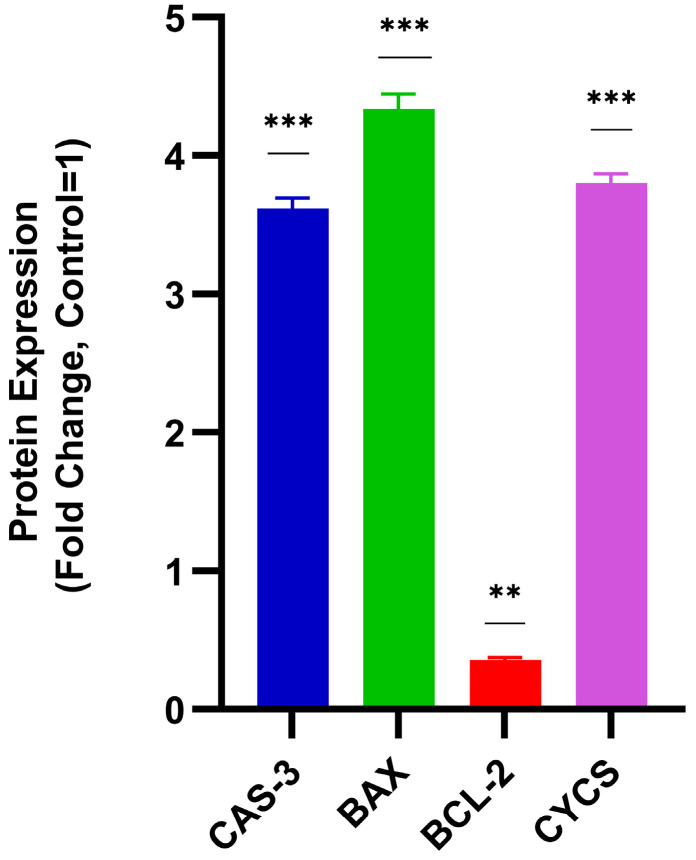
Protein expression in A549 cells treated with compound **1a** (IC_50_, 48 h), measured by ELISA. Data are shown as fold change vs. control (mean ± SD, *n* = 3; ** *p* < 0.01, *** *p* < 0.001).

**Figure 13:**
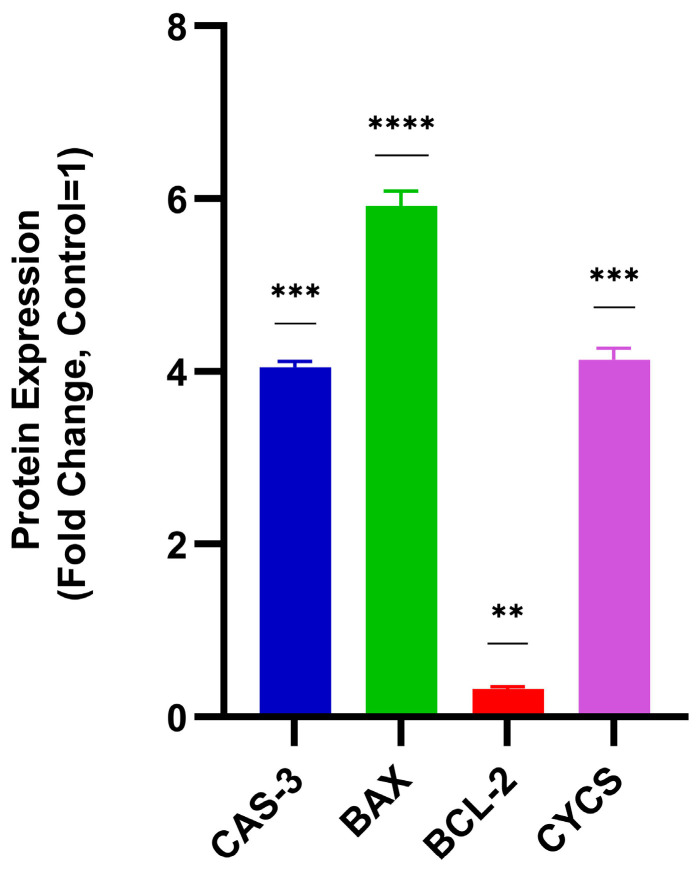
Protein expression in HeLa cells treated with compound **1a** (IC_50_, 48 h), measured by ELISA. Data are shown as fold change vs. control (mean ± SD, *n* = 3; ** *p* < 0.01, *** *p* < 0.001, and **** *p* < 0.0001).

To confirm the gene expression findings at the protein level, ELISA assays were performed to quantify the protein levels of Caspase-3, BAX, BCL-2, and Cytochrome C in A549 and HeLa cells following treatment with compound **1a** (IC_50_, 48 h). Consistent with RT-qPCR results, ELISA analyses revealed significant increases in the protein expression levels of pro-apoptotic markers Caspase-3, BAX, and Cytochrome C, coupled with a marked decrease in the anti-apoptotic protein BCL-2 in both cell lines compared to untreated controls (Figure 12 and Figure 13). These protein-level results further validate and reinforce the role of apoptosis induction as the key mode of action for compound **1a**.

### 3.6. Antimicrobial Activity

The antimicrobial activities of compounds on Gram-positive, Gram-negative bacteria, and yeasts were investigated by broth microdilution method. Antimicrobial activity of compounds **1a**–**1e** MIC values of *Staphylococcus aureus* (ATCC 29213), *Enterococcus faecalis* (ATCC 29212), *Escherichia coli* (ATCC 25922), *Pseudomonas aeruginosa* (ATCC 27853), and yeast *Candida albicans* (ATCC 10231) strains were determined as >1000 µg/mL. Antimicrobial activity of the 1% DMSO on *Staphylococcus aureus* (ATCC 29213), *Enterococcus faecalis* (ATCC 29212), *Escherichia coli* (ATCC 25922), *Pseudomonas aeruginosa* (ATCC 27853), and yeast *Candida albicans* (ATCC 10231) strains were not determined. No microorganism growth was detected in the negative control culture, no microbial growth was observed in the wells containing the positive control agents (ciprofloxacin for bacterial strains and fluconazole for *Candida albicans*), but all microorganisms grew in the growth control culture.

### 3.7. Antibiofilm Activity

The antibiofilm activities of the compounds **1a**–**1e** on Gram-positive, Gram-negative bacteria, and yeasts were investigated by the well plate assay. The antibiofilm activity of 1c was found to be 20.1% at a concentration of 1000 µg/mL on *Staphylococcus aureus*, 0.7–16% at a concentration of 0.488–500 µg/mL on *Enterococcus faecalis*, and 0.9–12.3% at a concentration of 0.488–1000 µg/mL on *Candida albicans*. Compound **1c** did not show antibiofilm activity on *Escherichia coli* and *Pseudomonas aeruginosa*. The antibiofilm activity of **1a** was found to be 1.08–14.7% at a concentration of 0.488–1000 µg/mL on *Enterococcus faecalis*, and 0.5–13.7% at a concentration of 0.244–1000 µg/mL on *Candida albicans*. Compound **1a** did not show antibiofilm activity on *Staphylococcus aureus*, *Escherichia coli,* and *Pseudomonas aeruginosa*. The antibiofilm activity of **1b** was found to be 5.7–23.1% at a concentration of 0.97–1000 µg/mL on *Enterococcus faecalis* and 0.8–23.9% at a concentration of 0.244–1000 µg/mL on *Candida albicans*. Compound **1b** did not show antibiofilm activity on *Staphylococcus aureus*, *Escherichia coli*, and *Pseudomonas aeruginosa*. The antibiofilm activity of **1d** was found to be 0.7–23.9% at a concentration of 0.244–1000 µg/mL on *Enterococcus faecalis* and 1.8–11.8% at a concentration of 0.244–1000 µg/mL on *Candida albicans*. Compound **1d** did not show antibiofilm activity on *Staphylococcus aureus*, *Escherichia coli*, and *Pseudomonas aeruginosa*. The antibiofilm activity of **1e** was found to be 13.6% at a concentration of 1000 µg/mL on *Staphylococcus aureus*, 5.1–13.9% at a concentration of 0.244–250 µg/mL on *Enterococcus faecalis*, and 0.4–23.4% at a concentration of 0.244–1000 µg/mL on *Candida albicans*. Compound **1e** did not show antibiofilm activity on *Escherichia coli* and *Pseudomonas aeruginosa*. The antibiofilm activity of DMSO was found to be 0.7–24.1% at a concentration of 0.00048–1% on *Enterococcus faecalis* and 2–16.4% at a concentration of 0.00048–1% on *Candida albicans*. The DMSO did not show antibiofilm activity on *Staphylococcus aureus*, *Escherichia coli* and *Pseudomonas aeruginosa*. The positive control agents, ciprofloxacin for bacterial strains and fluconazole for *Candida albicans*, demonstrated varying degrees of biofilm inhibition, with the highest activity observed against *Staphylococcus aureus* (50.56%), followed by *Escherichia coli* (36.67%), *Pseudomonas aeruginosa* (35.79%), *Candida albicans* (29.10%), and *Enterococcus faecalis* (18.11%). No microorganism growth and biofilm were detected in negative control cultures of Brucella broth containing 1% and 20% glucose, while microorganism growth and biofilm were detected in the positive control culture of Brucella broth containing 20% glucose. When evaluated in comparison with the biofilm inhibition levels of standard agents (ciprofloxacin and fluconazole), compounds **1b**, **1d**, and **1e** exhibited selective and in some cases promising antibiofilm activity, particularly against *Enterococcus faecalis* and *Candida albicans*. Notably, compound **1b** demonstrated up to 23.1% inhibition against *Enterococcus faecalis*, exceeding the level observed with ciprofloxacin (18.11%) for the same strain. Compound **1e** also showed considerable inhibition on *Candida albicans* (up to 23.4%), approaching the effect of fluconazole (29.10%). These findings suggest that certain theophylline derivatives possess meaningful antibiofilm potential, especially against Gram-positive bacteria and yeasts. These findings represent preliminary results demonstrating the potential anti-biofilm activity of certain theophylline derivatives. In our study, the antibiofilm activities of the molecules against *Staphylococcus aureus*, *Enterococcus faecalis*, and *Candida albicans* (all of which are major biofilm-producing pathogens commonly associated with hospital-acquired infections) appear promising. Given their strong biofilm-forming abilities, these microorganisms represent significant therapeutic challenges. Further studies focusing on synergistic combinations with conventional antimicrobials may also help improve their clinical relevance. The detailed results are summarized in Table 4.

In addition to the observed in vitro inhibition, the molecular docking results also support the potential of these compounds to interfere with biofilm-related regulatory pathways. Specifically, compound **1b** showed favorable interaction with the *Staphylococcus aureus* SarA protein, which plays a key role in biofilm regulation. It would be valuable in future studies to evaluate potential interactions with other biofilm-associated proteins, such as the enterococcal surface protein (Esp) in *Enterococcus faecalis* and the Als protein family in *Candida albicans*, to better understand the mechanistic basis of the observed effects.

## 4. Conclusions

In conclusion, five novel theophylline derivatives bearing CF_3_/OCF_3_ substituents at the N(7) position were successfully synthesized and comprehensively characterized using various spectroscopic techniques, including ^1^H NMR, ^13^C NMR, ^19^F NMR, FT-IR, and elemental analysis. The biological activities of these molecules were investigated through a combination of computational and experimental approaches.

Experimental validation via in vitro studies further substantiated the anticancer potential of these derivatives, with compounds **1a**, **1b**, and **1c** showing substantial cytotoxic effects against HeLa cells as demonstrated by the remarkable IC_50_ values. Importantly, cytotoxicity assessments in non-tumorigenic bronchial epithelial cells (BEAS-2B) revealed high selectivity indices, particularly for compound **1a**, signifying a favorable therapeutic index and minimal toxicity toward normal cells. Such tumor selectivity is critical in cancer treatment, where the challenge lies in effectively targeting cancer cells while sparing normal tissue [81].

Detailed mechanistic studies employing RT-qPCR and ELISA provided compelling evidence that apoptosis induction is the fundamental mechanism underlying the anticancer activity observed with compound **1a**. The pronounced upregulation of key pro-apoptotic markers (CASP3, CASP8, CASP9, BAX, PUMA, and NOXA) alongside significant suppression of anti-apoptotic proteins (BCL-2, BCL-XL, MCL1) confirms the activation of intrinsic and extrinsic apoptotic pathways [82]. Additionally, the notable increase in the expression of cell cycle regulatory and stress-related genes such as TP53, CDKN1A (p21), GADD45A, and ATF4 further highlights compound **1a**’s capability to modulate multiple cellular processes involved in cancer progression and survival [83].

Antimicrobial activity assessments indicated limited antibacterial efficacy, with MIC values generally exceeding 1000 µg/mL, suggesting that the primary biomedical relevance of these derivatives lies in their anticancer properties. However, modest antibiofilm activities observed warrant further optimization and exploration of structure–activity relationships to enhance efficacy.

Molecular docking studies provided critical insights into their interaction potential with pivotal biological targets such as VEGFR-2, Cytochrome P450, estrogen receptor, DNA Gyrase, and SarA. Specifically, compound **1b** exhibited exceptional affinity towards VEGFR-2, estrogen receptor, and SarA according to the binding affinity values. On the other hand, compound **1e** showed strong interactions with Human Cytochrome P450, indicating potential modulation of metabolic pathways, while compound **1a** displayed significant binding affinity towards DNA Gyrase, highlighting possible antibacterial applications.

In future research, exploring additional biological targets and diverse substituents on the theophylline framework could substantially enhance anticancer potency, selectivity, and overall therapeutic efficacy. These findings underscore the remarkable potential of substituted theophylline derivatives, especially compound **1a**, as promising selective anticancer candidates, paving the way for innovative cancer treatment strategies and clinical translation.

## Figures and Tables

**Table 1:** Some selected physical and spectroscopic properties of the N(7)-substituted theophyllines **1a**–**1e**.

Compounds	Molecular Formula	Yield (%)	M.p. (°C)	IR (*v*_C(2)=O,_ *v*_C(6)=O_) (cm^−1^)	IR (*v*_C(8)=N_) (cm^−1^)	^1^H NMR (C(8)-*H*) (ppm)	^13^C NMR (C(8)) (ppm)
**1a**	C_15_H_13_F_3_N_4_O_2_	92	214–215	1704, 1650	1550	7.53	141.50
**1b**	C_15_H_13_F_3_N_4_O_2_	81	128–129	1694, 1644	1547	7.58	139.78
**1c**	C_15_H_13_F_3_N_4_O_2_	87	196–197	1703, 1654	1548	7.56	140.88
**1d**	C_16_H_12_F_6_N_4_O_2_	93	157–158	1703, 1645	1551	7.69	140.71
**1e**	C_15_H_13_F_3_N_4_O_3_	90	177–178	1703, 1656	1547	7.54	140.74

**Table 2:** Interaction details of the molecules **1a**–**1e** against VEGFR-2, Human Cytochrome P450, Estrogen Receptor, DNA Gyrase, and SarA.

Molecules	BA *	Amino Acids Residue
		**VEGFR-2**
**1a**	−5.07	Glu883, His1024 (H-bonds), Asp1044 (halogenic interaction), Ile886, Leu887, Ile890, Val896, Leu1017 (alkylic interactions), Lys866, Val897, Cys1022, Ile1042, Cys1043 (van der Waals interactions)
**1b**	−5.69	Lys866, His1024, Ile1042 (H-bonds), Glu883, Asp1044 (halohenic interactions), Ile886 (pi-interactions), Ile890, Val896, Val897, Leu1017 (alkylic interacttions), Phe843, Leu887, Cys1022, Cys1043 (van der Waals interactions)
**1c**	−5.27	Ile1023, His1024, Ile1042 (H-bonds), Glu883, Asp1044 (pi-interactionss), val896, Leu1017 (alkylic interactions), Phe843, Lys866, Ile886, Ile890, Val897, Cys1022, Cys1043 (van der Waals interactions)
**1d**	−5.17	Lys866, His1024, Ile1042 (H-bonds), Glu883, Asp1044 (halogenic interactions), Leu887 (pi-interactions), Ile886, Ile890, Val896, Leu1017 (alkylic interactions), Ser882, Phe843, Val897, Cys1022, Ile1023, Cys1043 (van der Waals interactions)
**1e**	−5.34	Lys866, His1024 (H-bonds), Glu883 (halogenic interaction), Leu887, Asp1044 (pi-interactions), Ile886, Ile890, Val896, Leu1017, Ile1042 (alkylic interactions) Phe843, Ala879, Val897, Cys1022, Ile1023, Cys1043 (van der Waals interactions)
**AAFP**	−5.61	Glu883, Glu915, Cys917, Asp1044 (H-bonds), Val846 (pi-interaction), Ile1042, Cys1043 (halogenic interactions), Leu838, Ala864, Leu887, Ile890, Val896, Val897, Val914, Leu1017, Leu1033 (alkylic interactions), Gly839, Arg840, Gly841, Lys866, Ile886, Phe916, His1024 (van der Waals interactions)
		**Human Cytochrome P450**
**1a**	−6.37	Ala367, Cys442 (H-bonds), Arg440 (halogenic interaction), Val366 (pi-interaction), Leu361, leu370, Ile371, Phe435, Ala448 (alkylic interactions), Arg96, Thr306, Val310, Gly436, Ser441 (van der Waals interactions)
**1b**	−6.42	Gly436, Arg440, Ser441, Cys442, Ile443 (H-bonds), His373 (halogenic interactions), Ala302, Ala367, Leu370 (alkylic interactions), Leu86, Arg96, Ile112, Arg125, Thr306, Ile371, Pro434, Phe435, Ala437, Gly444 (van der Waals interactions)
**1c**	−6.60	Gly436, Ser441, Cys442 (H-bonds), Phe435, Arg440 (halogenic interactions), Thr306, Val366 (pi-interactions), Ala302, Ala367, Leu370, Ala448 (alkylic interactions), Arg96, Pro434, Ala437 (van der Waals interactions)
**1d**	−6.28	Gly436, Ser441, Cys442, Ile443 (H-bonds), Phe435, Arg440 (halogenic interactions), Val366 (pi-interaction), Leu361, Ala367, Leu370, Ala448 (alkylic interactions), Arg96, Ala113, Arg125, Thr306, Ile371, Ala437, Gly444 (van der Waals interactions)
**1e**	−6.69	Pro434, Gly436, Ala437, Ser441, Cys442 (H-bonds), His373, Arg440 (halogenic interactions), Thr306, Val366 (pi-interactions), Leu361, Ala367, Leu370, Phe435, Ala448 (alkylic interactions), Leu86, Arg96, Val310, Ile371, Leu452 (van der Waals interactions)
**Abiraterone**	−9.01	Asn202 (H-bond), Ala113, Phe114, Ile205, Leu209, Ala302, Val482 (alkylic interactions), Tyr201, Ile206, Arg239, Gly297, Asp298, Gly301, Glu305, Thr306, Val366, Ala367, Ile371, Cys442, Val483 (van der Waals interactions)
		**Estrogen Receptor**
**1a**	−6.64	Leu346, Glu353 (H-bonds), Leu384, Met388 (pi interactions), Met343, Leu349, Ala350, Leu387, Leu391, Met421, Leu525 (alkylic interactions), Thr347, Arg394, Phe404, Glu419, Gly420, Ile424, Gly51-21, His524 (van der Waals interactions)
**1b**	−6.70	Glu353, Lys449 (H-bonds), Glu323, Pro324 (halogenic interactions), Ile326 (pi-interaction), Leu327, His356, Met357, Trp360, Ile386, Arg394 (alkylic interactions), Pro325, Gly390, Trp393, Phe445 (van der Waals interactions)
**1c**	−6.36	Leu387, Arg394, Gly521, His524, Leu525 (H-bonds), Glu419, Gly420 (halogenic interactions), Ala350, Glu353 (pi-interactions), Leu346, Leu349, Leu384, Met388, Leu391, Phe404 (alkylic interactions), Met343, Thr347, Trp383, Met421, Ile424 (van der Waals interactions)
**1d**	−6.40	Pro325, Ile326, Ile386, Trp393, Lys449 (H-bonds), Glu323, Pro324 (halogenic interactions), Glu353, Arg394 (pi-interactions), Leu327, Phe445 (alkylic interactions), Met357, Leu387, Gly390, Val446 (van der Waals interactions)
**1e**	−6.54	Pro325, Glu353, Lys449 (H-bonds), Trp393, Phe445 (pi-interactions), Pro324, Ile326, Leu327, His356, Met357, Trp360, Ile386, Arg394 (alkylic interactions), Glu323, Gly390 (van der Waals interactions)
**Hat**	−10.35	Asp351, Glu353, Arg394 (H-bonds), Met343, Leu346 (pi-interactions), Ala350, Leu387, Met388, Leu391, Ile424, Leu428, Leu525 (alkylic interactions), Thr347, Leu349, Trp383, Leu384, Phe404, Glu419, Gly420, Gly521, His524 (van der Waals interactions)
		**DNA Gyrase**
**1a**	−5.56	Asn46, Val71, Asp73, Thr165 (H-bonds), Val43, Ala47, Ile78, Ile90, Val120, Val167 (alkylic interactions), Asp49, Glu50, Gln72, Met91 (van der Waals interactions)
**1b**	−5.06	Asn46, Asp (H-bonds), Val43, Val71, Asp73, Thr165 (halogenic interactions), Ala47, Ile90, Val120, Val167 (alkylic interactions), Val44, Glu50, Gln72, Ile78, Met91, Met166 (van der Waals interactions)
**1c**	−4.62	Asn46, Asp49, Val167 (H-bonds), Val43, Val71, Thr165 (halogenic interactions), Ala47, Ile78, Ile90 (alkylic interactions), Glu50, Gln72, Asp73, Val120, Met166 (van der Waals interactions)
**1d**	−4.59	Asn46, Asp49 (H-bonds), Val43, Val71, Asp73, Thr165 (halogenic interactions), Ala47, Ile78, Ile90, Met91, Val120 (alkylic interactions), Val44, Glu50, Ile59, Gln72, Leu132, Met166 (van der Waals interactions)
**1e**	−5.11	Asn46, Asp49, Val167 (H-bonds), Val71, Thr165 (halogenic interactions), Ala47, Ile78, Ile90 (alkylic interactions),Val43, Glu50, Gln72, Asp73, Val120, Met166 (van der Waals interactions)
**Clorobiocin**	−7.15	Asn46, Asp73, Arg136 (H-bonds), Glu50, Arg76, Thr165 (pi-interactions), Gly77 (halogenic interaction), Ala47, Ile78, Pro79, Ile90 (alkylic interactions), Val43, Asp49, Val71, Gln72, Ala86, Met91, Val120, Met166, Val167 (van der Waals interactions)
		**SarA**
**1a**	−4.56	His 159 (H-bond), Leu160 (pi-interaction), Phe134, Phe137, Ala138 (alkylic interactions), Thr141, Tyr142, Glu145, Asn161, Tyr162 (van der Waals interactions)
**1b**	−4.90	Ala138, Tyr162 (H-bonds), His159 (halogenic interaction), Phe134, Tyr142 (pi-interactions), Phe137, Leu160 (alkylic interactions), Thr141, Glu145 (van der Waals interactions)
**1c**	−4.68	Ala138, Tyr162 (H-bonds), Glu145 (halogenic interaction), Phe134, Tyr142 (pi-interactions), Leu160 (alkylic interaction), Phe137, Thr141, His159 (van der Waals interactions)
**1d**	−4.64	Ala138, Tyr162 (H-bonds), Leu122 (halogenic interaction), Phe137 (pi-interaction), Lys123, Ile126, Phe134, Leu160, Ile215 (alkylic interactions), Thr141, Tyr142 (van der Waals interactions)
**1e**	−4.53	Ala138, Tyr162 (H-bonds), Glu145 (halogenic interaction), Phe134, Tyr142 (pi-interactions), Phe137, Leu160 (alkylic interactions), Thr141, Asn146, His159 (van der Waals interactions)

* BA: Binding Affinity in kcal/mol.

**Table 3:** IC_50_ values (μM) and selectivity index (SI) of substituted theophylline derivatives in BEAS-2B, A549, and HeLa cell lines. SI values were calculated as the ratio of IC_50_ in BEAS-2B cells to IC_50_ in cancer cells. Data are expressed as mean ± SD (*n* = 3).

Molecules	BEAS-2B (μM)	A549 (μM)	SI (A549)	HeLa (μM)	SI (HeLa)
**1a**	100 ± 2	12.4 ± 0.7	8.06 ± 0.10	11.5 ± 1.2	8.70 ± 0.20
**1b**	120 ± 3	13.6 ± 0.9	8.82 ± 0.20	14.7 ± 0.7	8.16 ± 0.16
**1c**	90 ± 3	15.7 ± 0.6	5.73 ± 0.15	11.5 ± 1.2	7.83 ± 0.22
**1d**	150 ± 4	31.4 ± 1.0	4.78 ± 0.14	34.5 ± 1.0	4.35 ± 0.13
**1e**	140 ± 3	29.1 ± 0.5	4.81 ± 0.09	32.4 ± 0.8	4.32 ± 0.11

**Table 4:** In vitro antibiofilm activity (%) of compounds **1a**–**1e**.

Compound	Concentration Range (µg/mL or %)	*Staphylococcus aureus* (%)	*Enterococcus* *faecalis* (%)	*Escherichia coli* (%)	*Pseudomonas* *aeruginosa* (%)	*Candida* *albicans* (%)
**1a**	0.488–1000	–	1.08–14.7	–	–	0.5–13.7
**1b**	0.97–1000	–	5.7–23.1	–	–	0.8–23.9
**1c**	0.488–1000	20.1	0.7–16.0	–	–	0.9–12.3
**1d**	0.244–1000	–	0.7–23.9	–	–	1.8–11.8
**1e**	0.244–1000	13.6	5.1–13.9	–	–	0.4–23.4
Brucella broth (NC)	–	–	–	–	–	–
Ciprofloxacin (PC)	0.03125–64	50.56	18.11	36.67	35.79	NT
Fluconazole (PC)	0.3125–640	NT	NT	NT	NT	29.10

Note: (–) indicates no inhibition detected.

## Data Availability

The datasets generated and/or analyzed during the current study are available from the corresponding author.

## Supplementary Materials

The following supporting information can be downloaded at https://www.mdpi.com/article/10.3390/biology14091180/s1, Figure S1. ^19^F NMR spectrum of **1a** (CDCl_3_, 25 °C, TMS, 376 MHz); Figure S2. ^1^H NMR spectrum of **1a** (CDCl_3_, 25 °C, TMS, 400 MHz); Figure S3. ^13^C NMR spectrum of **1a** (CDCl_3_, 25 °C, TMS, 101 MHz); Figure S4. FT-IR spectrum of **1a**; Figure S5. ^19^F NMR spectrum of **1b** (CDCl_3_, 25 °C, TMS, 376 MHz); Figure S6. ^1^H NMR spectrum of **1b** (CDCl_3_, 25 °C, TMS, 400 MHz); Figure S7. ^13^C NMR spectrum of **1b** (CDCl_3_, 25 °C, TMS, 101 MHz); Figure S8. FT-IR spectrum of **1b**; Figure S9. ^19^F NMR spectrum of **1c** (CDCl_3_, 25 °C, TMS, 376 MHz); Figure S10. ^1^H NMR spectrum of **1c** (CDCl_3_, 25 °C, TMS, 400 MHz); Figure S11. ^13^C NMR spectrum of **1c** (CDCl_3_, 25 °C, TMS, 101 MHz); Figure S12. FT-IR spectrum of **1c**; Figure S13. ^19^F NMR spectrum of **1d** (CDCl_3_, 25 °C, TMS, 376 MHz); Figure S14. ^1^H NMR spectrum of **1d** (CDCl_3_, 25 °C, TMS, 400 MHz); Figure S15. ^13^C NMR spectrum of **1d** (CDCl_3_, 25 °C, TMS, 101 MHz); Figure S16. FT-IR spectrum of **1d**; Figure S17. ^19^F NMR spectrum of **1e** (CDCl_3_, 25 °C, TMS, 376 MHz); Figure S18. ^1^H NMR spectrum of **1e** (CDCl_3_, 25 °C, TMS, 400 MHz); Figure S19. ^13^C NMR spectrum of **1e** (CDCl_3_, 25 °C, TMS, 101 MHz); Figure S20. FT-IR spectrum of **1e**; Figure S21. Interaction Residue and Details of **1a** against VEGFR2; Figure S22. The overlaps of the molecular docking poses of **1a** against VEGFR2; Figure S23. The overlaps of the molecular docking poses of **1b** against VEGFR2; Figure S24. Interaction Residue and Details of 1c against VEGFR2; Figure S25. The overlaps of the molecular docking poses of **1c** against VEGFR2; Figure S26. Interaction Residue and Details of **1d** against VEGFR2; Figure S27. The overlaps of the molecular docking poses of **1d** against VEGFR2; Figure S28. Interaction Residue and Details of **1e** against VEGFR2; Figure S29. The overlaps of the molecular docking poses of **1e** against VEGFR2; Figure S30. Interaction Residue and Details of **1a** against Human Cytochrome P450; Figure S31. The overlaps of the molecular docking poses of **1a** against Human Cytochrome P450; Figure S32. Interaction Residue and Details of **1b** against Human Cytochrome P450; Figure S33. The overlaps of the molecular docking poses of **1b** against Human Cytochrome P450; Figure S34. Interaction Residue and Details of **1c** against Human Cytochrome P450; Figure S35. The overlaps of the molecular docking poses of **1c** against Human Cytochrome P450; Figure S36. Interaction Residue and Details of **1d** against Human Cytochrome P450; Figure S37. The overlaps of the molecular docking poses of **1d** against Human Cytochrome P450; Figure S38. The overlaps of the molecular docking poses of **1e** against Human Cytochrome P450; Figure S39. Interaction Residue and Details of **1a** against estrogen receptor; Figure S40. The overlaps of the molecular docking poses of **1a** against estrogen receptor; Figure S41. The overlaps of the molecular docking poses of **1b** against estrogen receptor; Figure S42. Interaction Residue and Details of **1c** against estrogen receptor; Figure S43. The overlaps of the molecular docking poses of **1c** against estrogen receptor; Figure S44. Interaction Residue and Details of **1d** against estrogen receptor; Figure S45. The overlaps of the molecular docking poses of **1d** against estrogen receptor; Figure S46. Interaction Residue and Details of **1e** against estrogen receptor; Figure S47. The overlaps of the molecular docking poses of **1e** against estrogen receptor; Figure S48. The overlaps of the molecular docking poses of **1a** against DNA Gyrase; Figure S49. Interaction Residue and Details of **1b** against DNA Gyrase; Figure S50. The overlaps of the molecular docking poses of **1b** against DNA Gyrase; Figure S51. Interaction Residue and Details of **1c** against DNA Gyrase; Figure S52. The overlaps of the molecular docking poses of **1c** against DNA Gyrase; Figure S53. Interaction Residue and Details of **1d** against DNA Gyrase; Figure S54. The overlaps of the molecular docking poses of **1d** against DNA Gyrase; Figure S55. Interaction Residue and Details of **1e** against DNA Gyrase; Figure S56. The overlaps of the molecular docking poses of **1e** against DNA Gyrase; Figure S57. Interaction Residue and Details of **1a** against SarA; Figure S58. The overlaps of the molecular docking poses of **1a** against SarA; Figure S59. The overlaps of the molecular docking poses of **1b** against SarA; Figure S60. Interaction Residue and Details of **1c** against SarA; Figure S61. The overlaps of the molecular docking poses of **1c** against SarA; Figure S62. Interaction Residue and Details of **1d** against SarA; Figure S63. The overlaps of the molecular docking poses of **1d** against SarA; Figure S64. Interaction Residue and Details of **1e** against SarA; Figure S65. The overlaps of the molecular docking poses of **1e** against SarA. Figure S66. Primer Sequences Used in Quantitative RT-PCR Analysis.
