# Supplementary material for: Synthesis, Characterization, and Screening Anticancer—Antibiofilm Activities of Theophylline Derivatives Containing CF3/OCF3 Moiety
Source: Biology (Basel). 2025 Sep 2;14(9):1180. doi: 10.3390/biology14091180 (PMC12467740; doi:10.3390/biology14091180)
Supplement: Supplementary file 1 [file biology-14-01180-s001.zip › biology-3773137-supplementary.pdf]

Synthesis, Characterization, Biological Activities and Molecular Docking of Theophylline Derivatives Containing CF_3/OCF_3 Moiety

Serpil Demir Düşünceli[1*], Kübra Açıkalın Coşkun[2], Murat Kaloğlu[1], Elvan Üstün[3], Reyhan Çalışkan[4], Yusuf Tutar[5*]

[1] *İnönü University, Faculty of Science and Arts, Department of Chemistry, 44280 Malatya, Türkiye*

[2] *İstanbul Aydın University, Faculty of Medicine, Department of Basic Medical Sciences, Division of Biology, İstanbul, Türkiye*

[3] *Ordu University, Faculty of Science and Art, Department of Chemistry, Ordu, Türkiye*

[4] *Samsun University, Faculty of Medicine, Department of medical microbiology, Samsun, Türkiye*

[5] *Recep Tayyip Erdoğan University, Faculty of Medicine, Department of Basic Medical Sciences, Division of Biochemistry, Rize, Türkiye*

Supporting Information

(Table of Contents)

**Contents**	**Pages**
Characterisation data of 1,3-Dimethyl-7-(2-(trifluoromethyl)benzyl)-3,7-dihydro-1*H*-purine-2,6-dione, **1a**	S1-S2
Characterisation data of 1,3-Dimethyl-7-(3-(trifluoromethyl)benzyl)-3,7-dihydro-1*H*-purine-2,6-dione, **1b**	S3-S4
Characterisation data of 1,3-Dimethyl-7-(4-(trifluoromethyl)benzyl)-3,7-dihydro-1*H*-purine-2,6-dione, **1c**	S5-S6
Characterisation data of 1,3-Dimethyl-7-(3,5-bis(trifluoromethyl)benzyl)-3,7-dihydro-1*H*-purine-2,6-dione, **1d**	S7-S8
Characterisation data of 1,3-Dimethyl-7-(4-(trifluoromethoxy)benzyl)-3,7-dihydro-1*H*-purine-2,6-dione, **1e**	S9-S10
Interaction Residue and Details, and The overlaps of the molecular docking poses of **1a-1e** against VEGFR2	S11-S15
Interaction Residue and Details, and The overlaps of the molecular docking poses of **1a-1e** against Human Cytochrome P450.	S15-S19
Interaction Residue and Details, and The overlaps of the molecular docking poses of **1a-1e** against estrogen receptor	S20-S24
Interaction Residue and Details, and The overlaps of the molecular docking poses of **1a-1e** against DNA Gyrase	S24-S28
Interaction Residue and Details, and The overlaps of the molecular docking poses of **1a-1e** against SarA	S29-S33
Primer Sequences Used in Quantitative RT-PCR Analysis	S-34

1,3-Dimethyl-7-(2-(trifluoromethyl)benzyl)-3,7-dihydro-1*H*-purine-2,6-dione (1a)

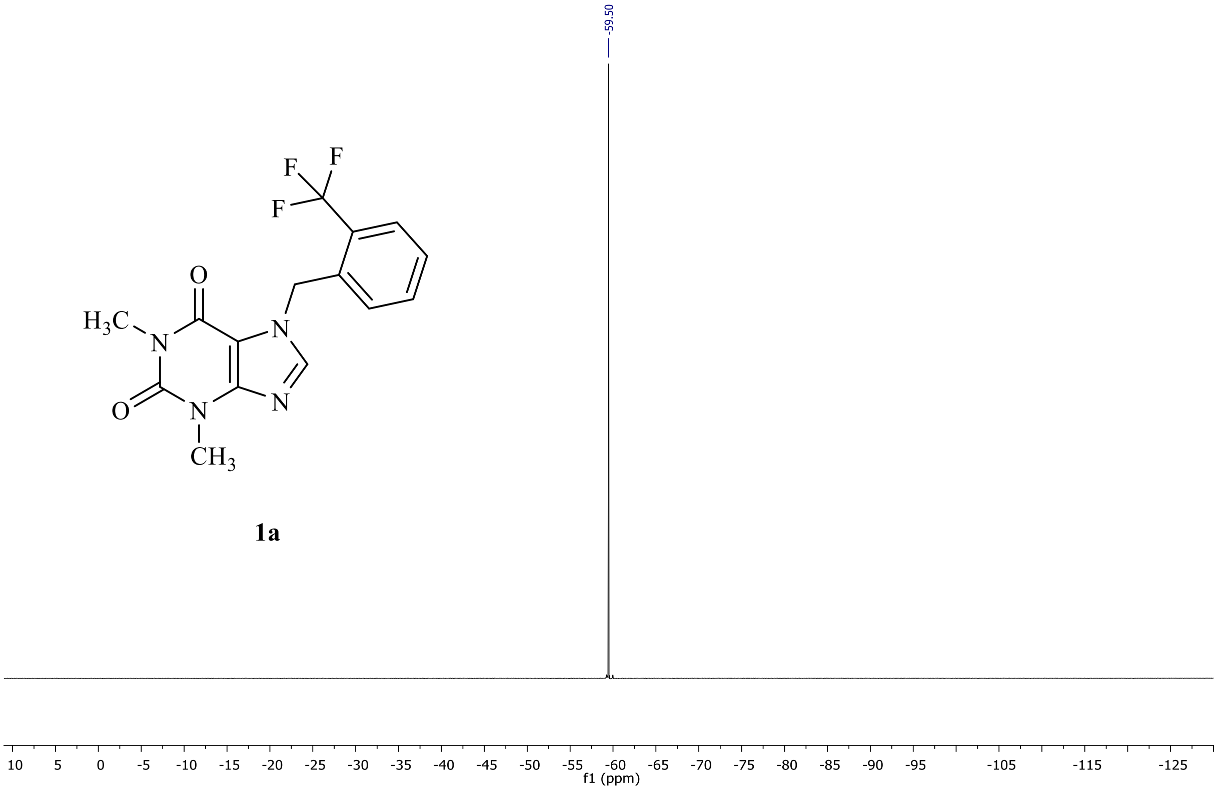

Figure S1. ^{19}F NMR spectrum of **1a** ($CDCl_3$, 25 °C, TMS, 376 MHz).

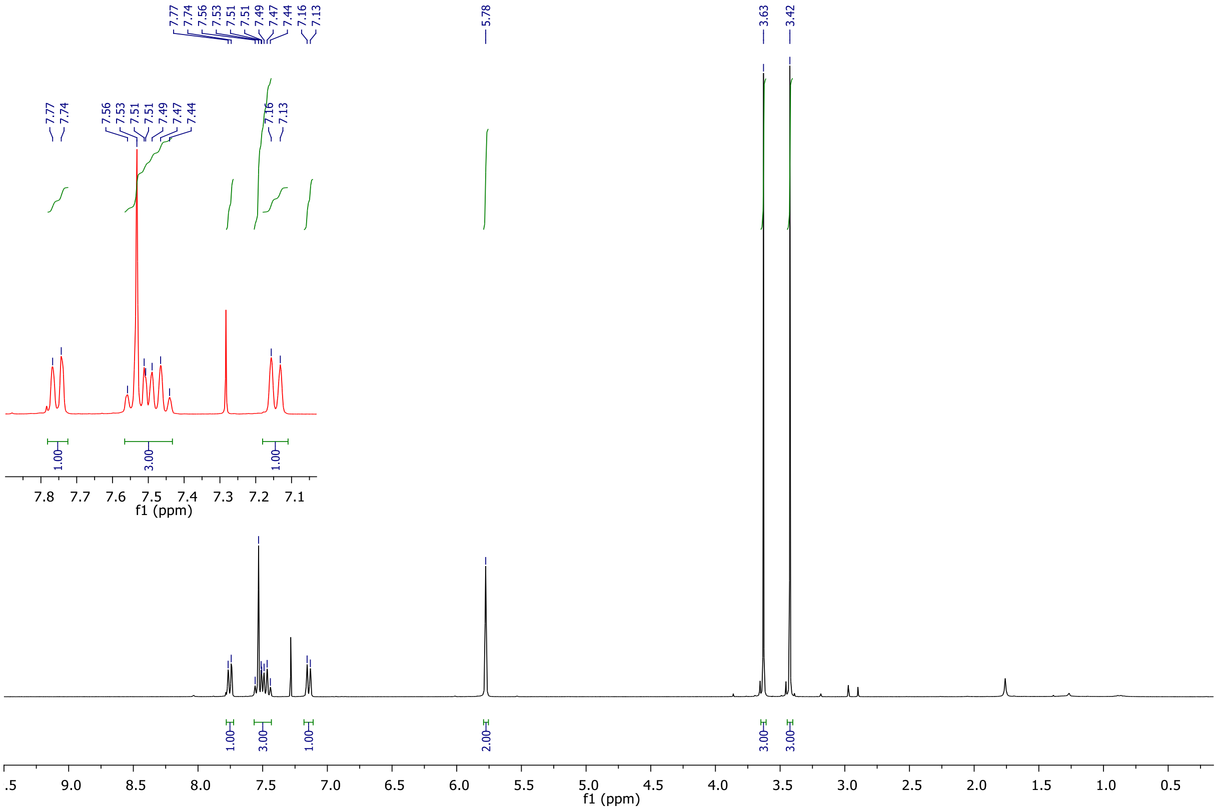

Figure S2. ^{1}H NMR spectrum of **1a** ($CDCl_3$, 25 °C, TMS, 400 MHz).

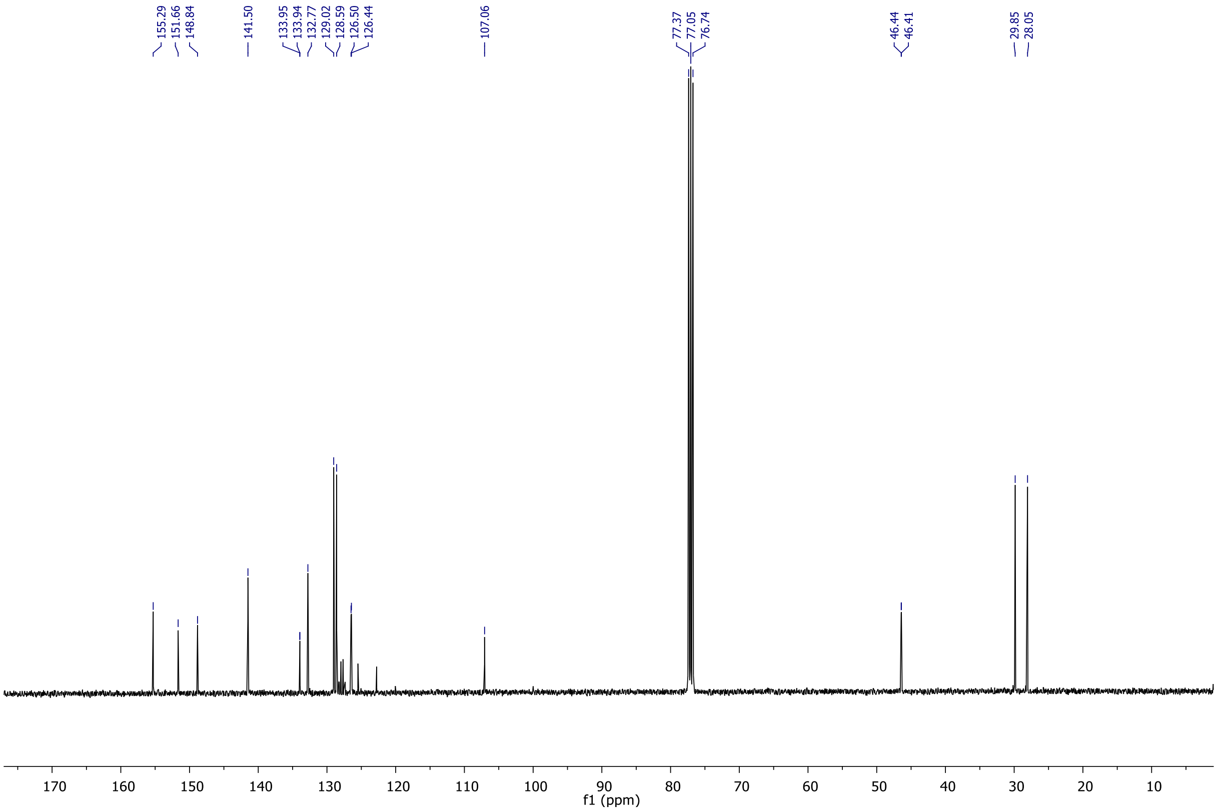

Figure S3. ^{13}C NMR spectrum of **1a** ($CDCl_3$, 25 °C, TMS, 101 MHz).

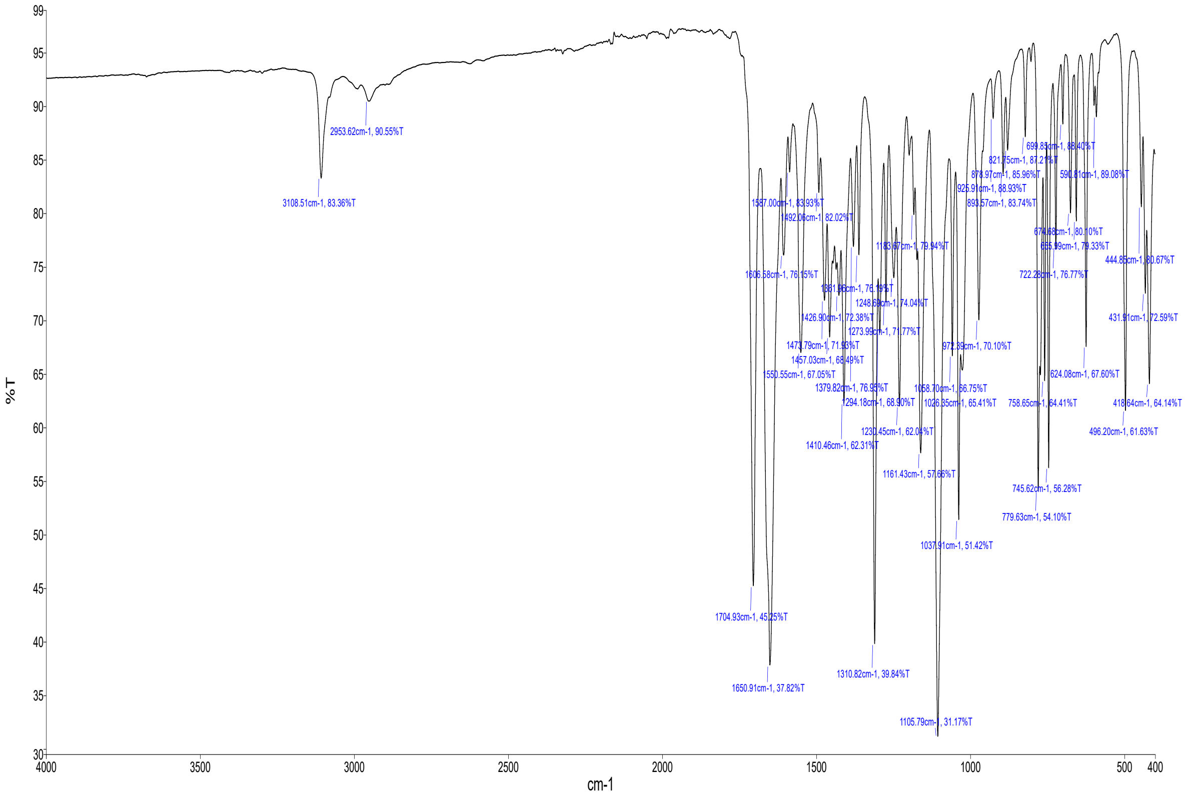

Figure S4. FT-IR spectrum of **1a**.

1,3-Dimethyl-7-(3-(trifluoromethyl)benzyl)-3,7-dihydro-1*H*-purine-2,6-dione (1b)

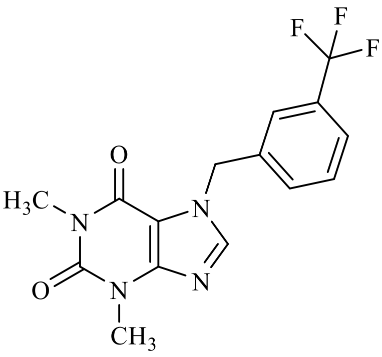

1b

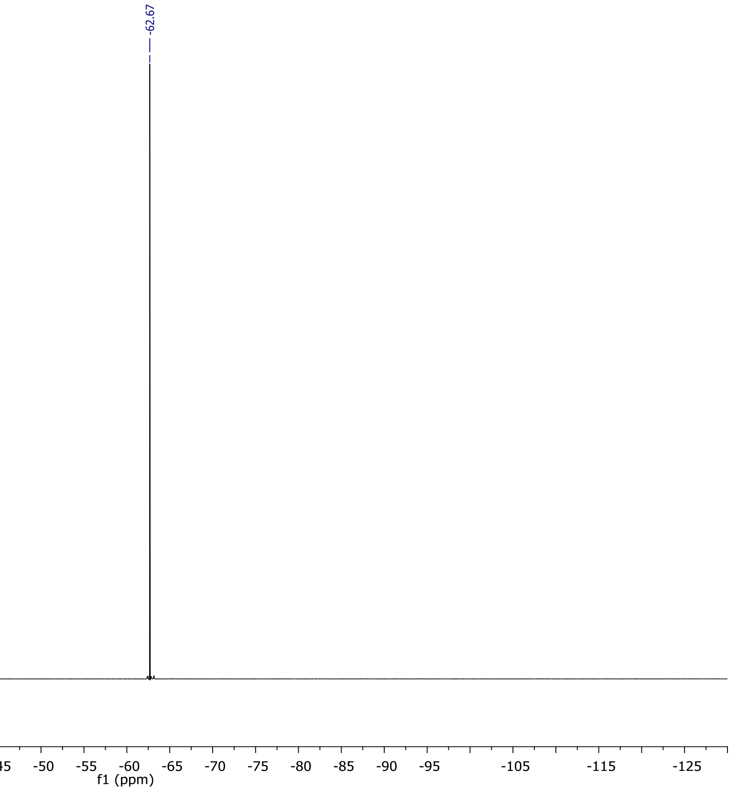

Figure S5. ^{19}F NMR spectrum of **1b** ($CDCl_3$, 25 °C, TMS, 376 MHz).

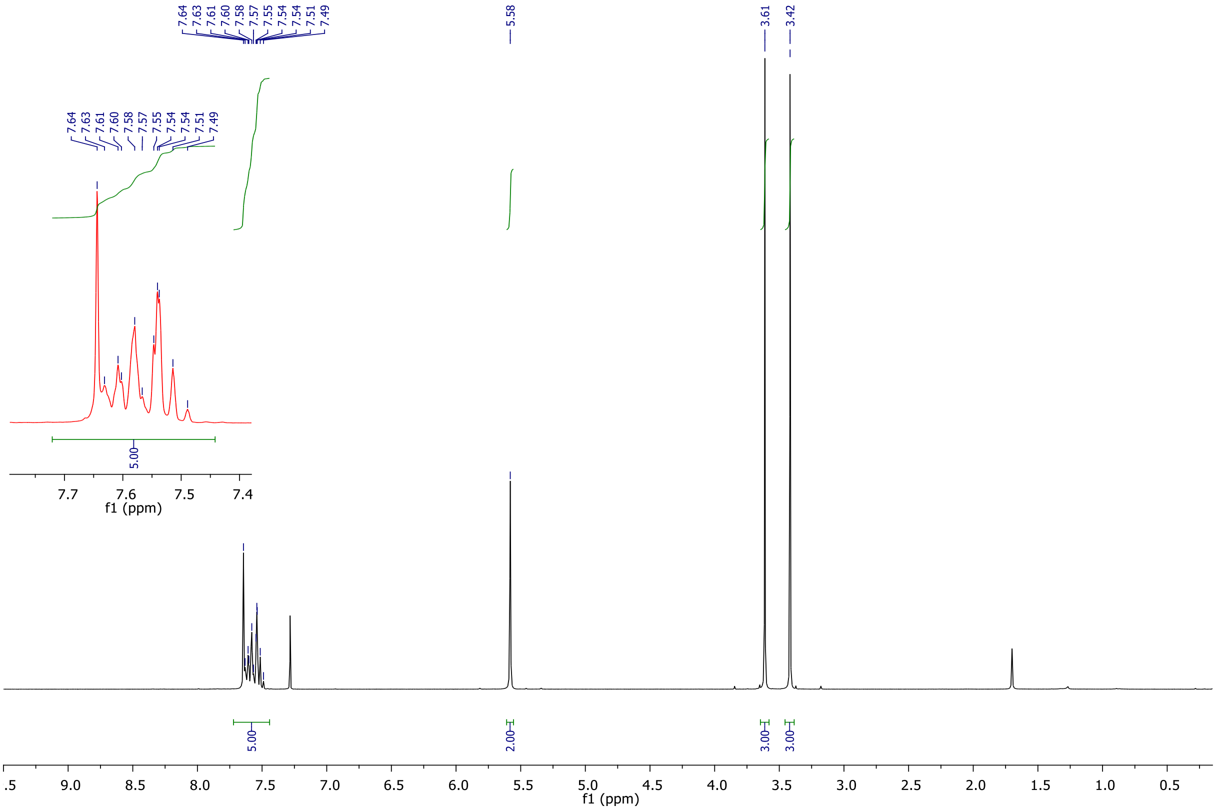

Figure S6. ^{1}H NMR spectrum of **1b** ($CDCl_3$, 25 °C, TMS, 400 MHz).

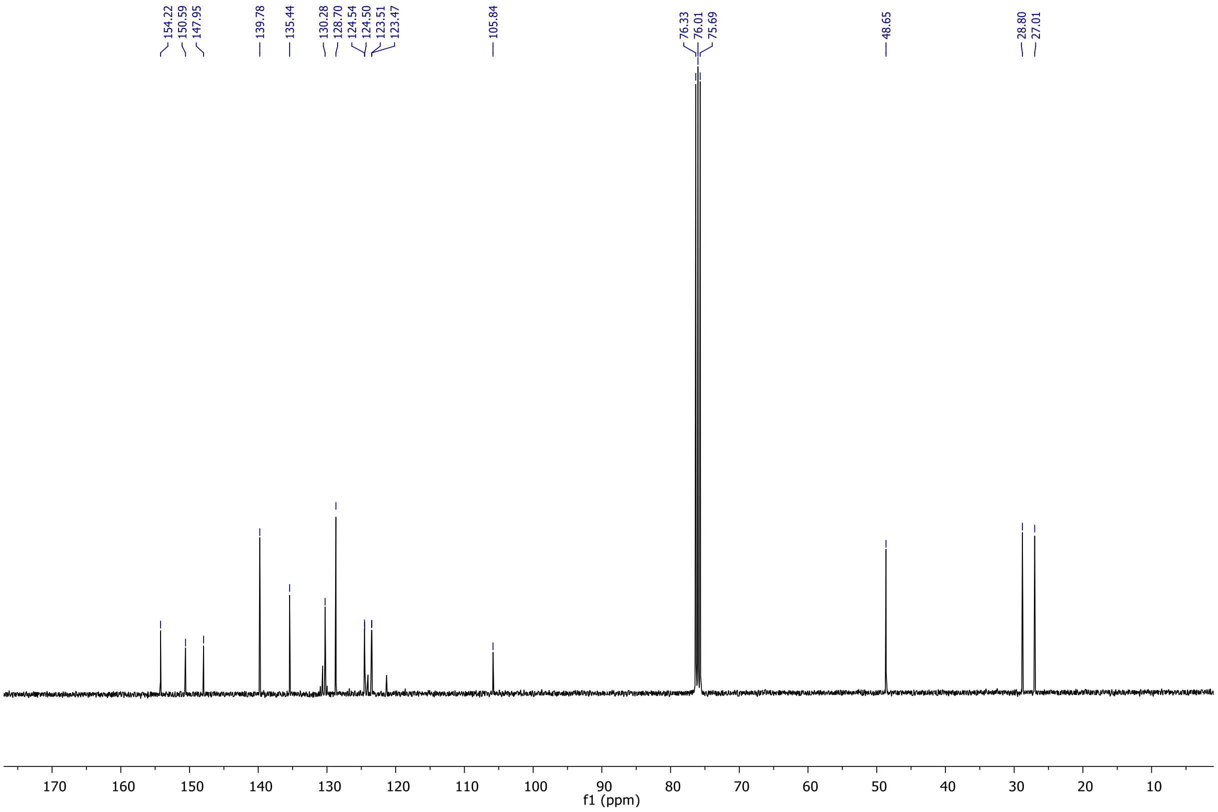

Figure S7. ^{13}C NMR spectrum of **1b** ($CDCl_3$, 25 °C, TMS, 101 MHz).

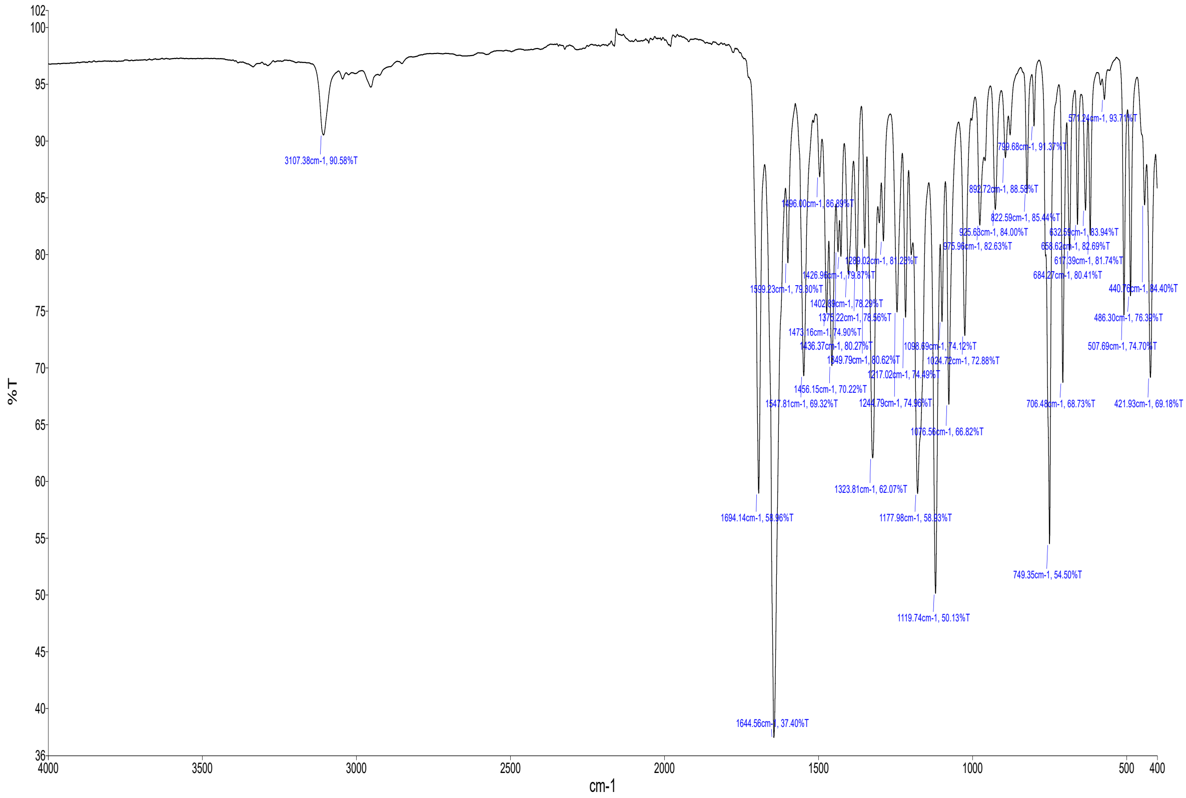

Figure S8. FT-IR spectrum of **1b**.

1,3-Dimethyl-7-(4-(trifluoromethyl)benzyl)-3,7-dihydro-1*H*-purine-2,6-dione (1c)

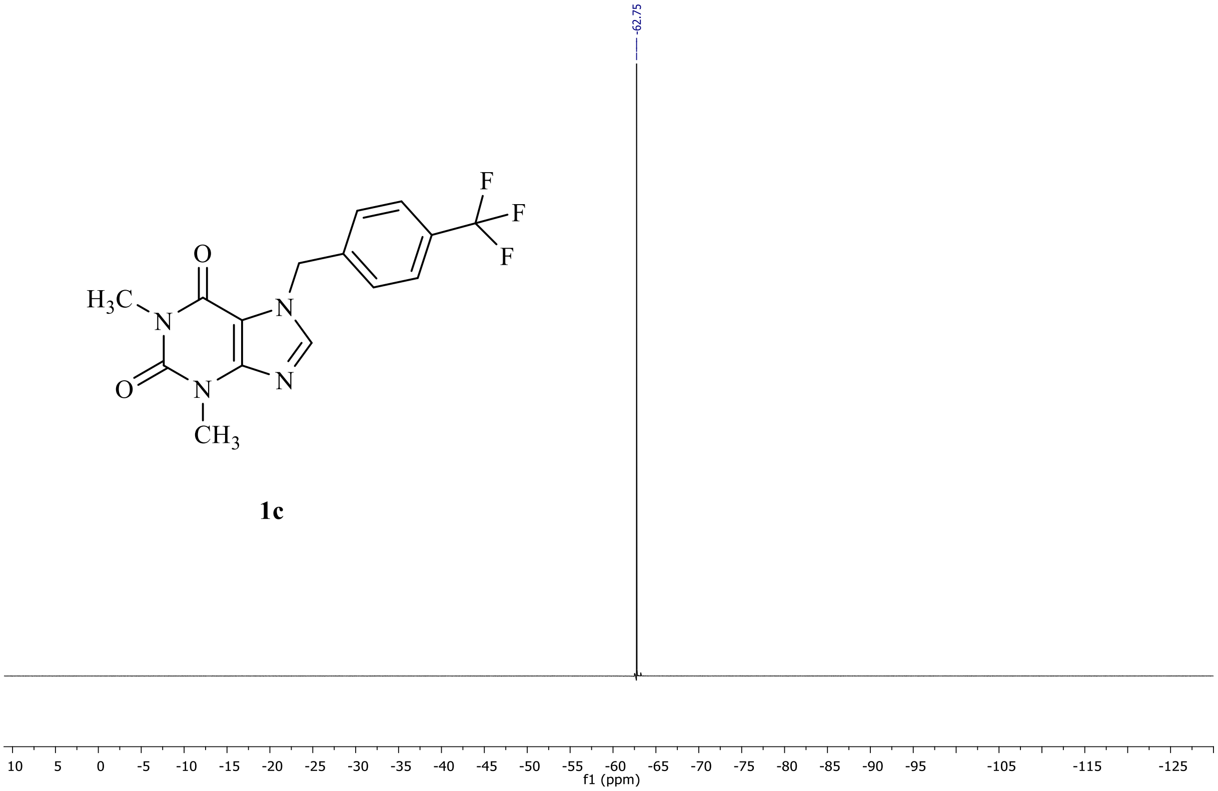

1c

Figure S9. ^{19}F NMR spectrum of **1c** ($CDCl_3$, 25 °C, TMS, 376 MHz).

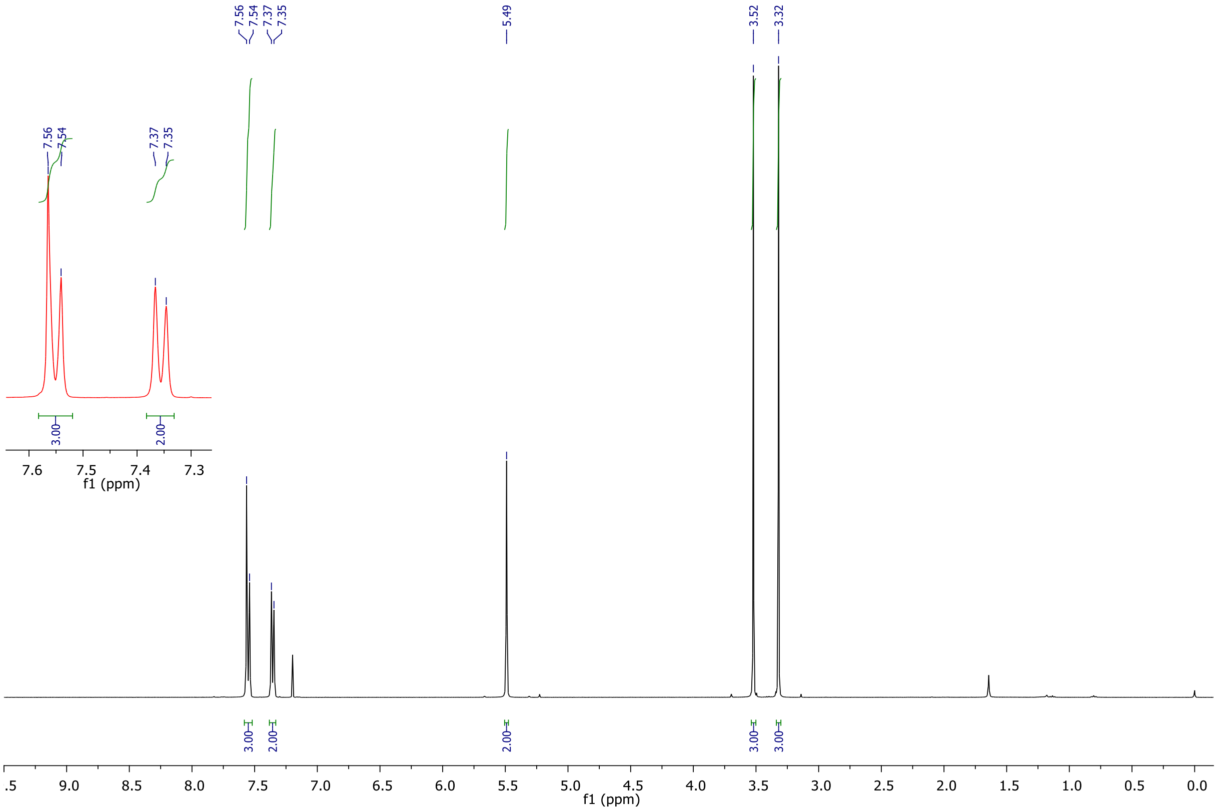

Figure S10. ^{1}H NMR spectrum of **1c** ($CDCl_3$, 25 °C, TMS, 400 MHz).

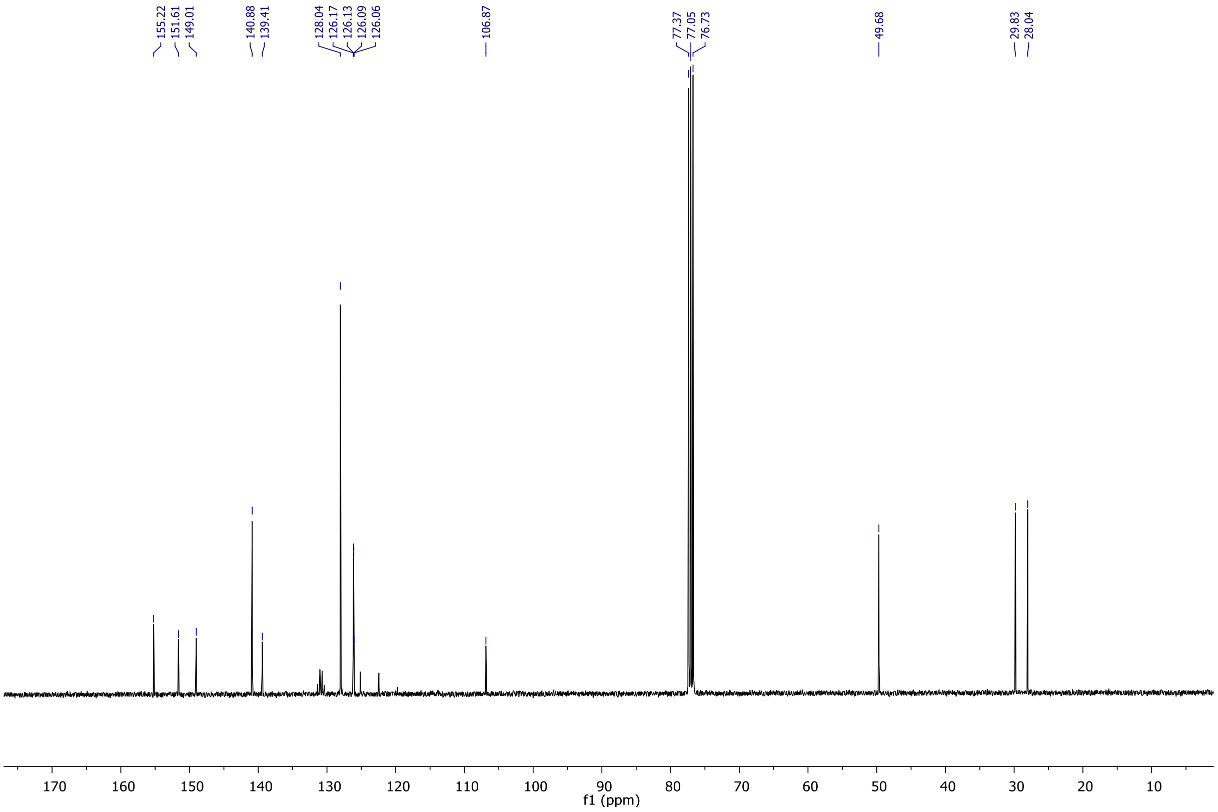

Figure S11. ^{13}C NMR spectrum of **1c** ($CDCl_3$, 25 °C, TMS, 101 MHz).

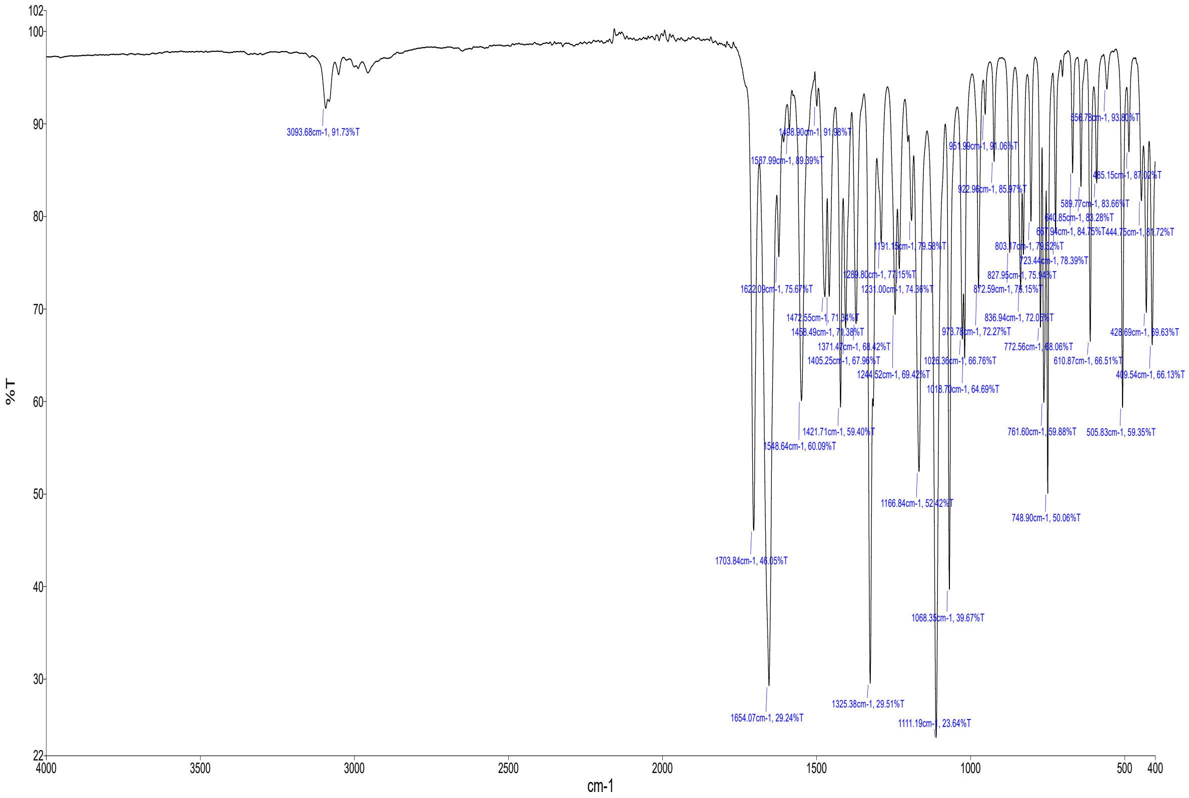

Figure S12. FT-IR spectrum of **1c**.

1,3-Dimethyl-7-(3,5-bis(trifluoromethyl)benzyl)-3,7-dihydro-1*H*-purine-2,6-dione (1d)

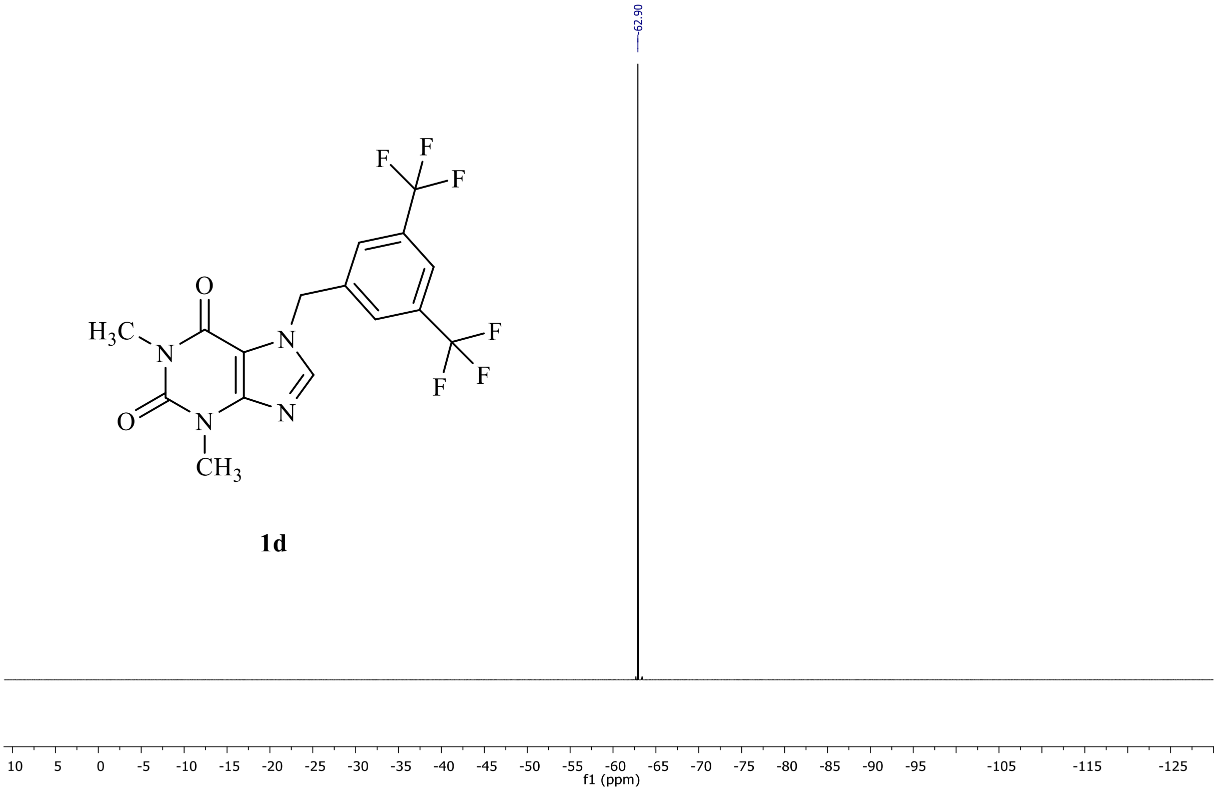

Figure S13. ^{19}F NMR spectrum of **1d** ($CDCl_3$, 25 °C, TMS, 376 MHz).

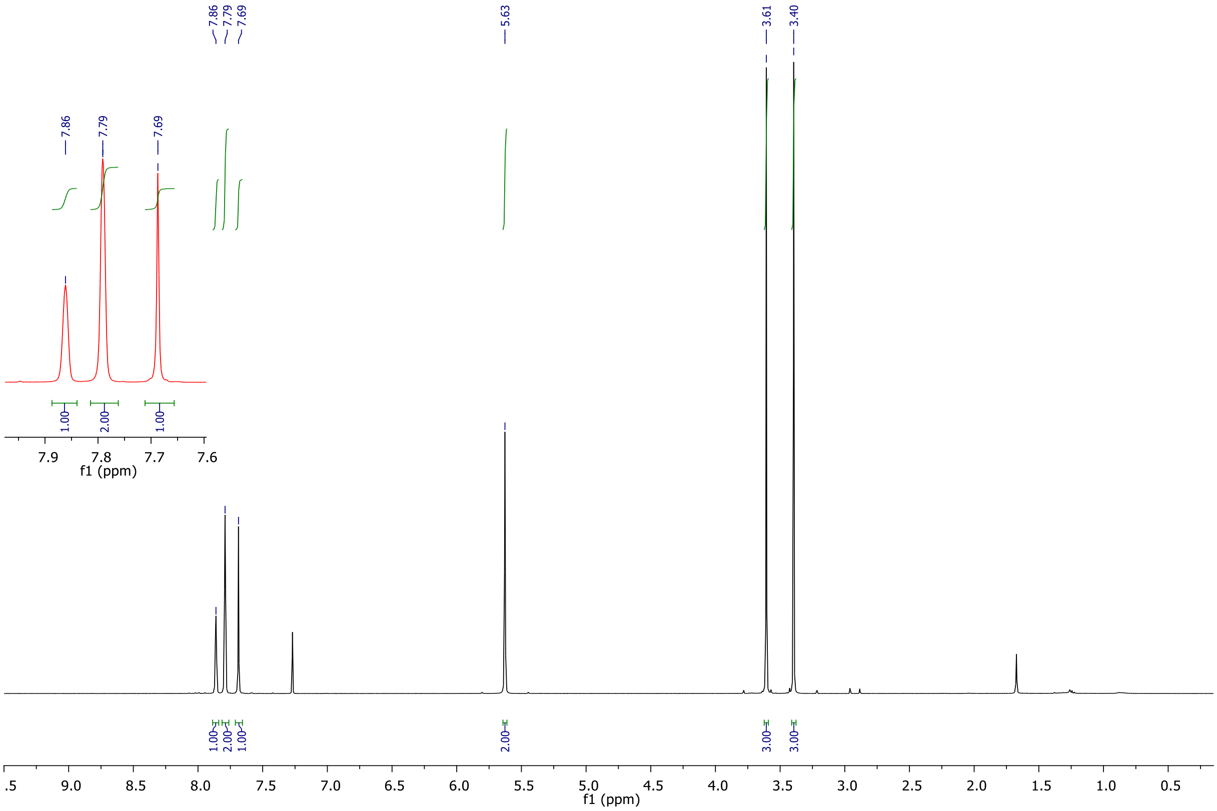

Figure S14. ^{1}H NMR spectrum of **1d** ($CDCl_3$, 25 °C, TMS, 400 MHz).

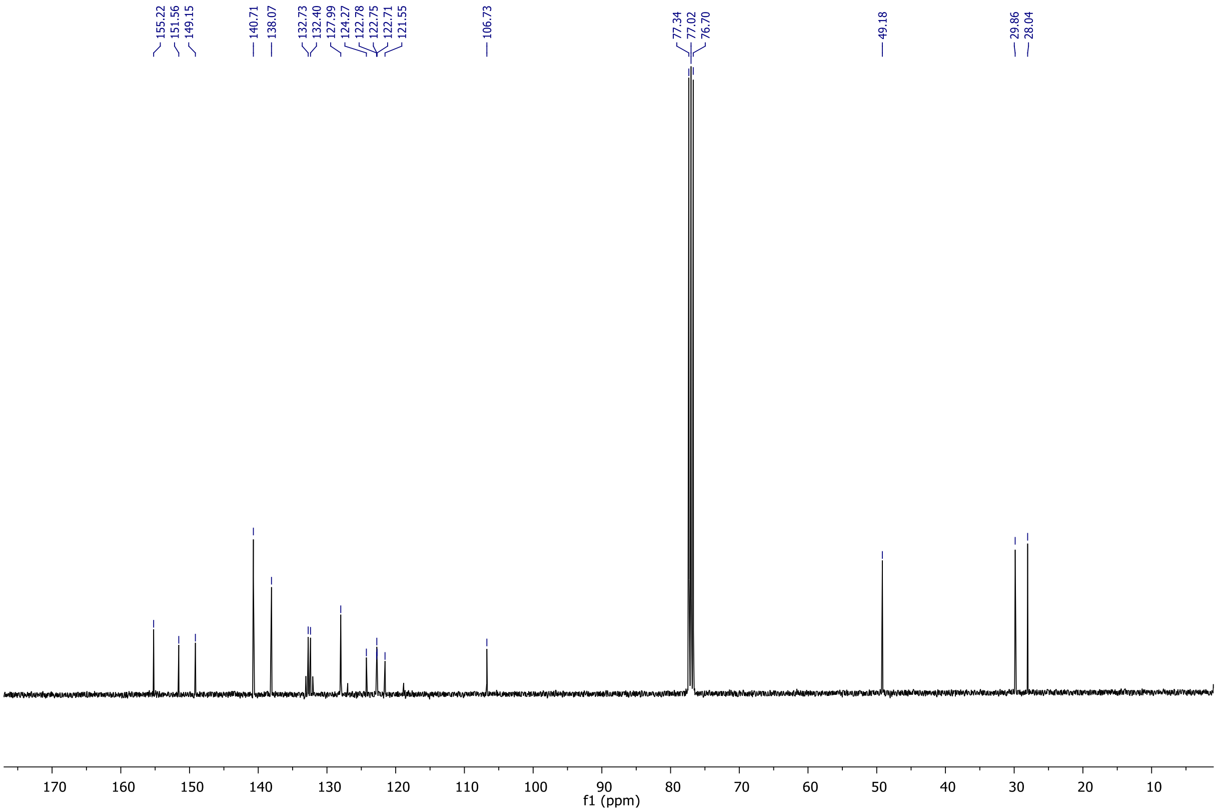

Figure S15. ${}^{13}C$ NMR spectrum of **1d** ($CDCl_3$, 25 °C, TMS, 101 MHz).

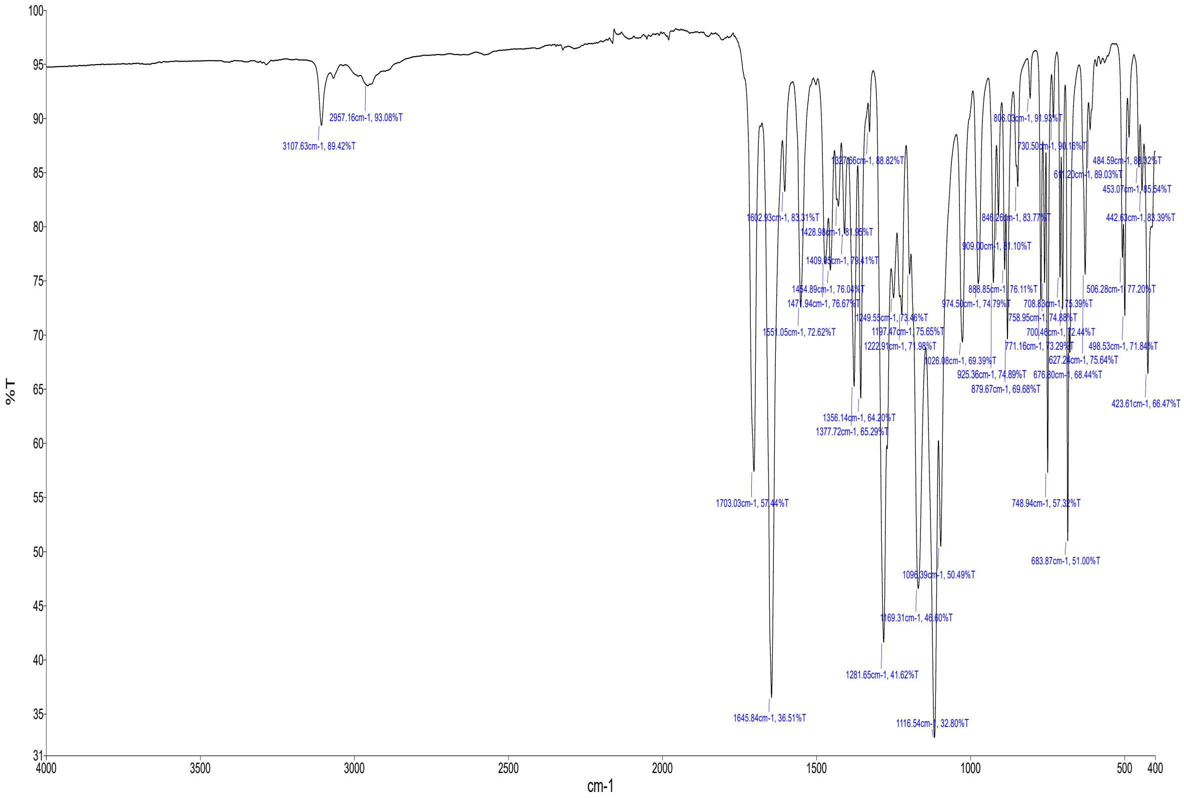

Figure S16. FT-IR spectrum of **1d**.

1,3-Dimethyl-7-(4-(trifluoromethoxy)benzyl)-3,7-dihydro-1*H*-purine-2,6-dione (1e)

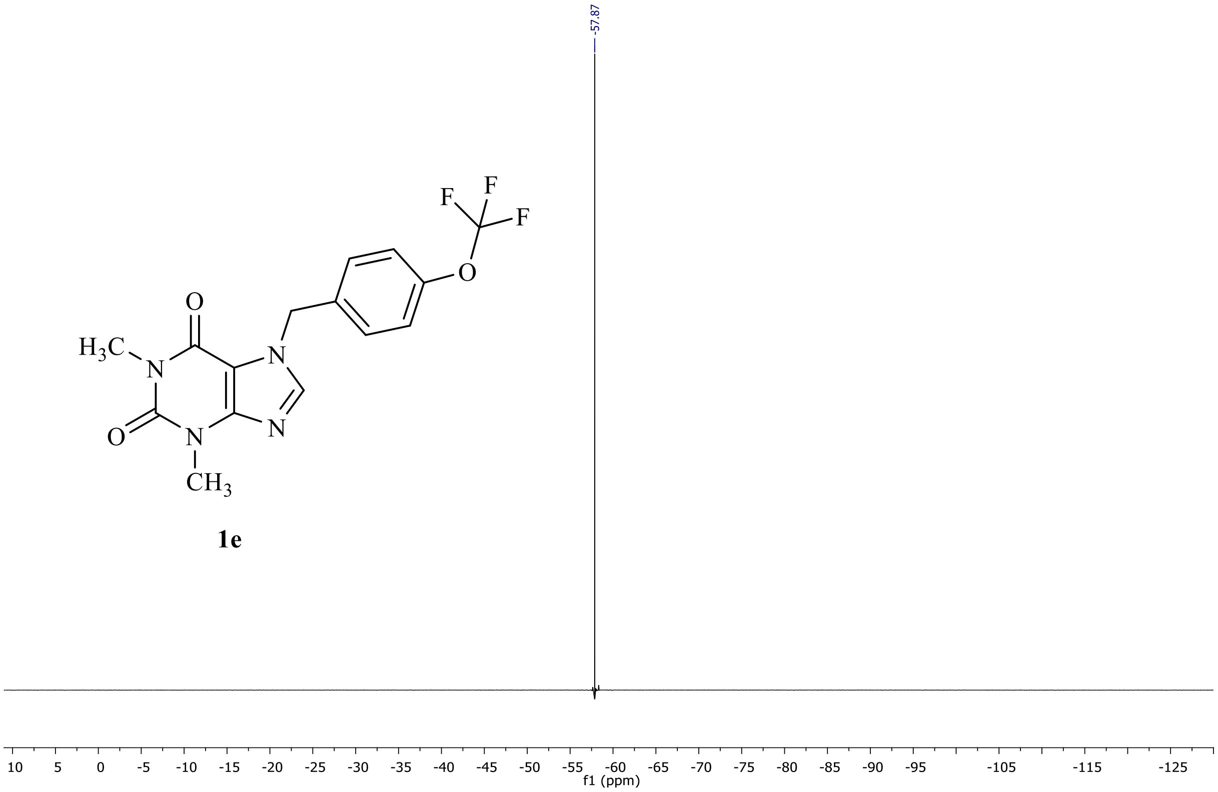

Figure S17. ^{19}F NMR spectrum of **1e** ($CDCl_3$, 25 °C, TMS, 376 MHz).

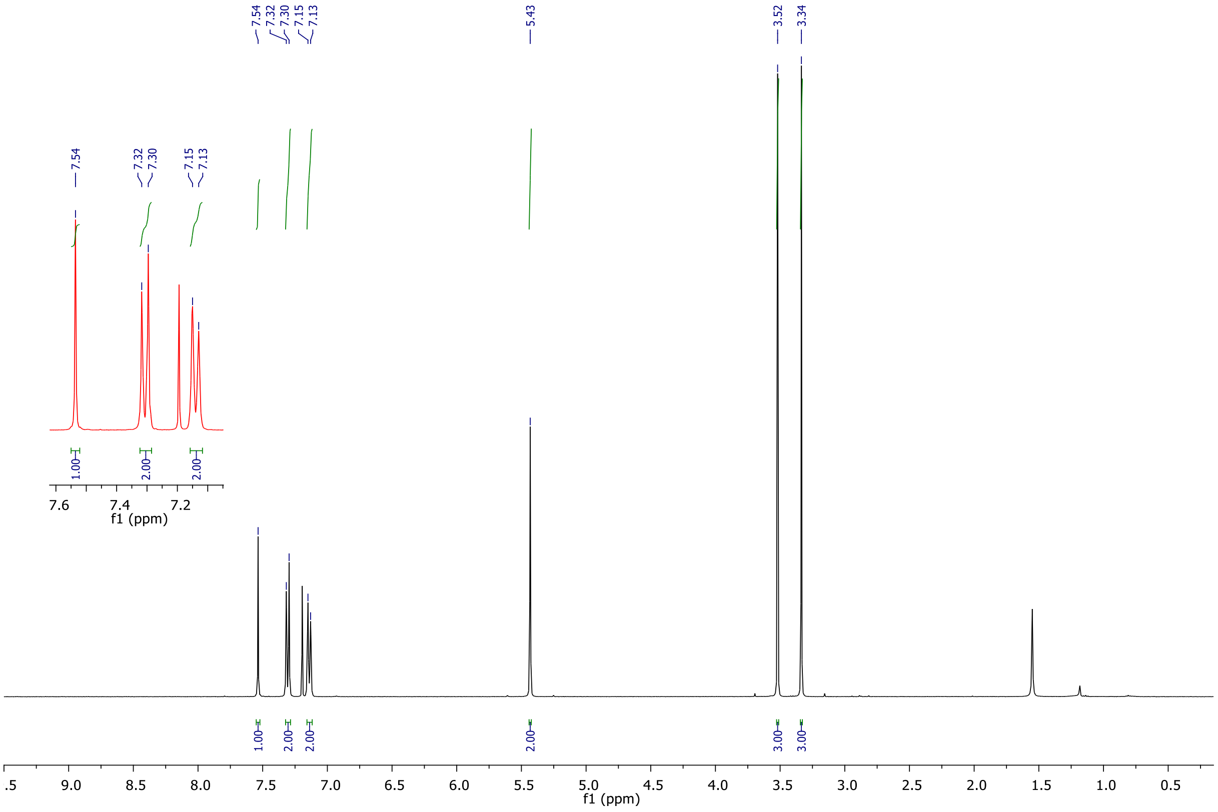

Figure S18. ^{1}H NMR spectrum of **1e** ($CDCl_3$, 25 °C, TMS, 400 MHz).

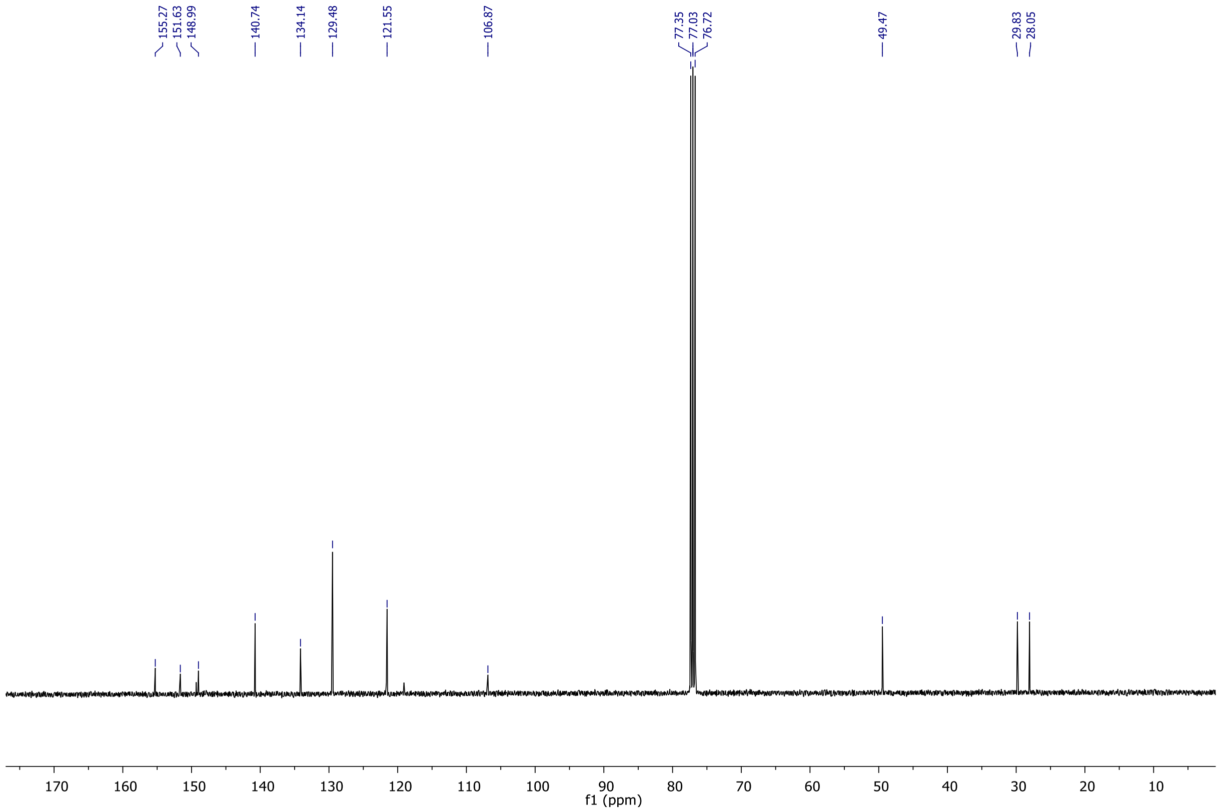

Figure S19. ^{13}C NMR spectrum of **1e** ($CDCl_3$, 25 °C, TMS, 101 MHz).

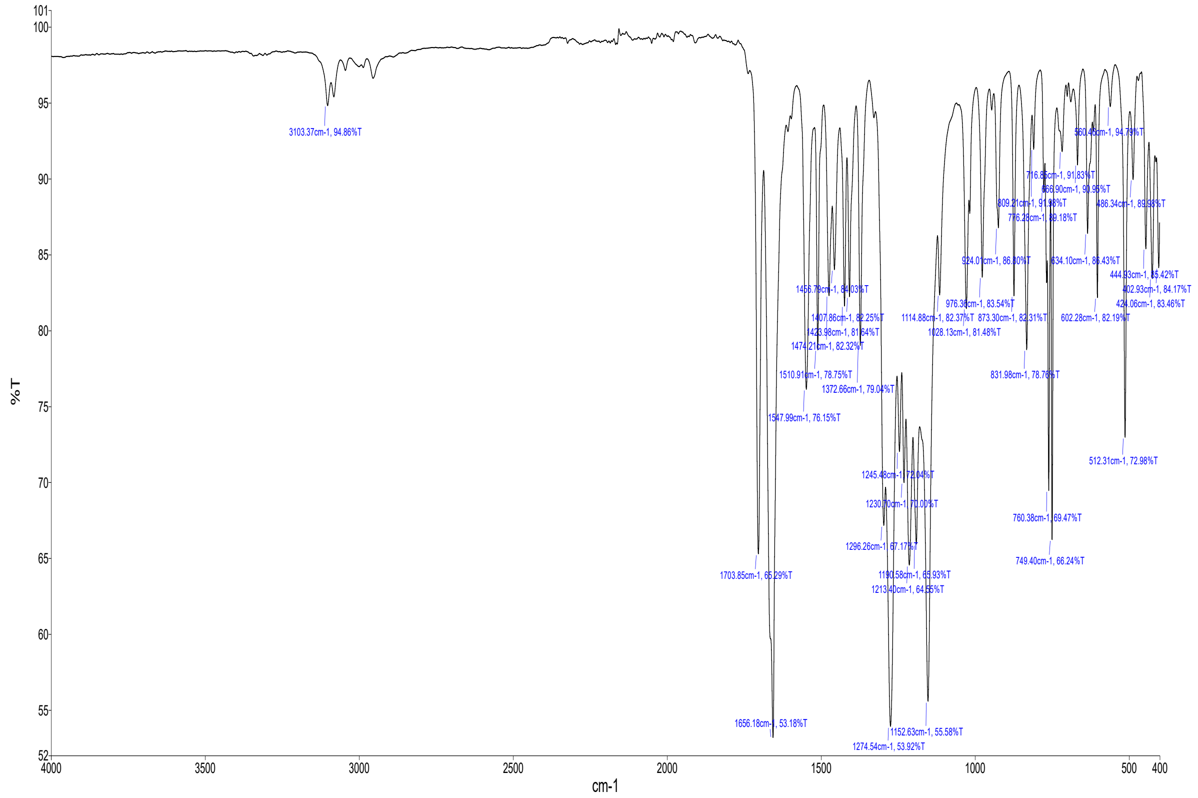

Figure S20. FT-IR spectrum of **1e**.

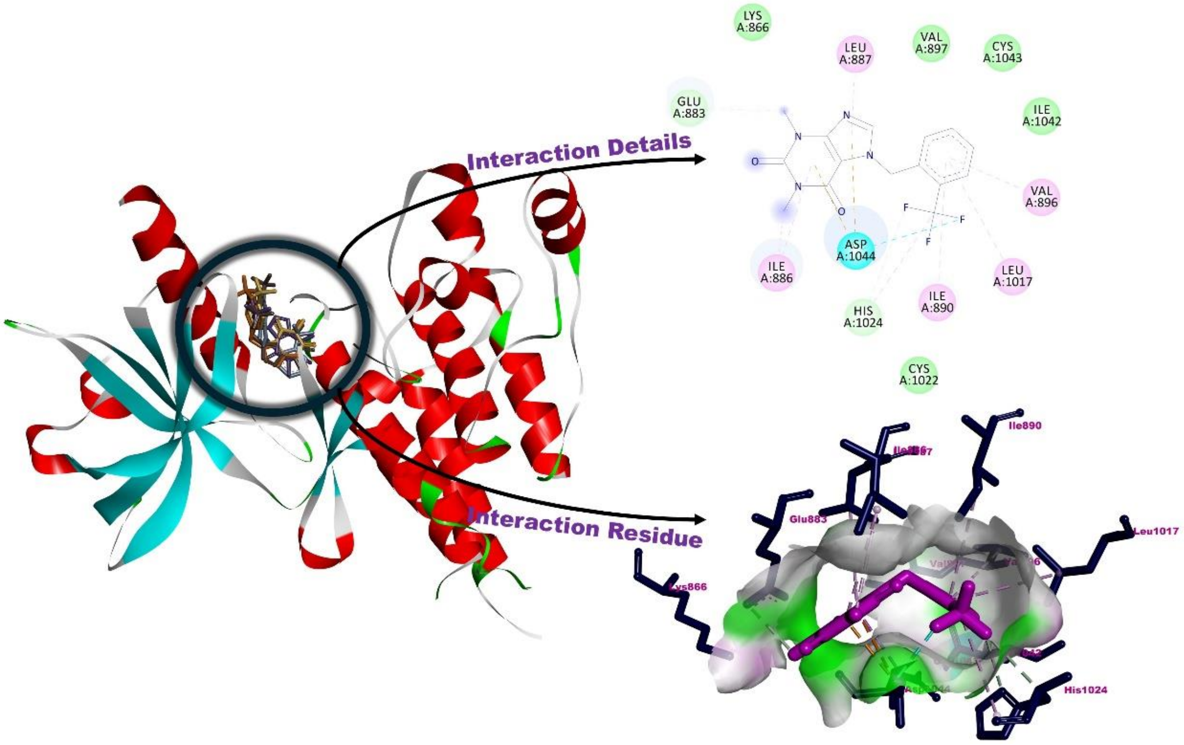

Figure S21. Interaction Residue and Details of **1a** against VEGFR2.

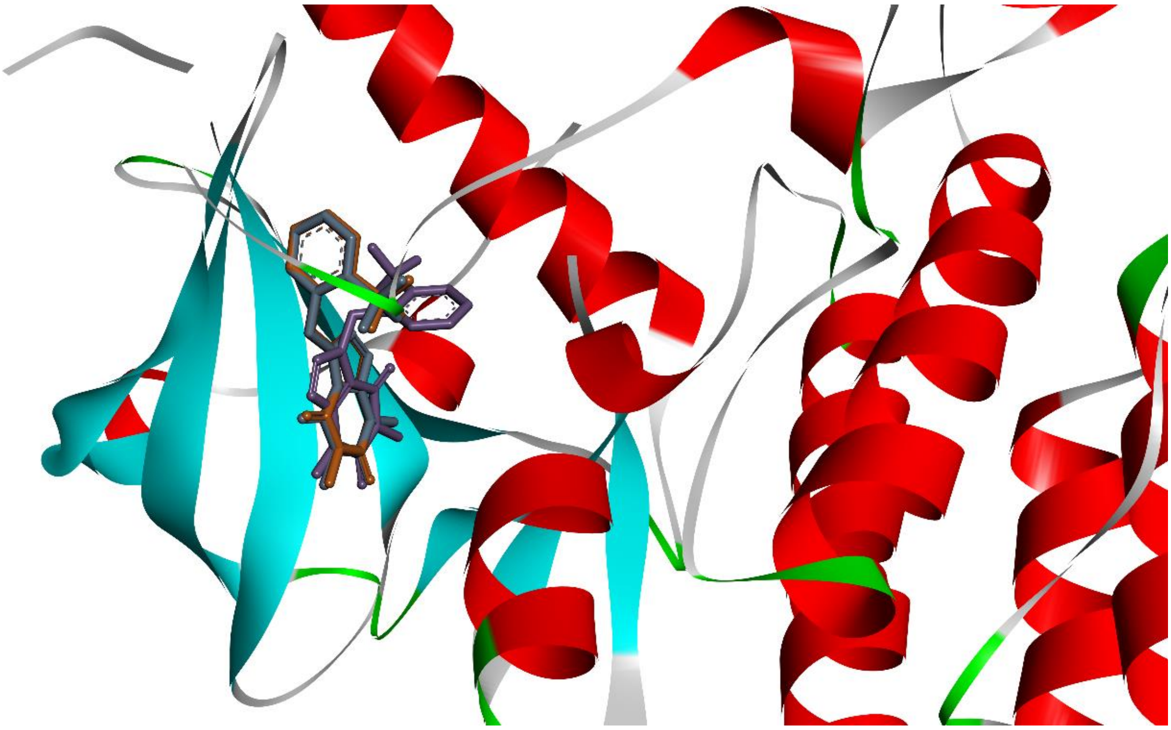

Figure S22. The overlaps of the molecular docking poses of **1a** against VEGFR2.

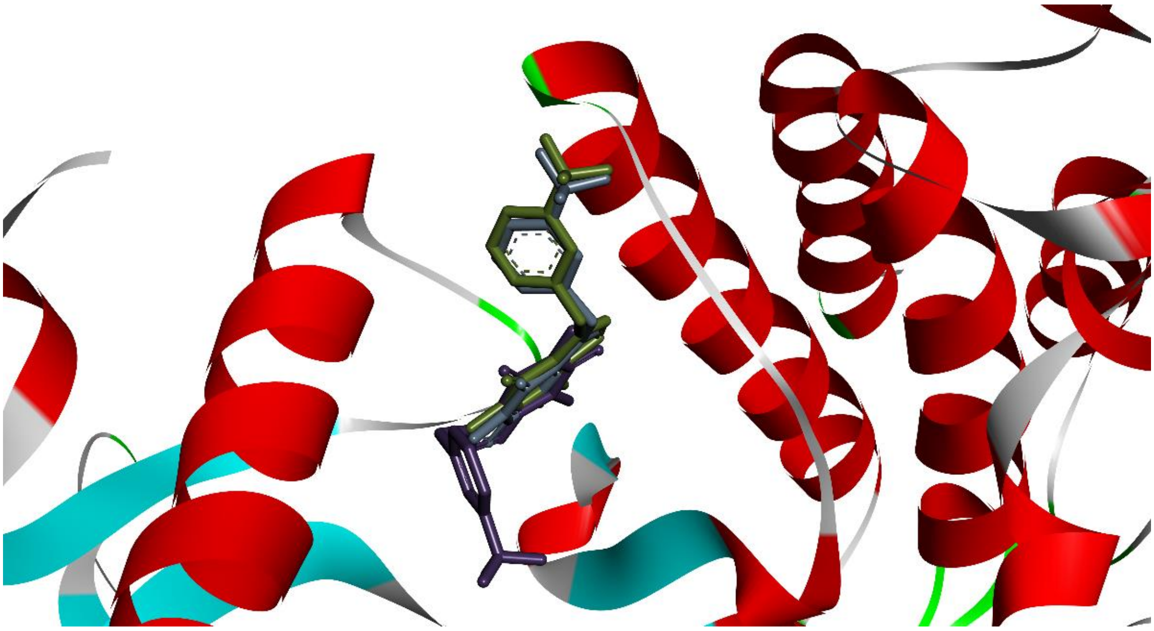

Figure S23. The overlaps of the molecular docking poses of **1b** against VEGFR2.

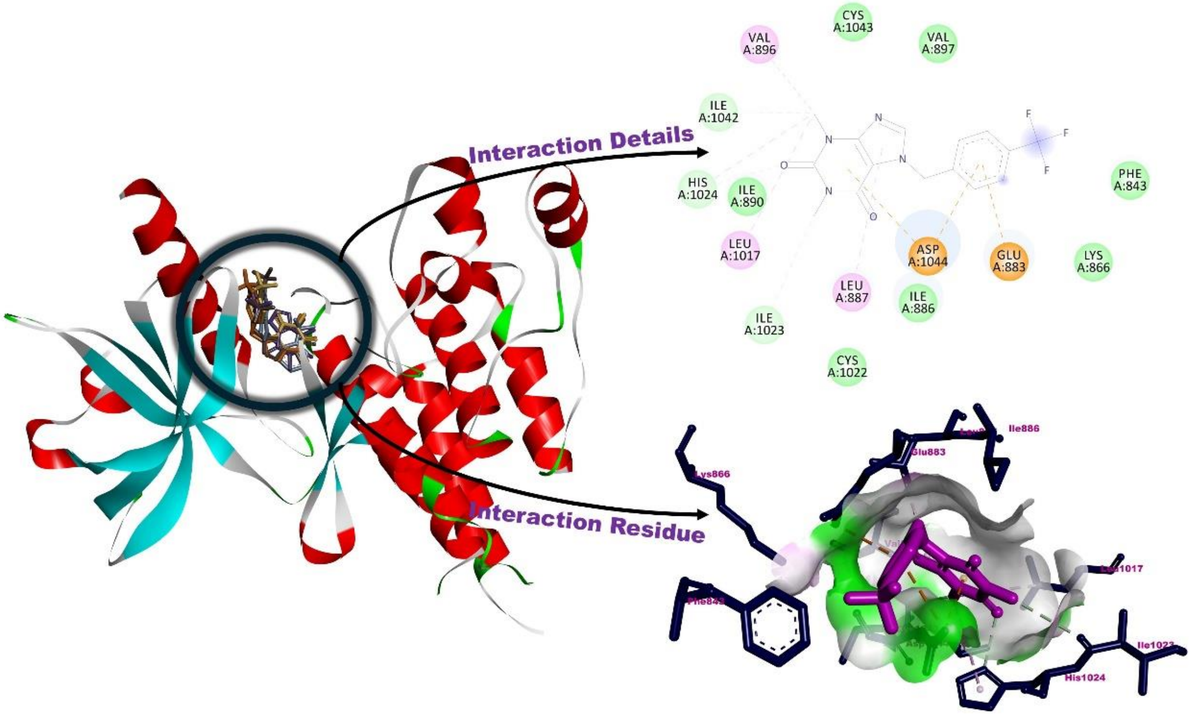

Figure S24. Interaction Residue and Details of **1c** against VEGFR2.

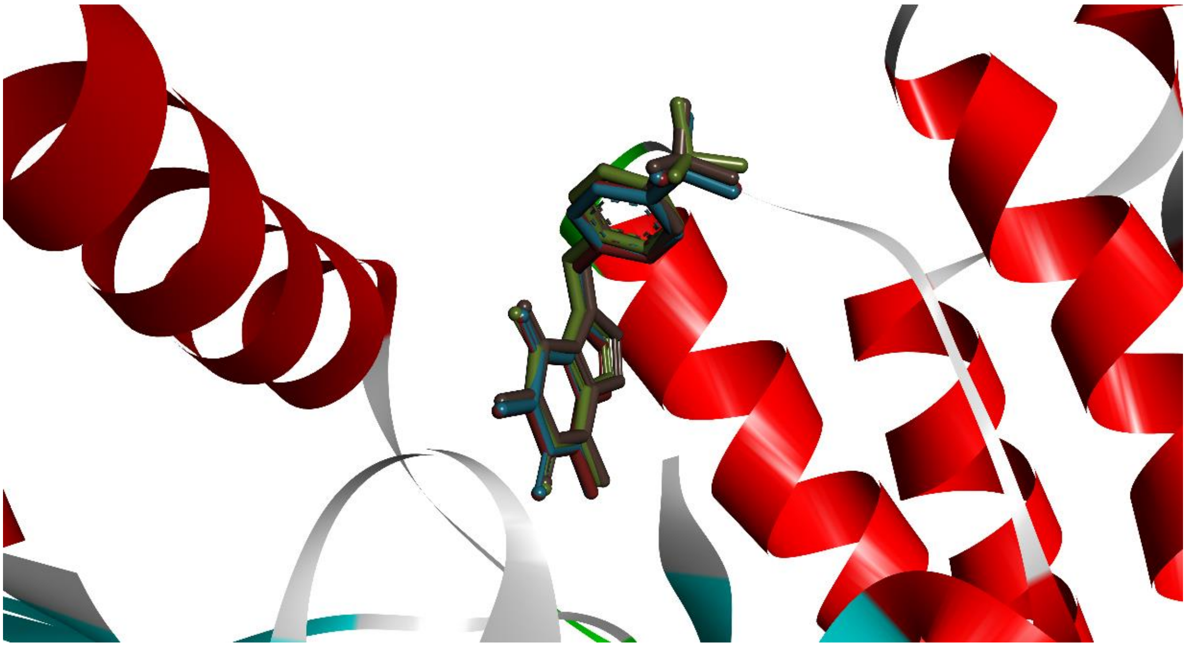

Figure S25. The overlaps of the molecular docking poses of **1c** against VEGFR2.

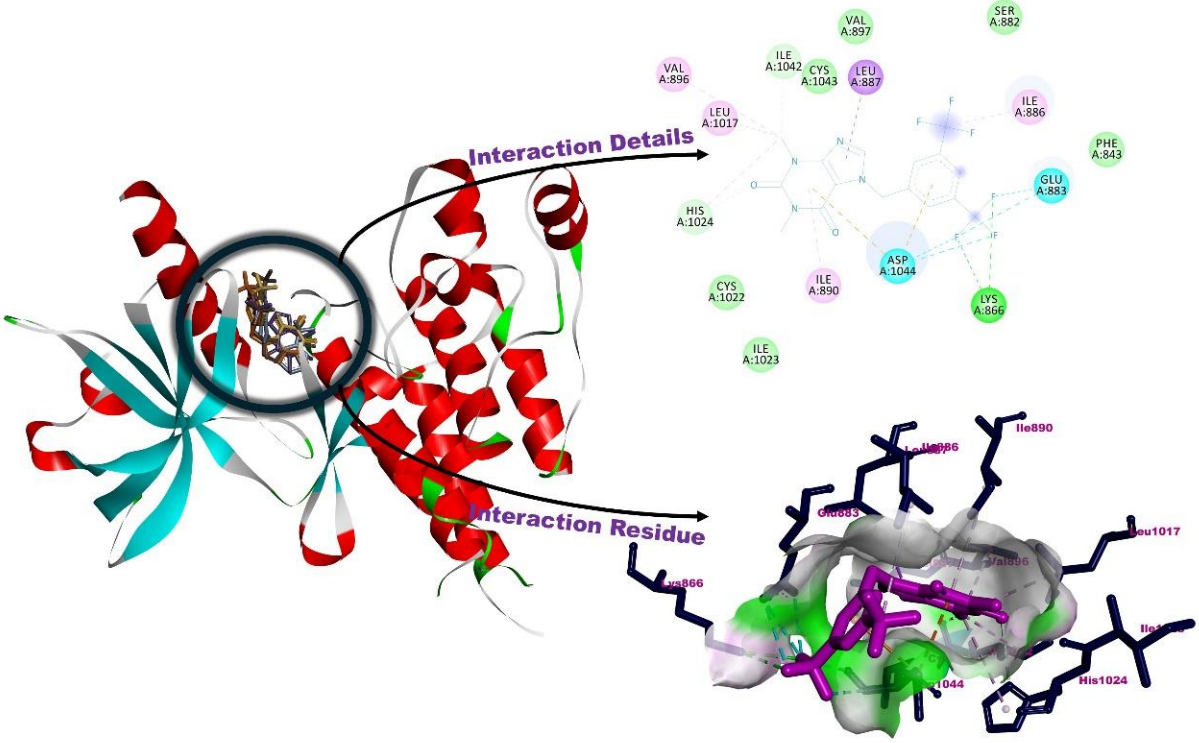

Figure S26. Interaction Residue and Details of **1d** against VEGFR2.

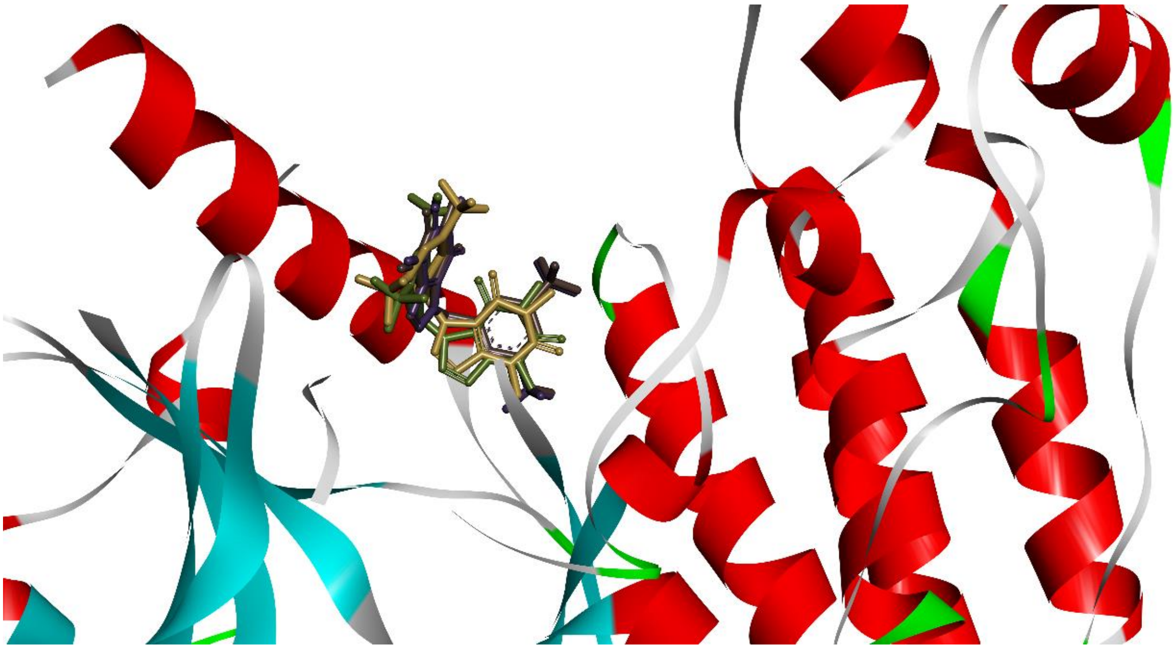

Figure S27. The overlaps of the molecular docking poses of **1d** against VEGFR2.

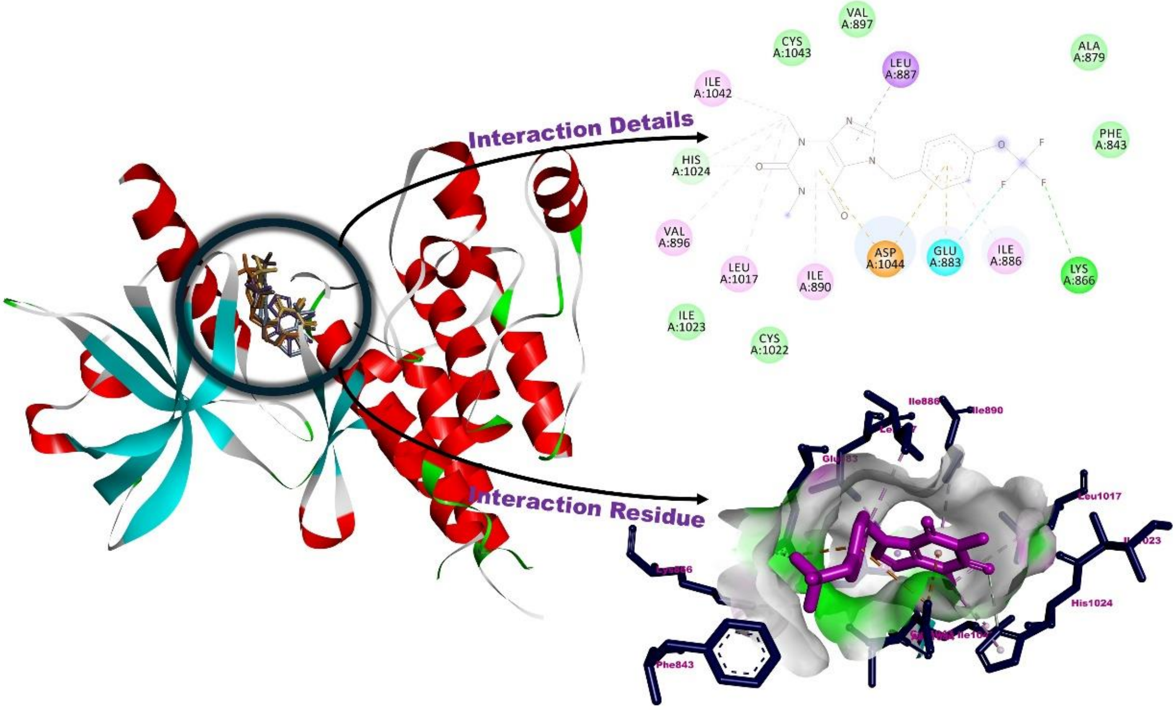

Figure S28. Interaction Residue and Details of **1e** against VEGFR2.

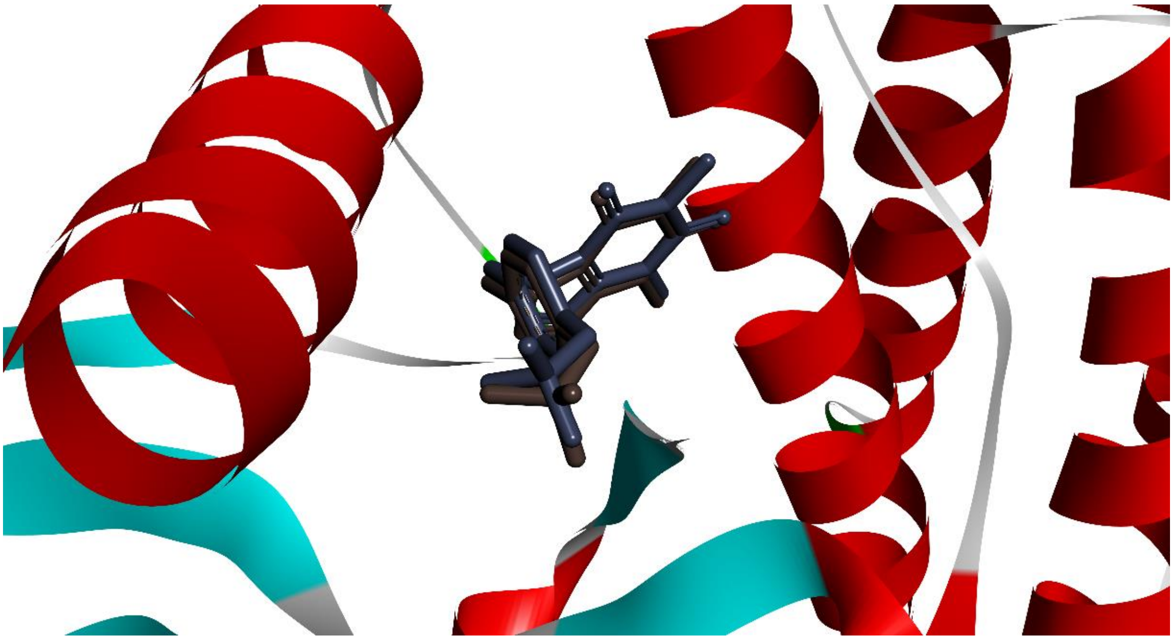

Figure S29. The overlaps of the molecular docking poses of **1e** against VEGFR2.

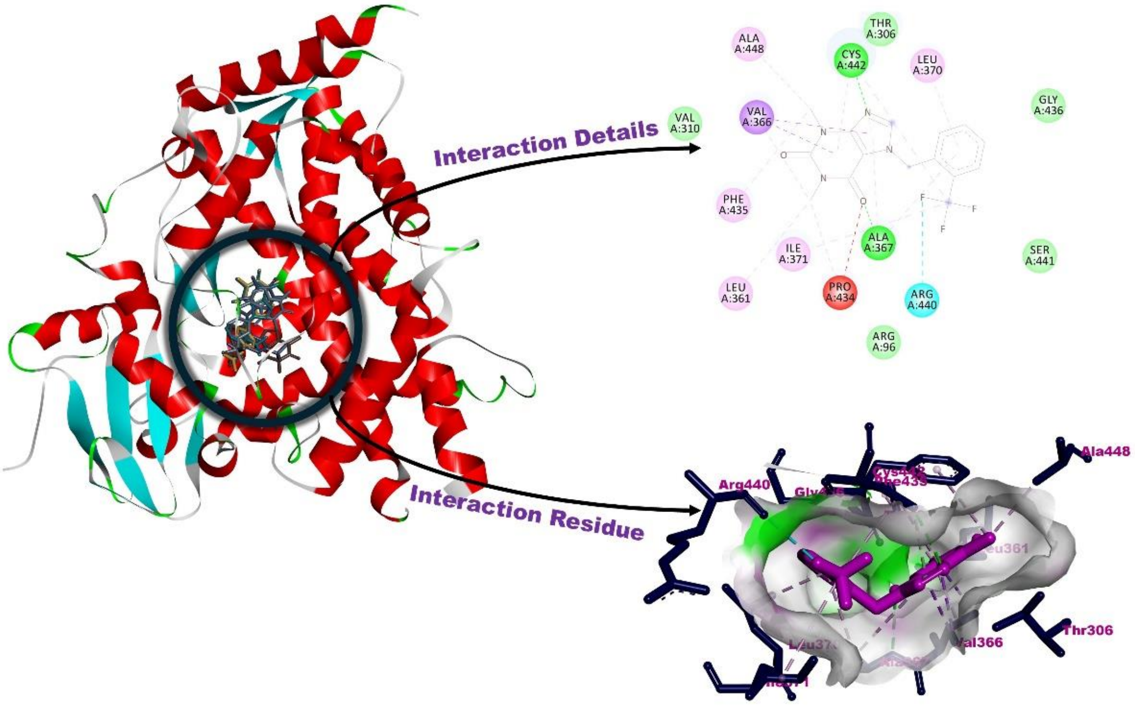

Figure S30. Interaction Residue and Details of **1a** against Human Cytochrome P450.

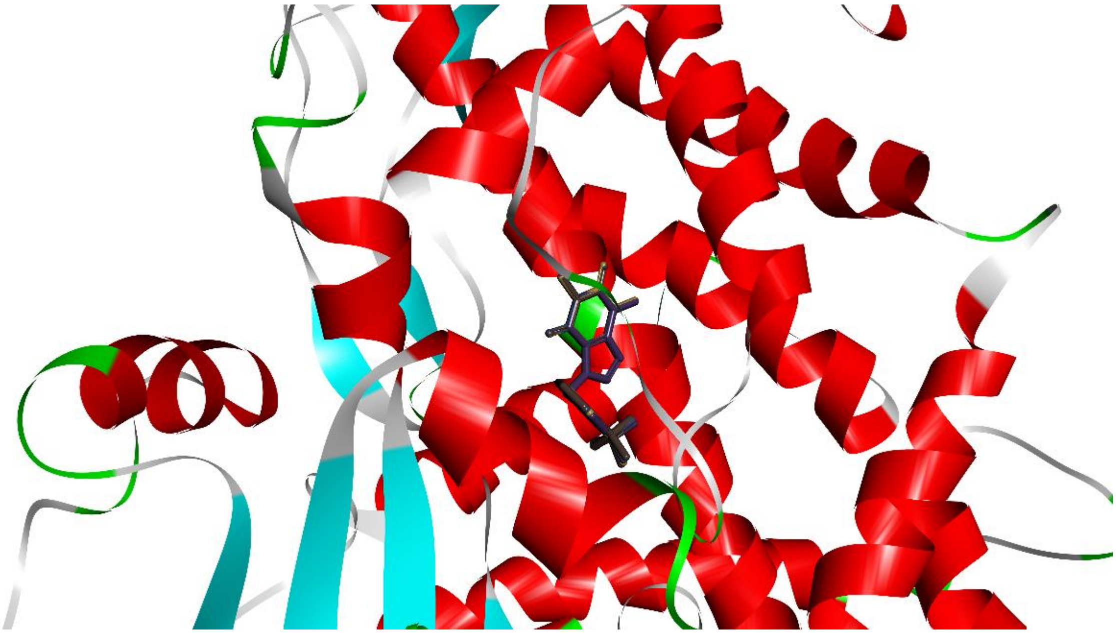

Figure S31. The overlaps of the molecular docking poses of **1a** against Human Cytochrome P450.

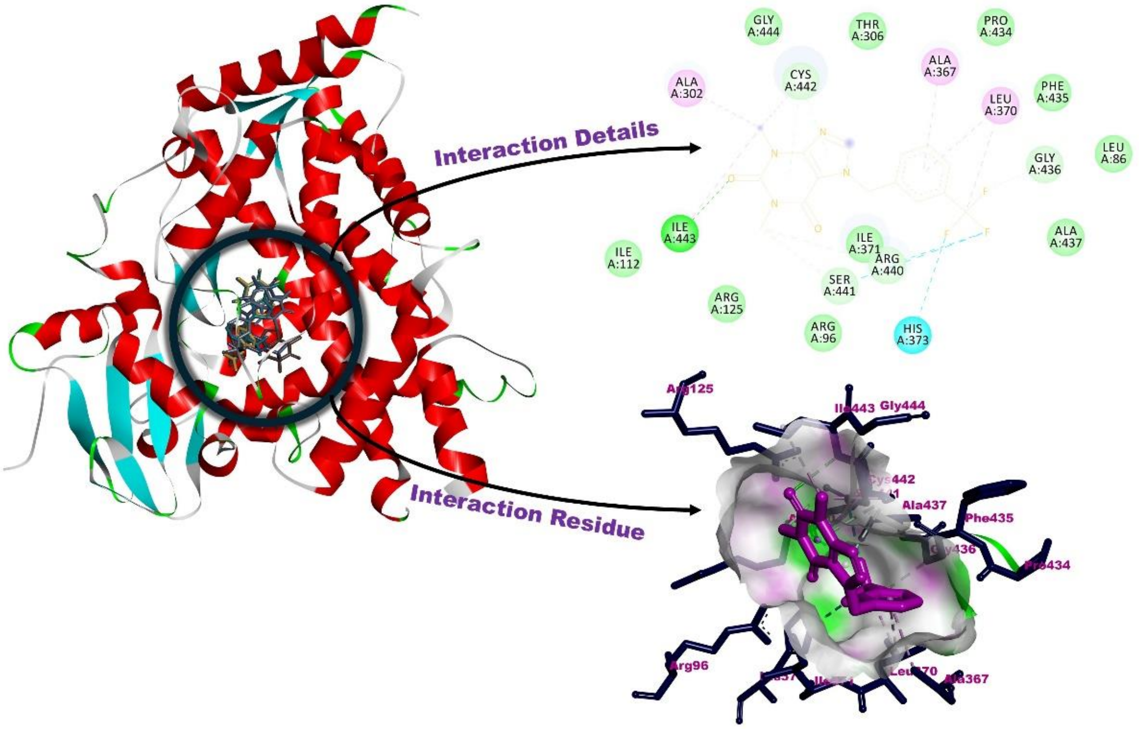

Figure S32. Interaction Residue and Details of **1b** against Human Cytochrome P450.

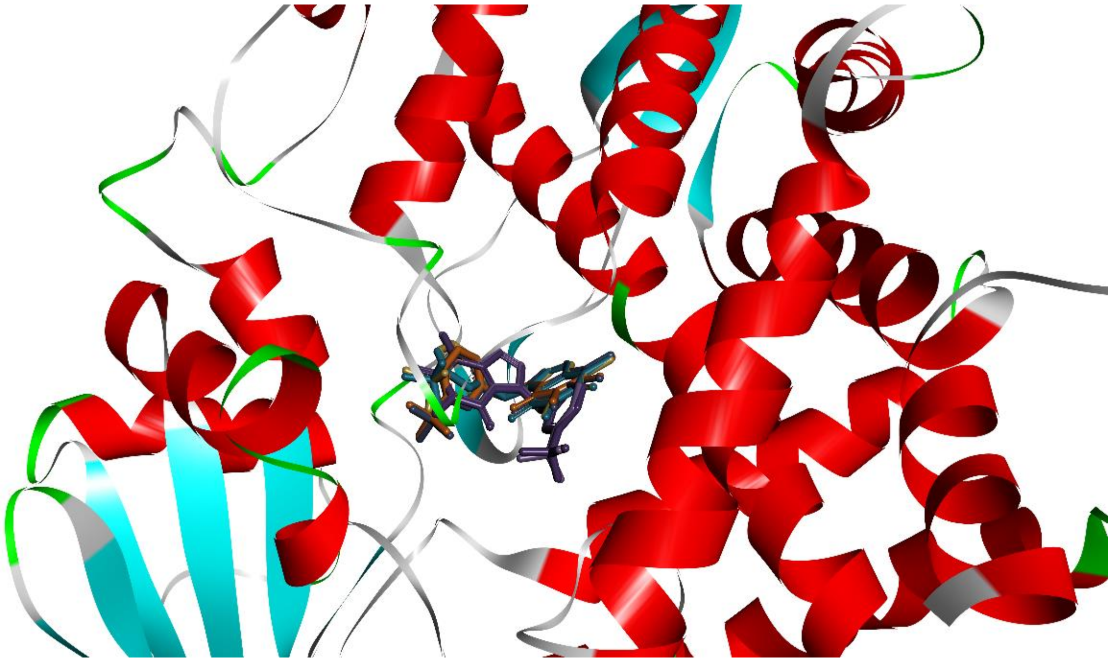

Figure S33. The overlaps of the molecular docking poses of **1b** against Human Cytochrome P450.

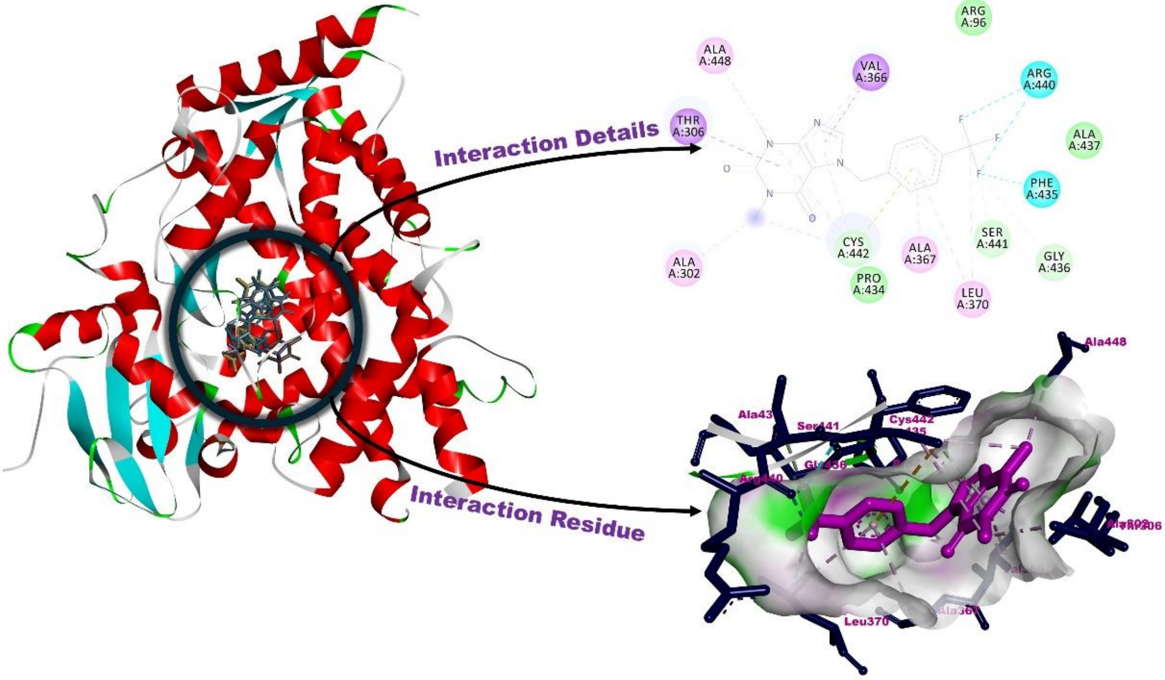

Figure S34. Interaction Residue and Details of **1c** against Human Cytochrome P450.

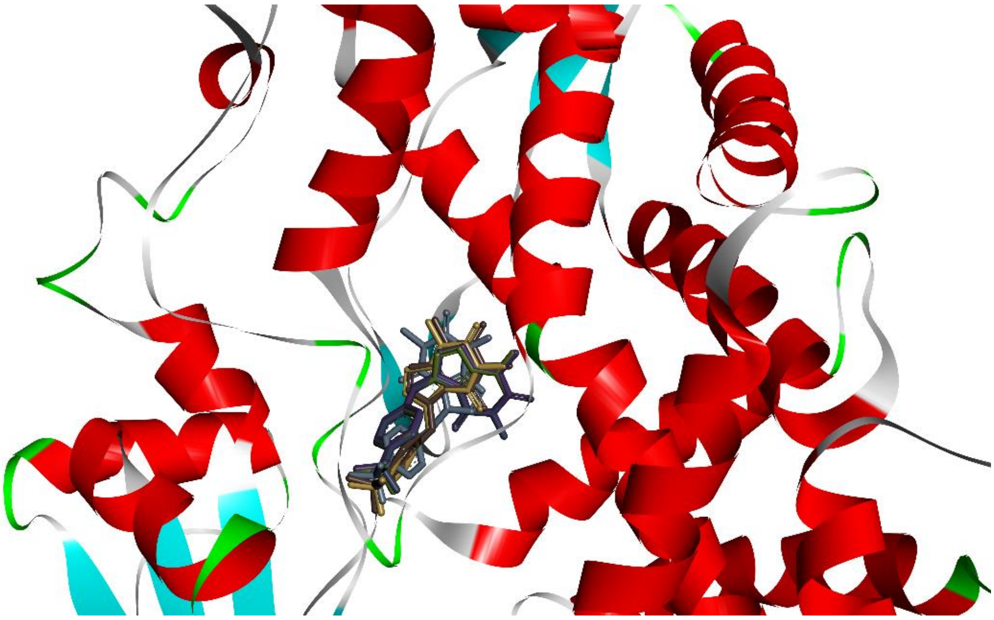

Figure S35. The overlaps of the molecular docking poses of **1c** against Human Cytochrome P450.

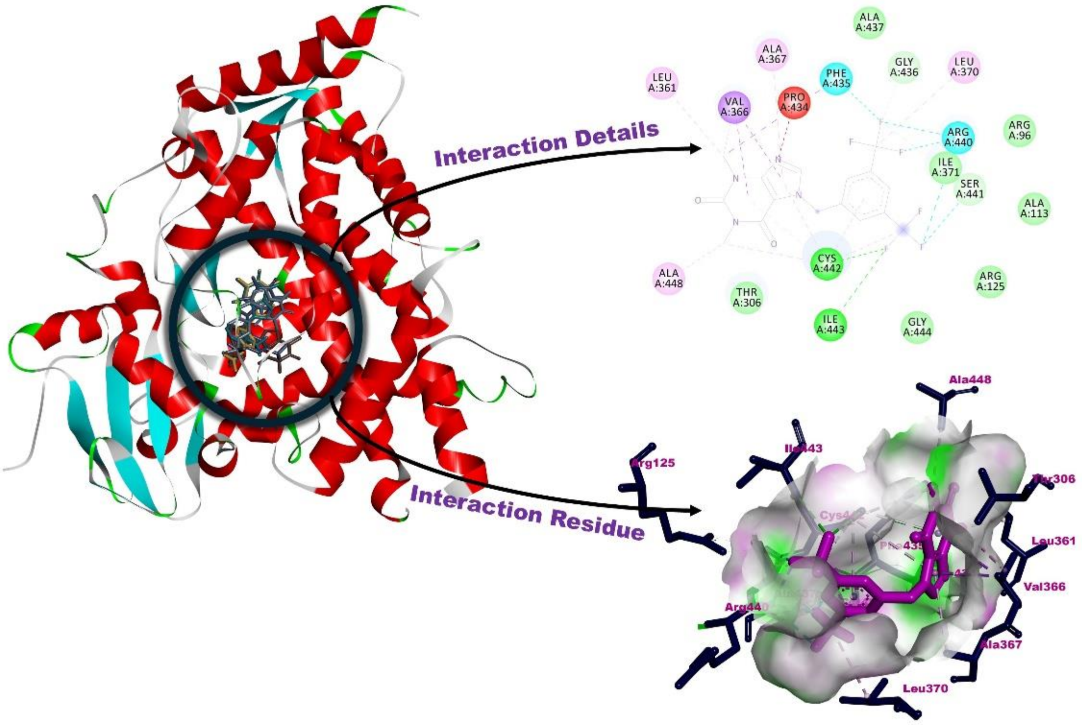

Figure S36. Interaction Residue and Details of **1d** against Human Cytochrome P450.

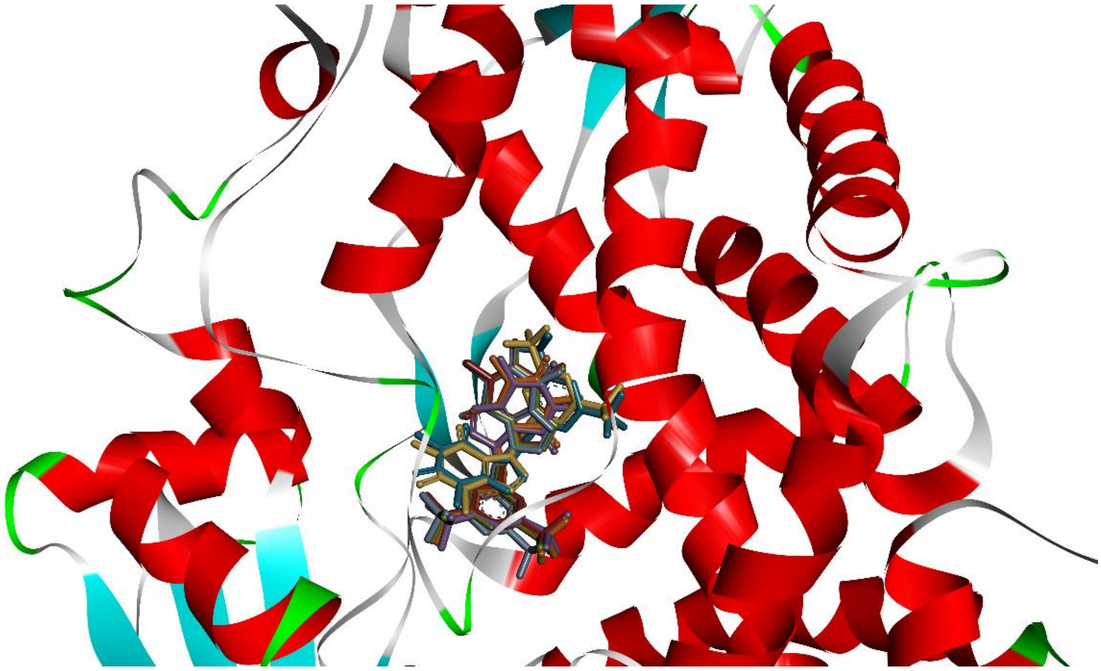

Figure S37. The overlaps of the molecular docking poses of **1d** against Human Cytochrome P450.

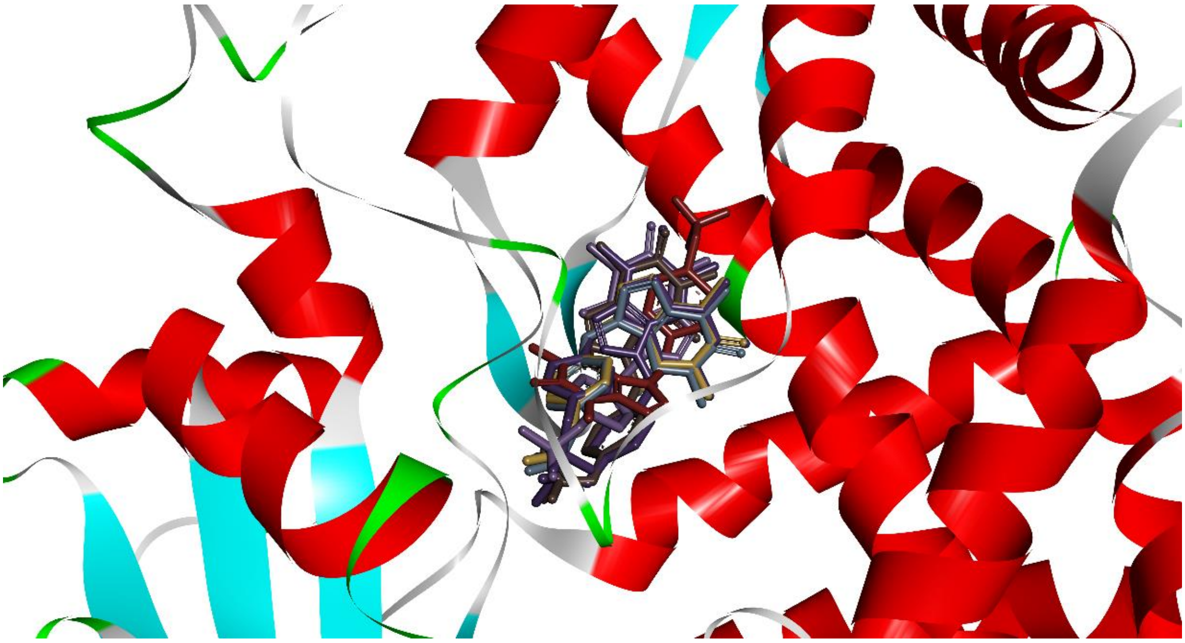

Figure S38. The overlaps of the molecular docking poses of **1e** against Human Cytochrome P450.

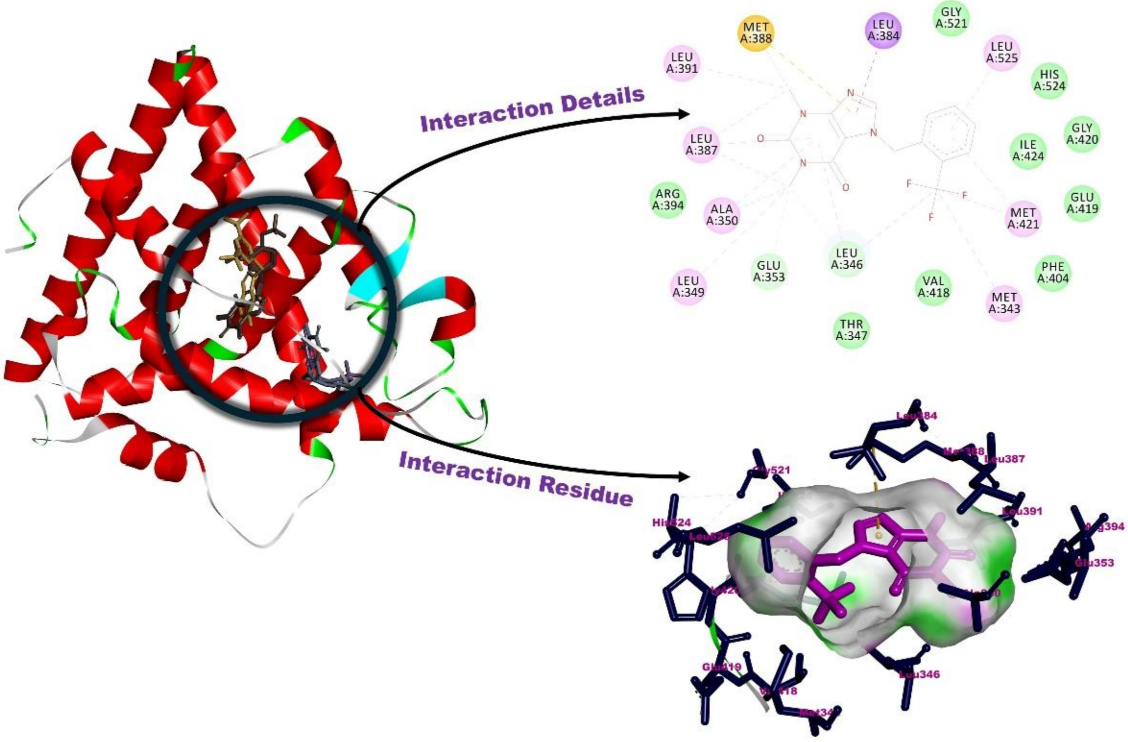

Figure S39. Interaction Residue and Details of **1a** against estrogen receptor.

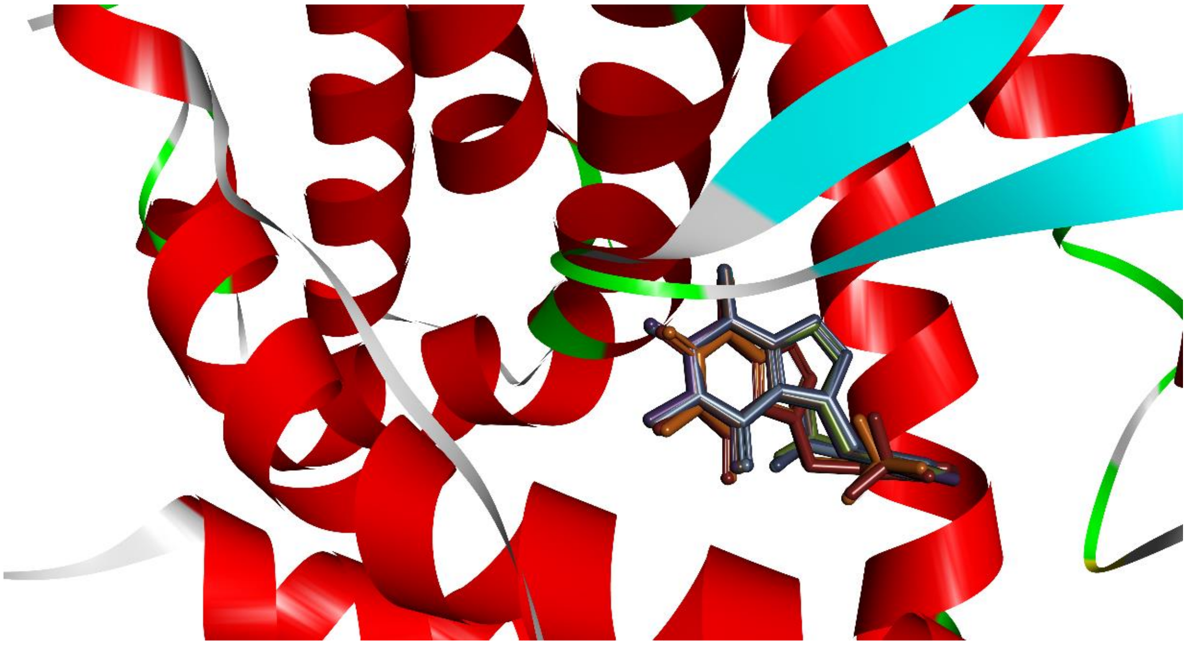

Figure S40. The overlaps of the molecular docking poses of **1a** against estrogen receptor.

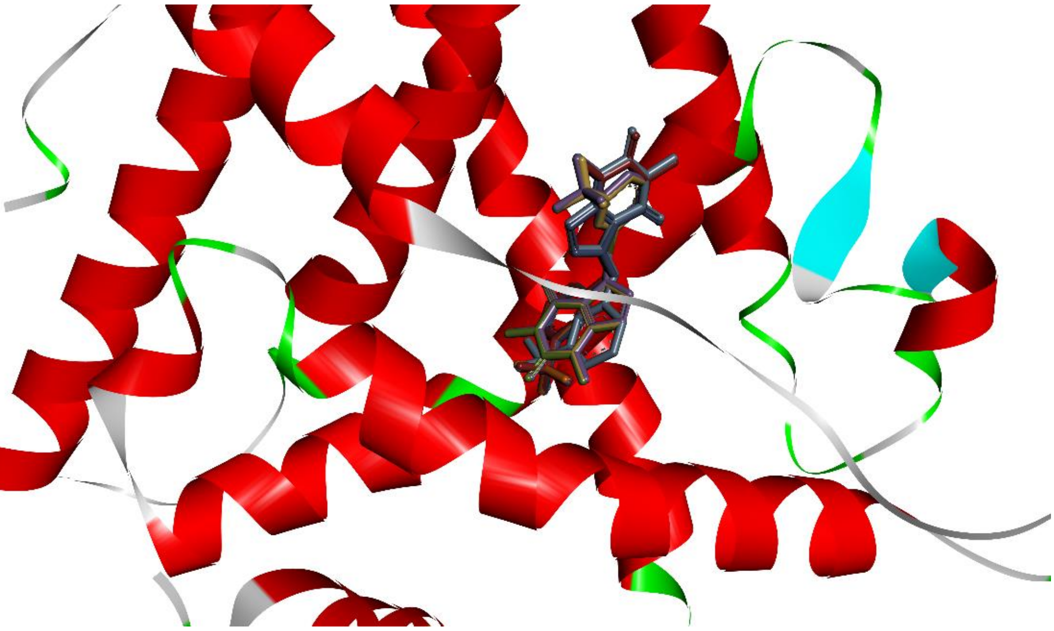

Figure S41. The overlaps of the molecular docking poses of **1b** against estrogen receptor.

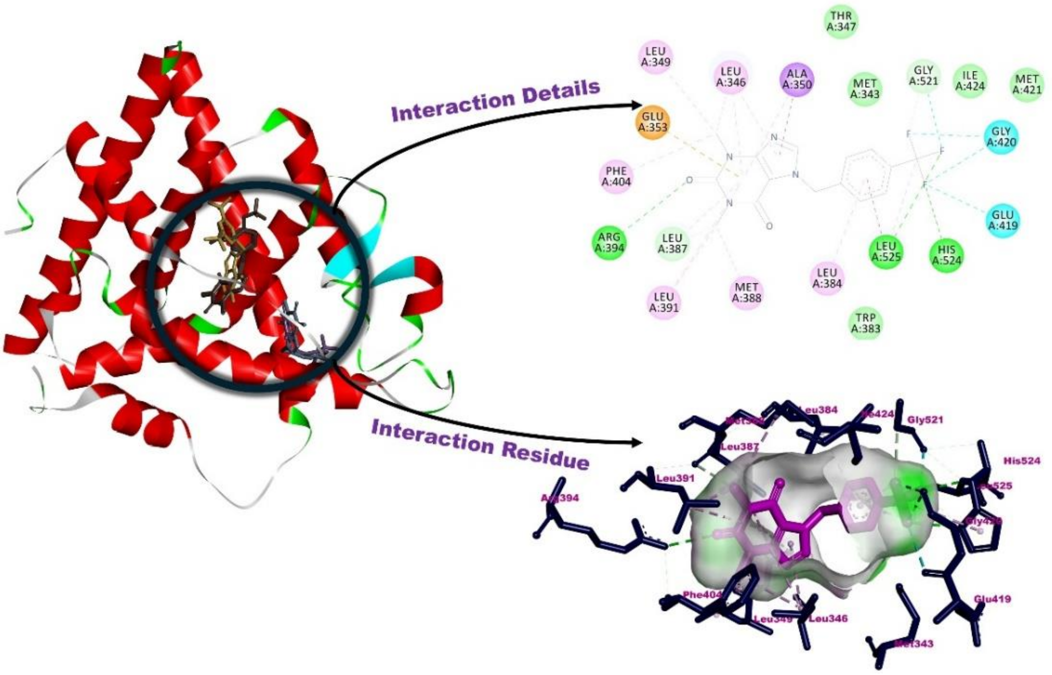

Figure S42. Interaction Residue and Details of **1c** against estrogen receptor.

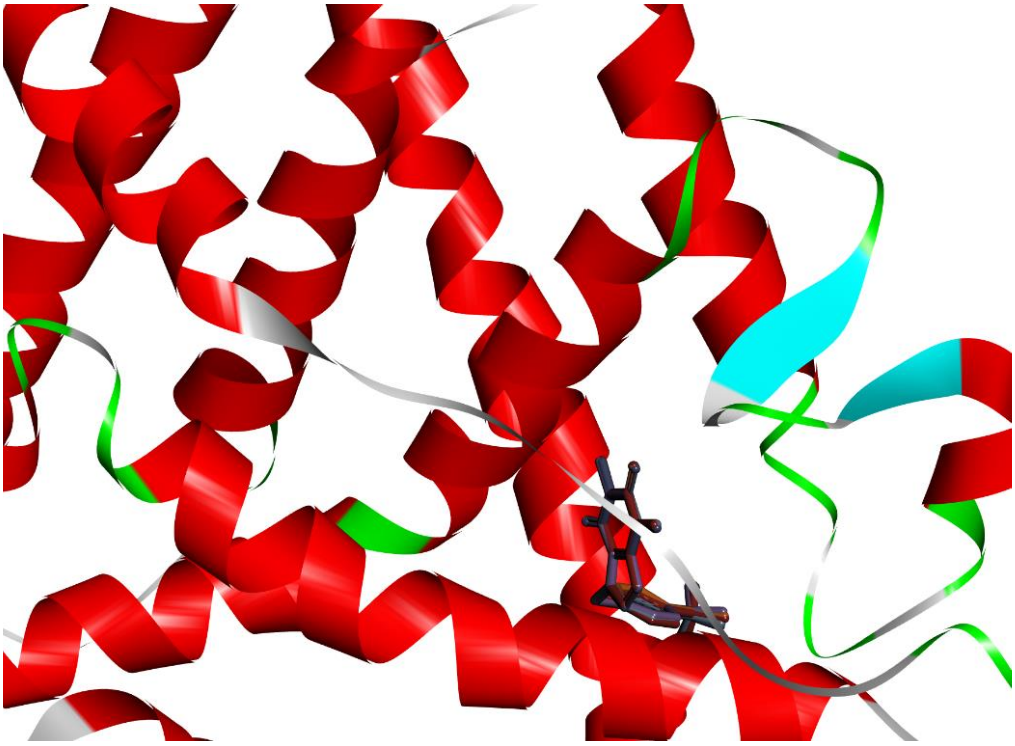

Figure S43. The overlaps of the molecular docking poses of **1c** against estrogen receptor.

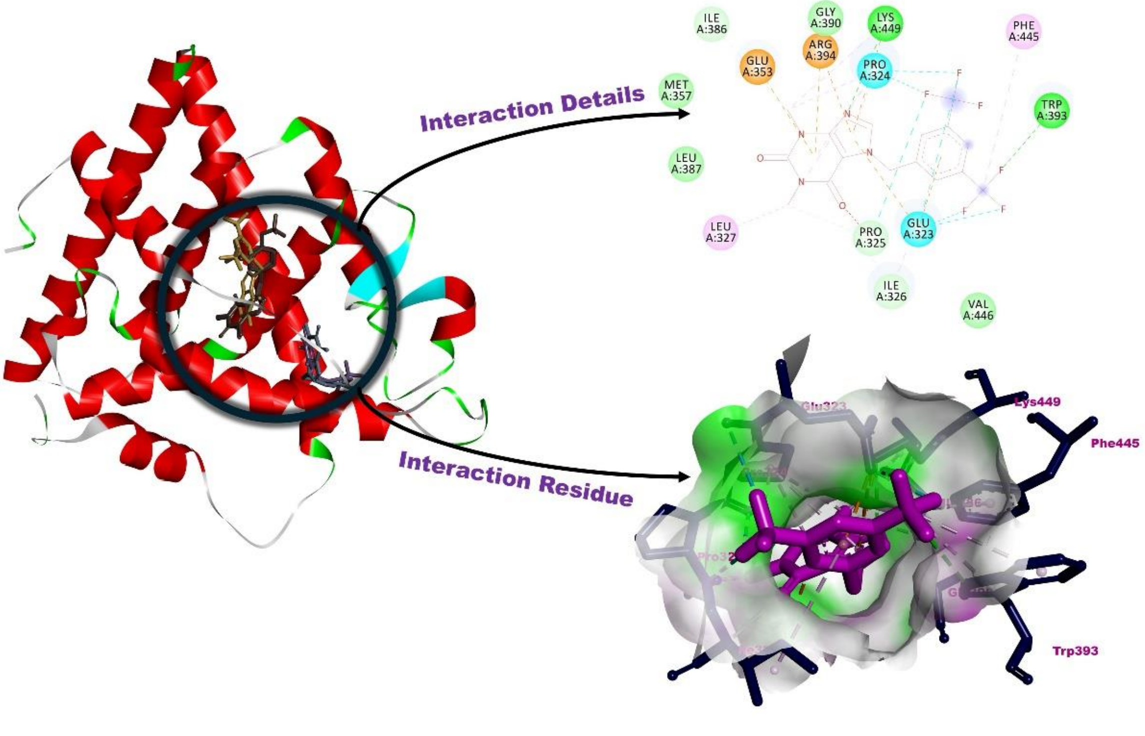

Figure S44. Interaction Residue and Details of **1d** against estrogen receptor.

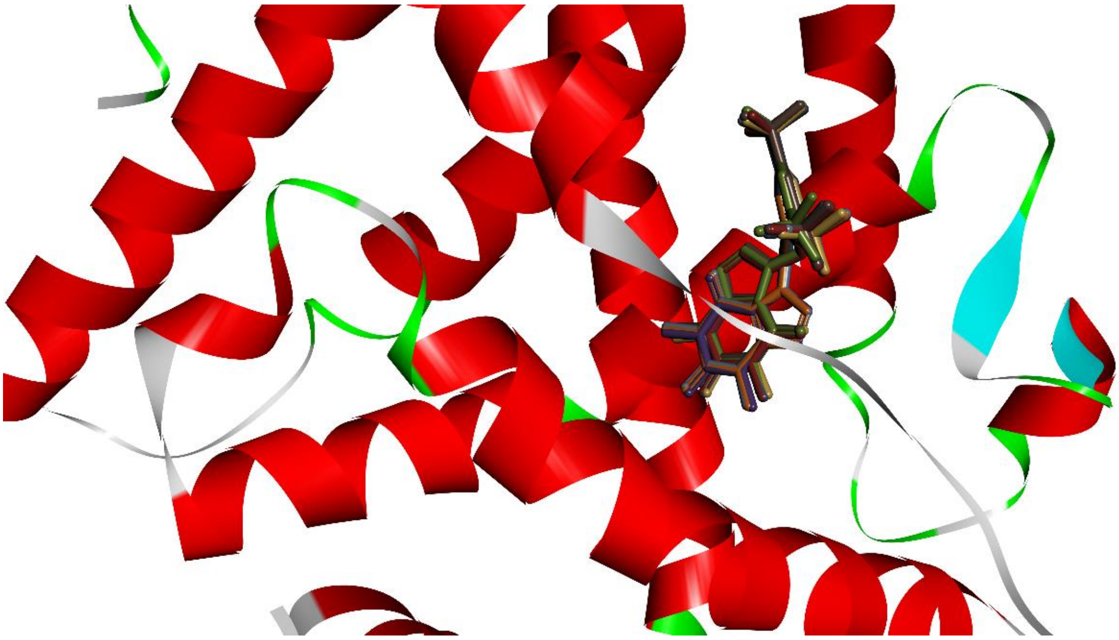

Figure S45. The overlaps of the molecular docking poses of **1d** against estrogen receptor.

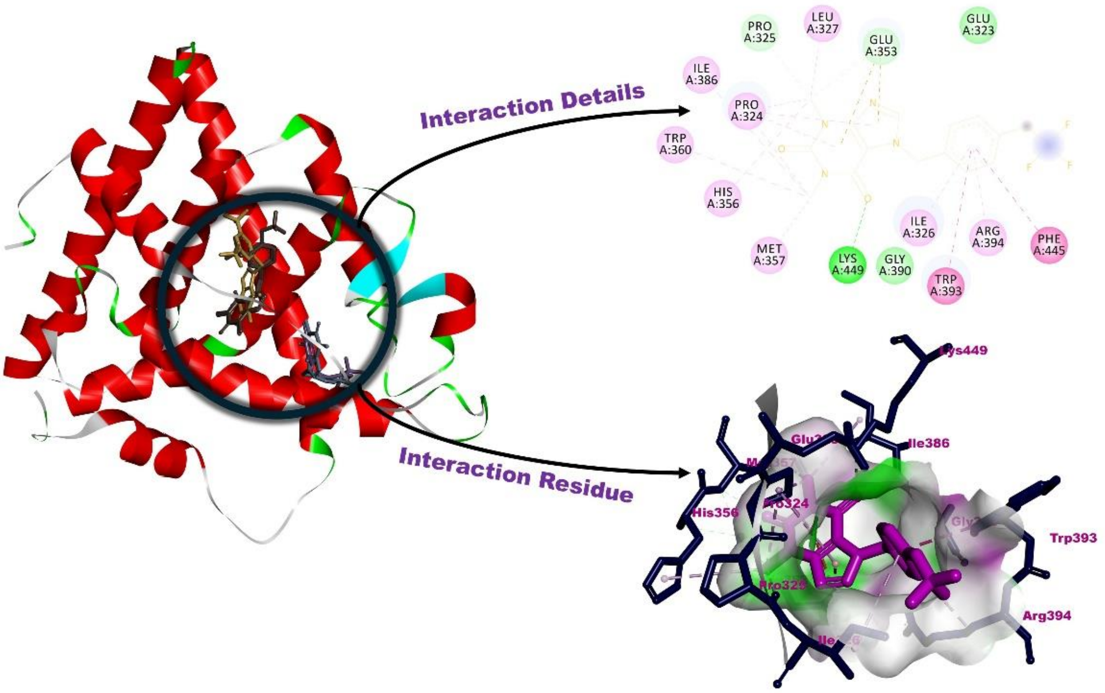

Figure S46. Interaction Residue and Details of **1e** against estrogen receptor.

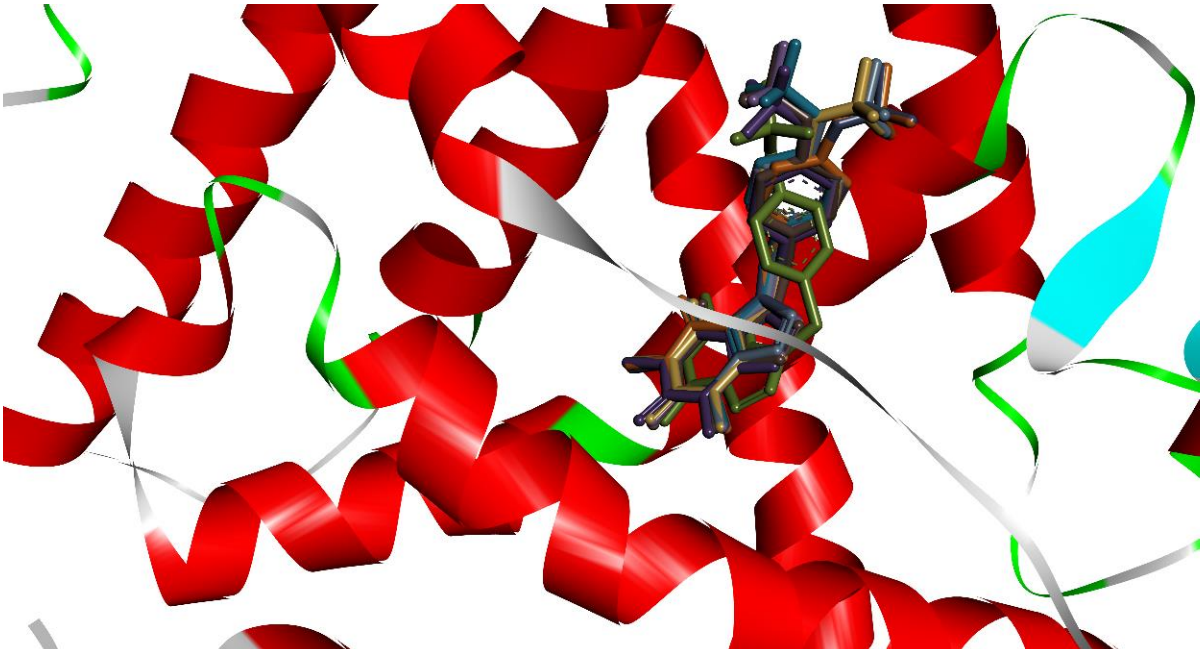

Figure S47. The overlaps of the molecular docking poses of **1e** against estrogen receptor.

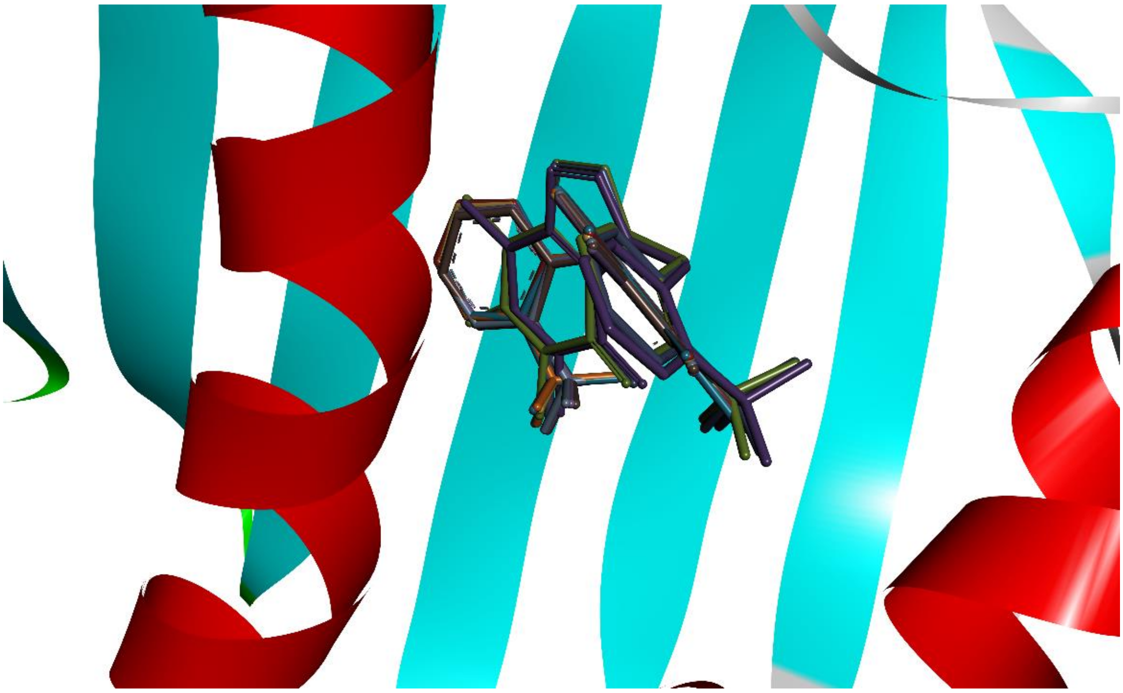

Figure S48. The overlaps of the molecular docking poses of **1a** against DNA Gyrase.

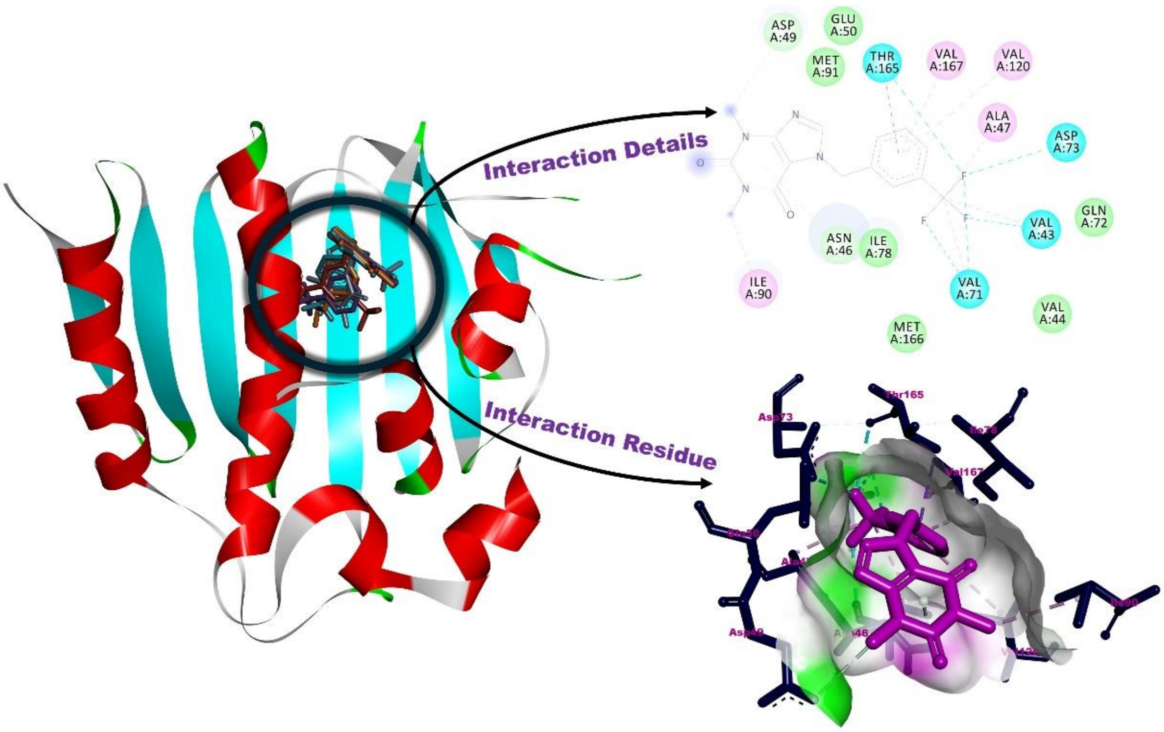

Figure S49. Interaction Residue and Details of **1b** against DNA Gyrase.

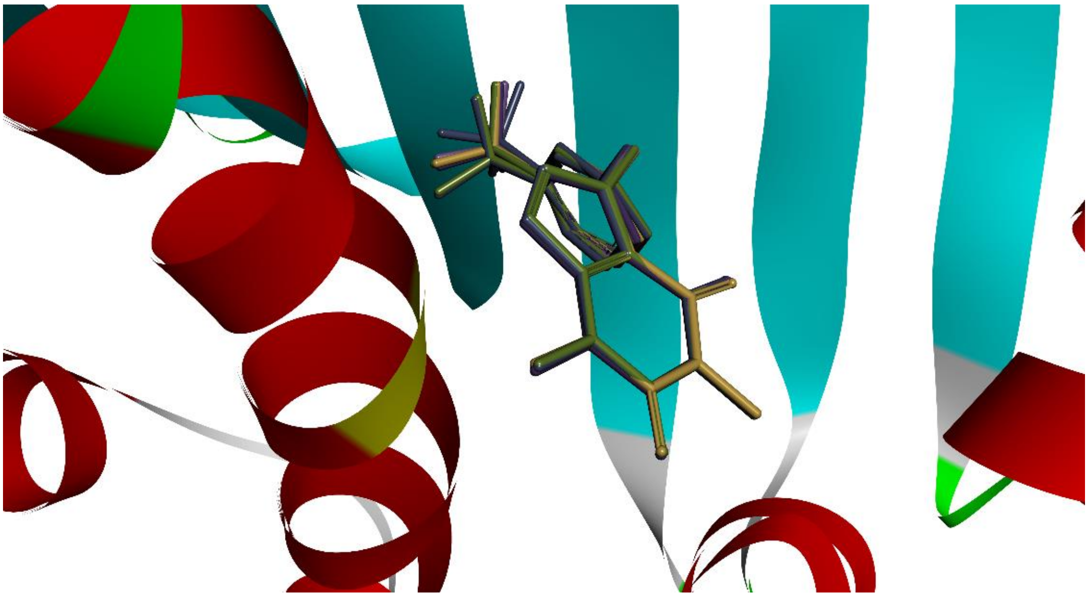

Figure S50. The overlaps of the molecular docking poses of **1b** against DNA Gyrase.

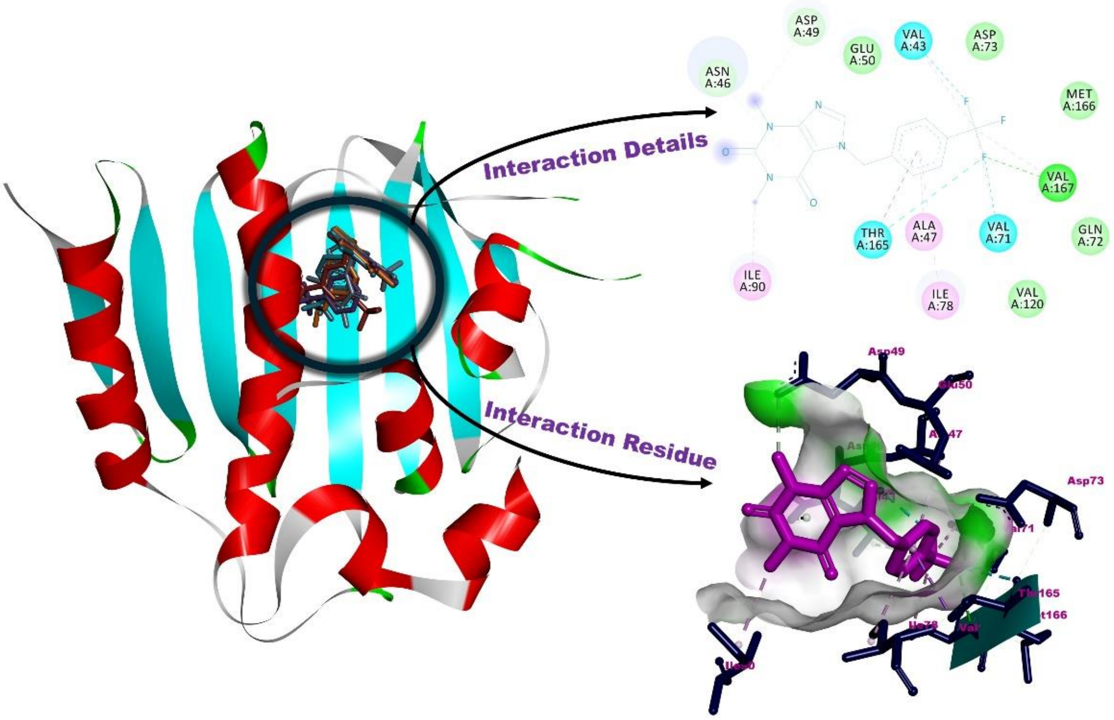

Figure S51. Interaction Residue and Details of **1c** against DNA Gyrase.

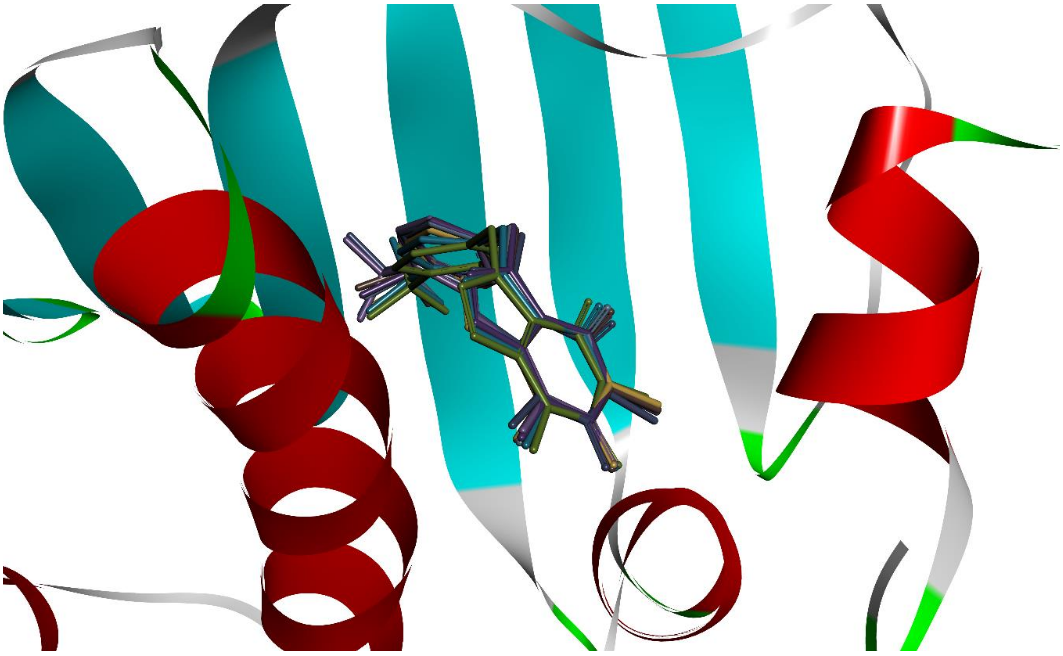

Figure S52. The overlaps of the molecular docking poses of **1c** against DNA Gyrase.

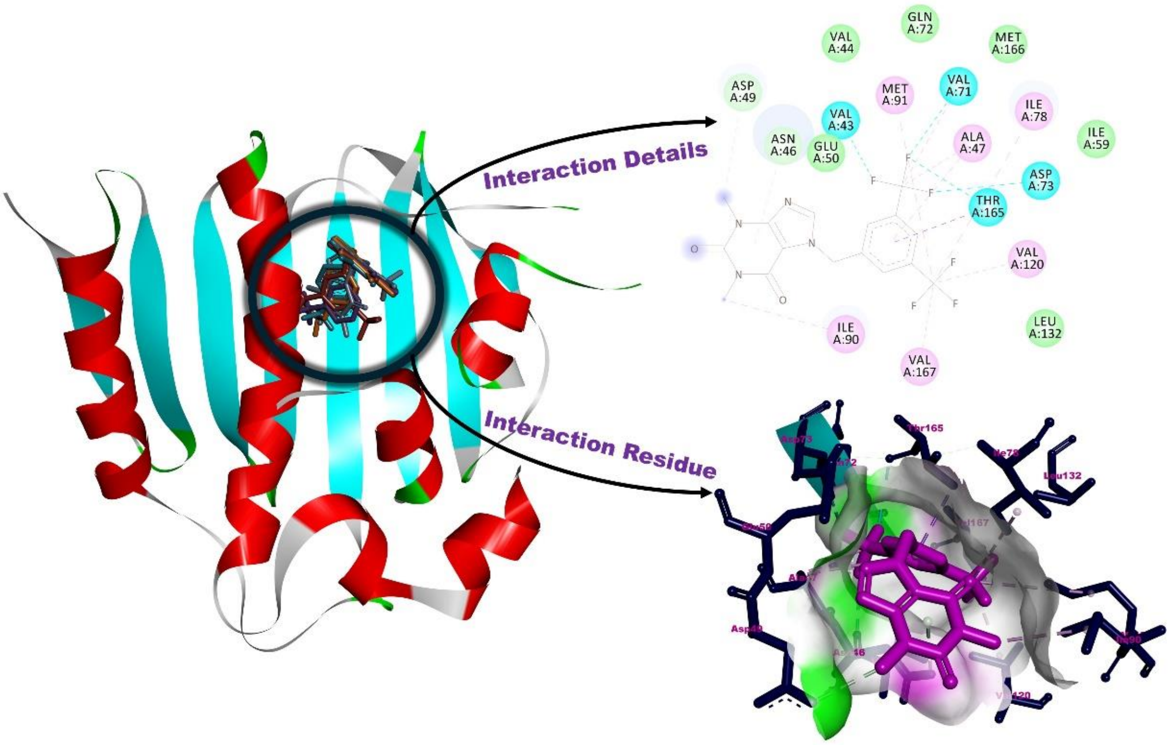

Figure S53. Interaction Residue and Details of **1d** against DNA Gyrase.

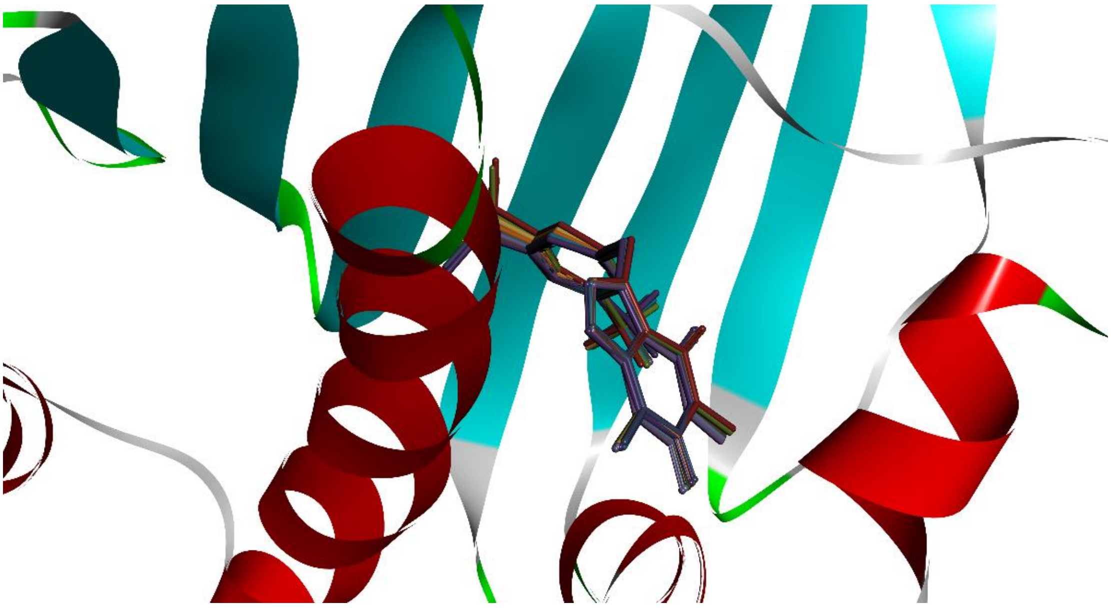

Figure S54. The overlaps of the molecular docking poses of **1d** against DNA Gyrase.

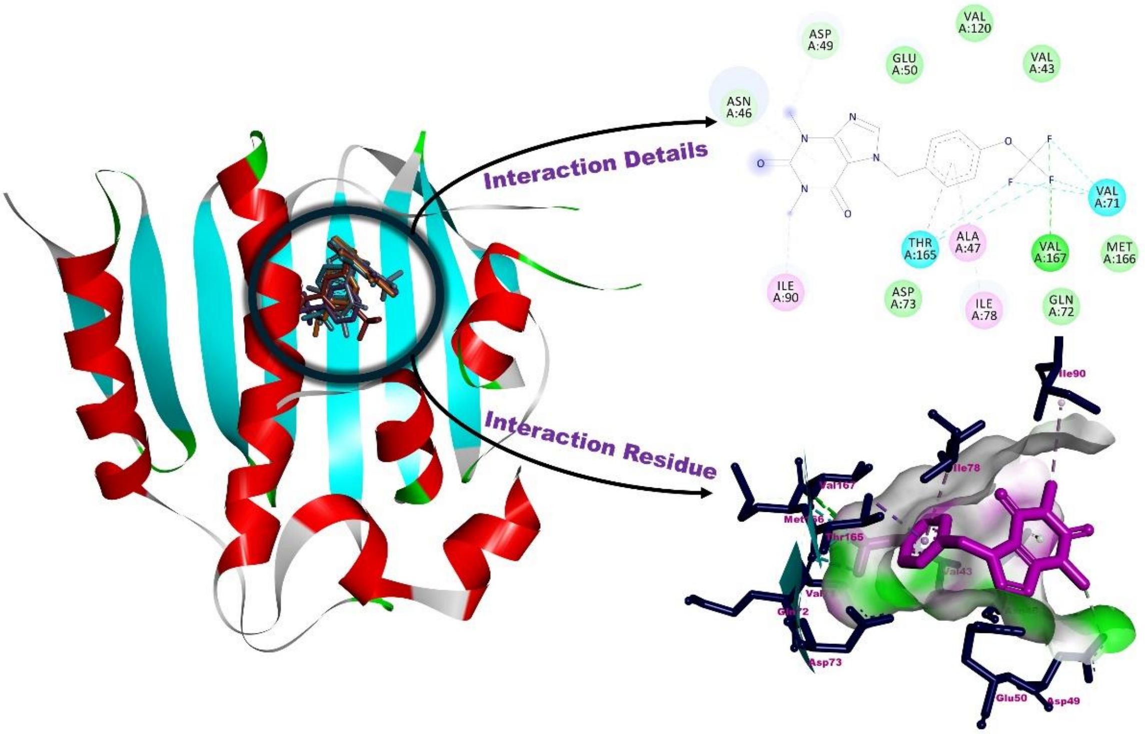

Figure S55. Interaction Residue and Details of **1e** against DNA Gyrase.

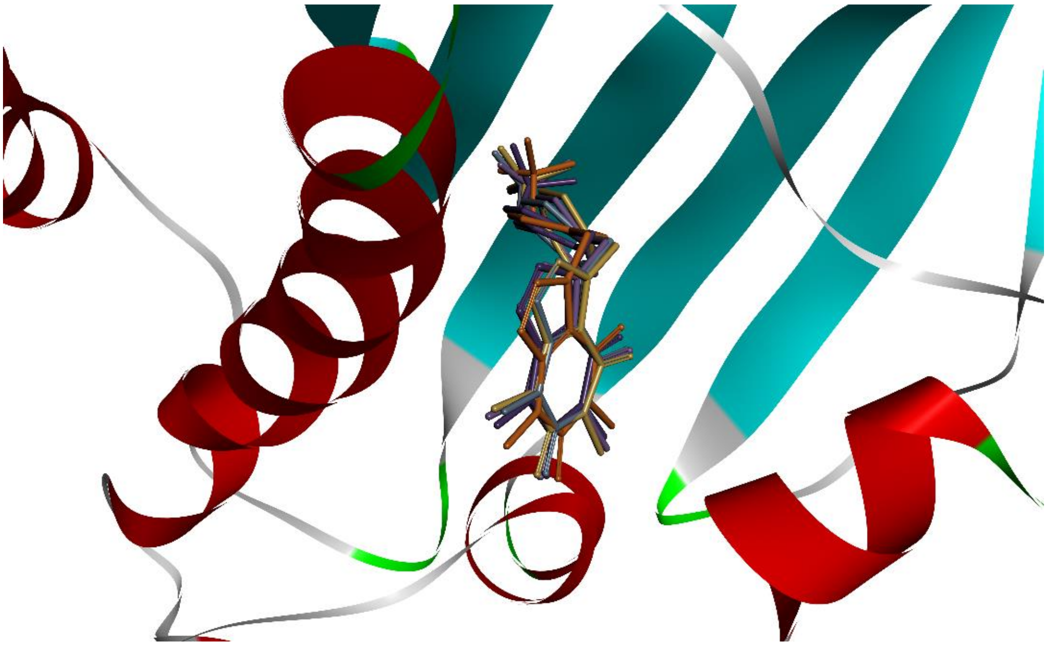

Figure S56. The overlaps of the molecular docking poses of **1e** against DNA Gyrase.

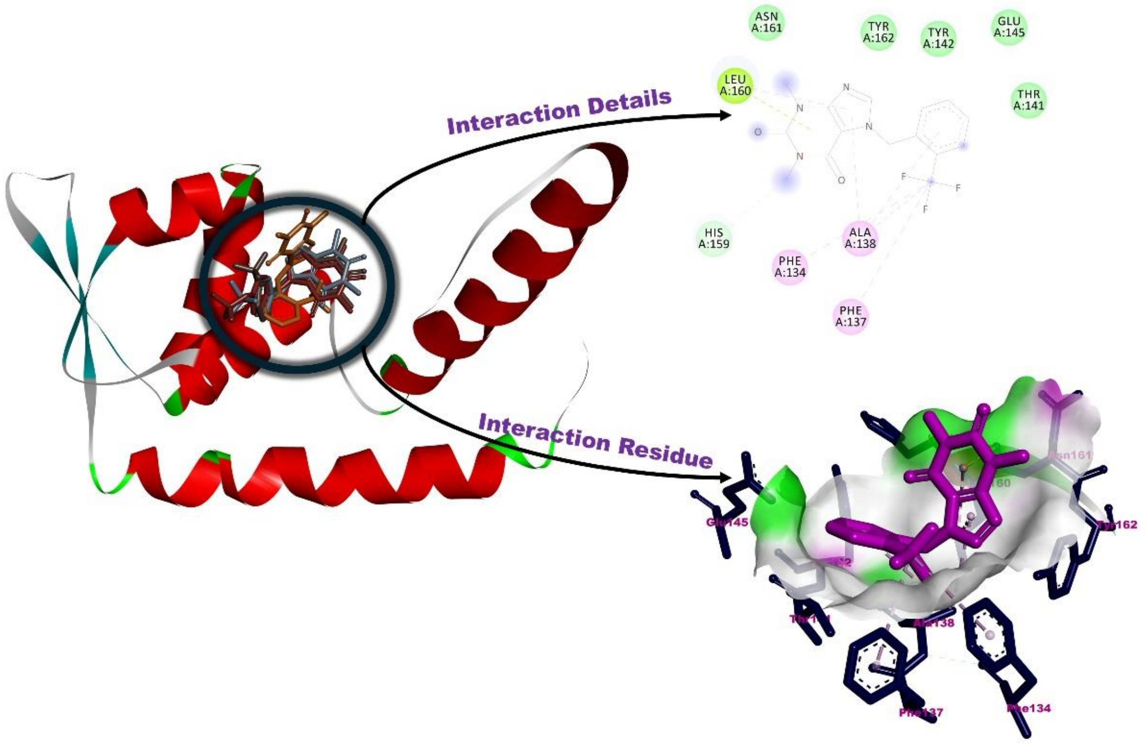

Figure S57. Interaction Residue and Details of **1a** against SarA.

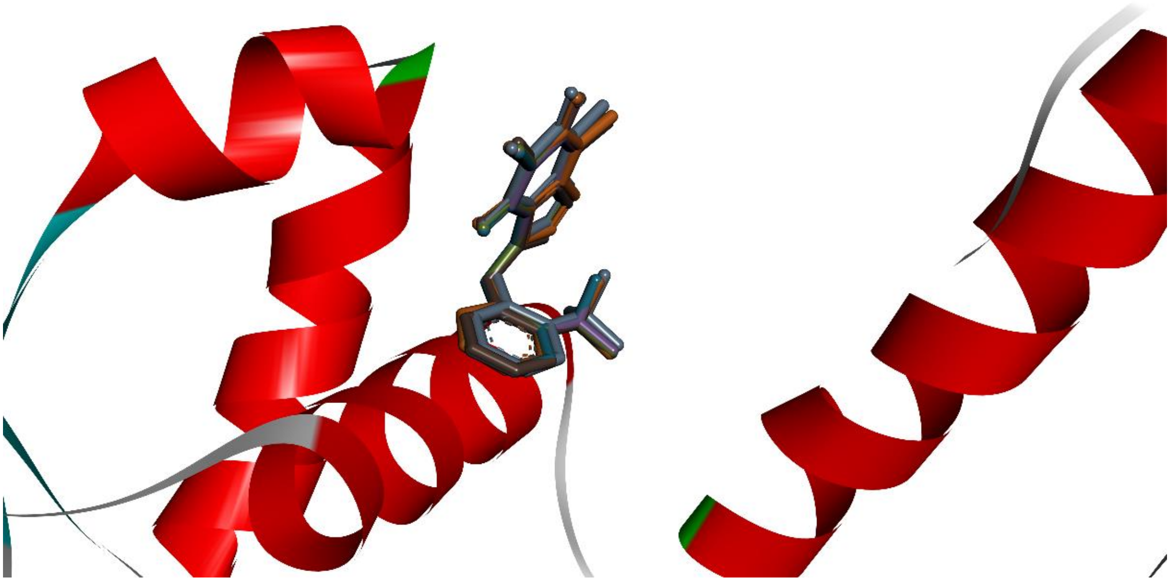

Figure S58. The overlaps of the molecular docking poses of **1a** against SarA.

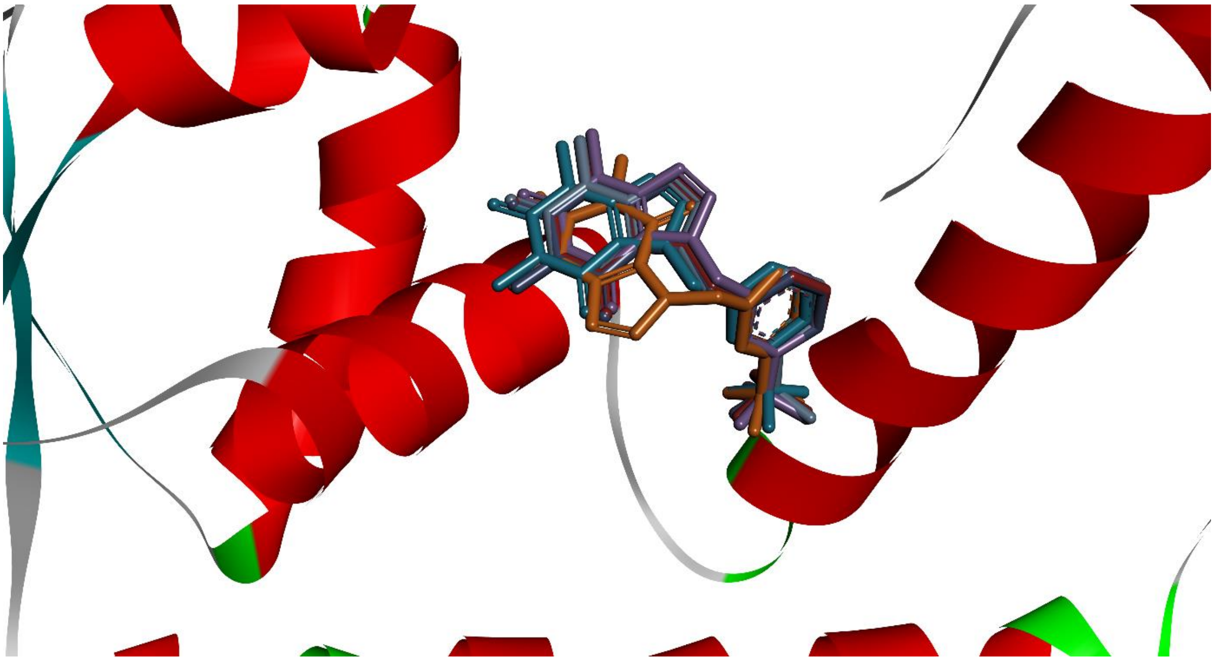

Figure S59. The overlaps of the molecular docking poses of **1b** against SarA.

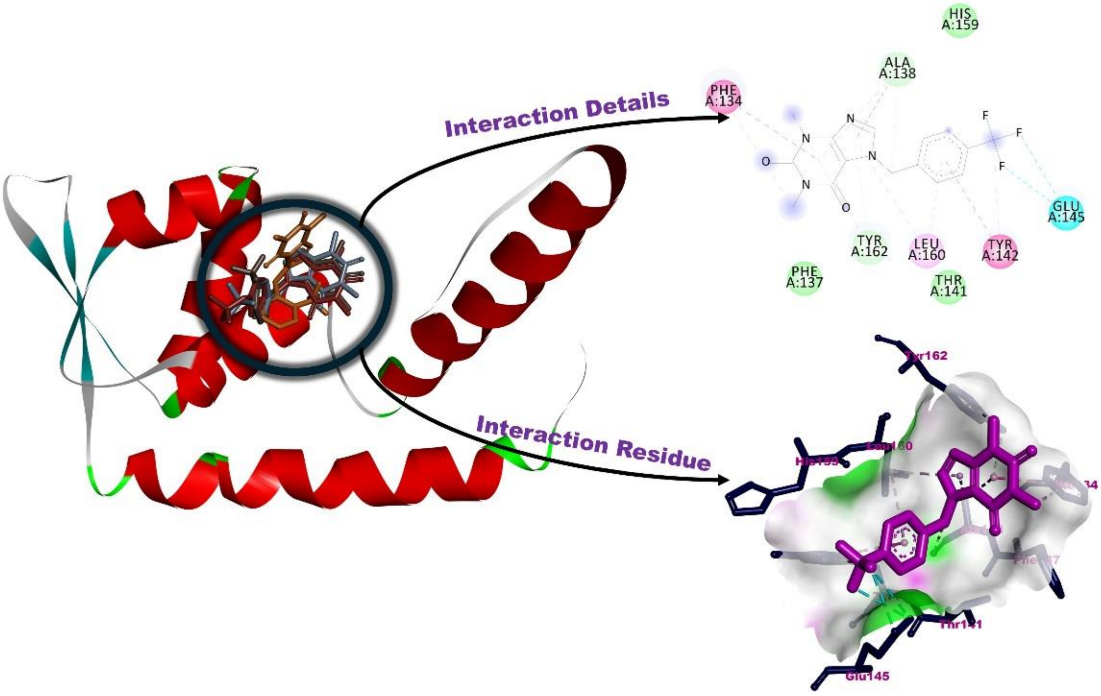

Figure S60. Interaction Residue and Details of **1c** against SarA.

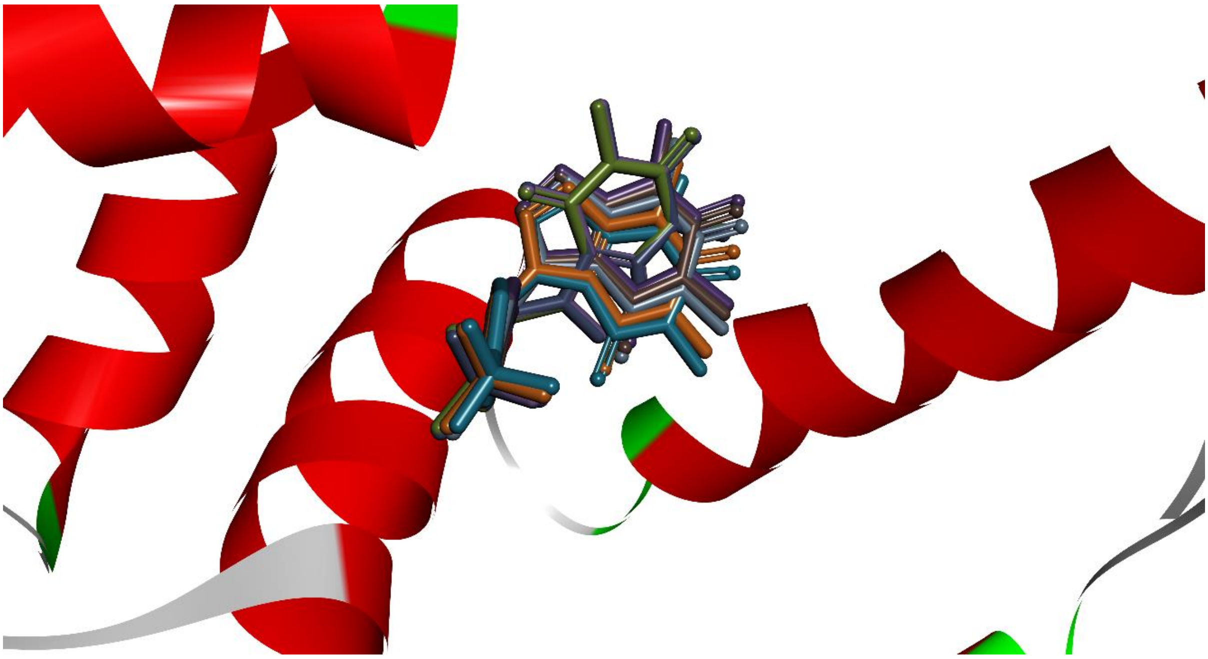

Figure S61. The overlaps of the molecular docking poses of **1c** against SarA.

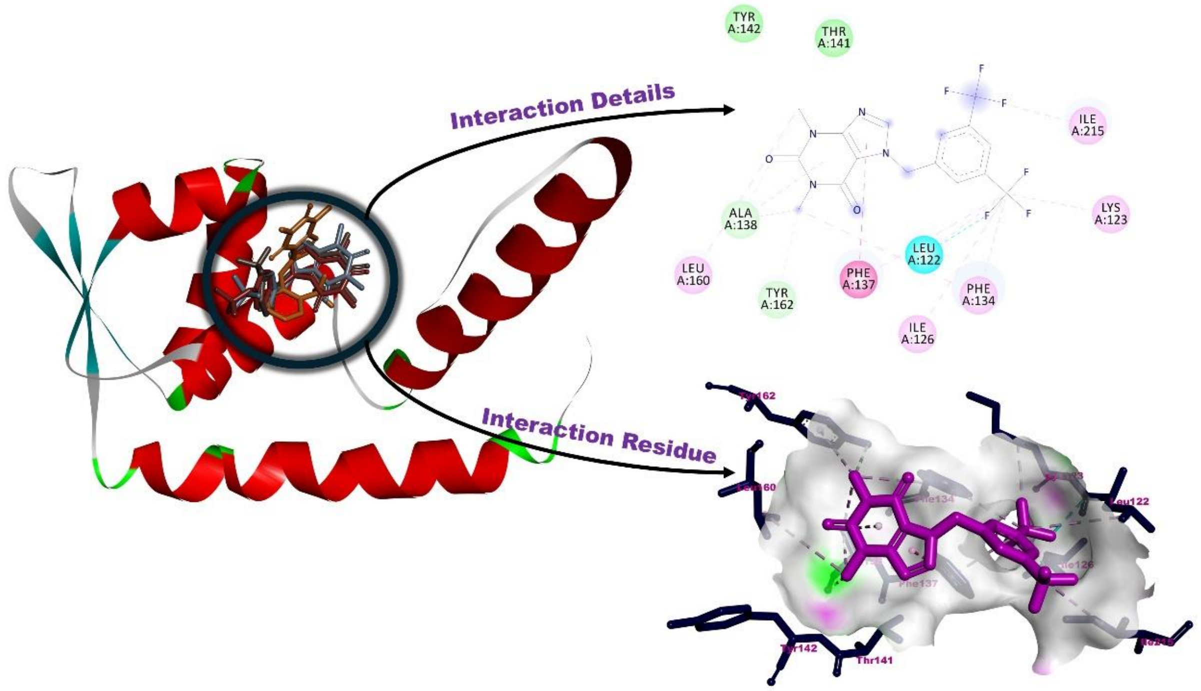

Figure S62. Interaction Residue and Details of **1d** against SarA.

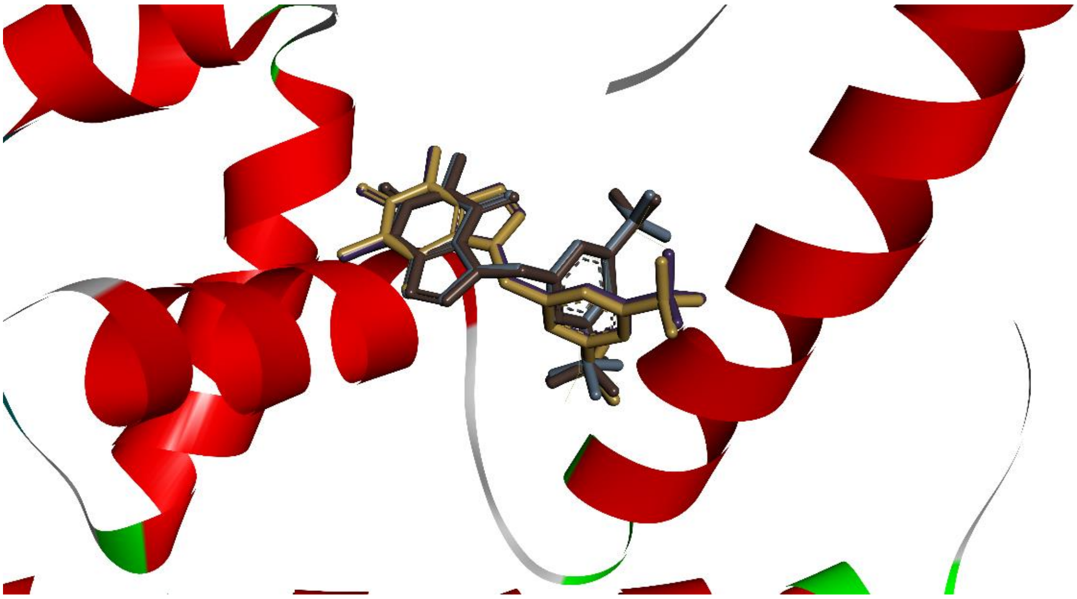

Figure S63. The overlaps of the molecular docking poses of **1d** against SarA.

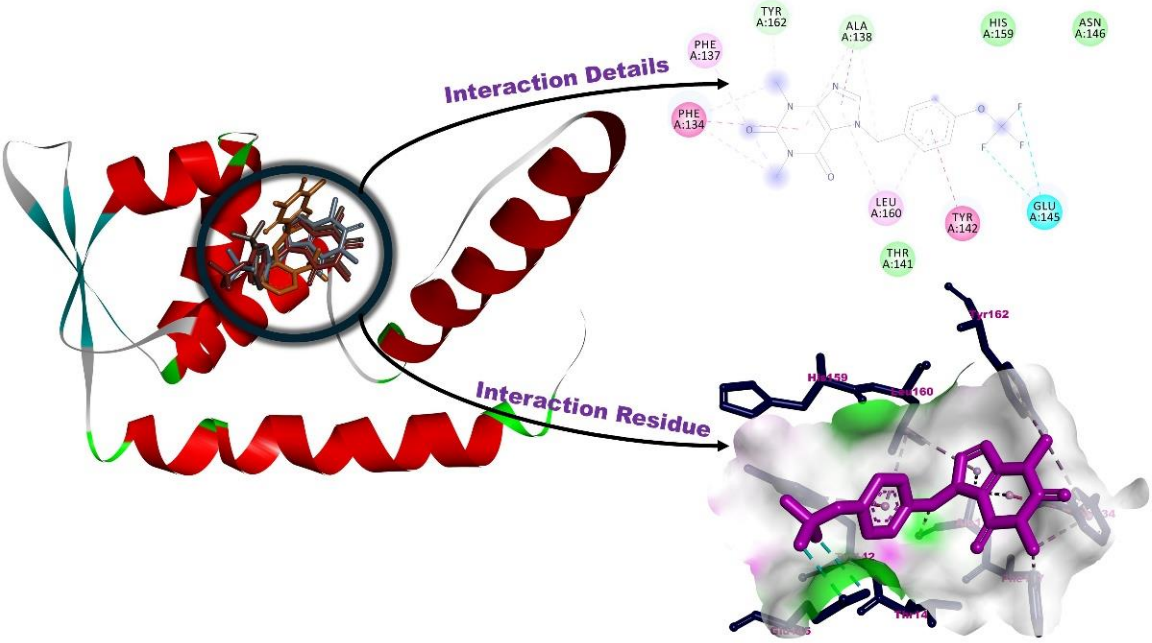

Figure S64. Interaction Residue and Details of **1e** against SarA.

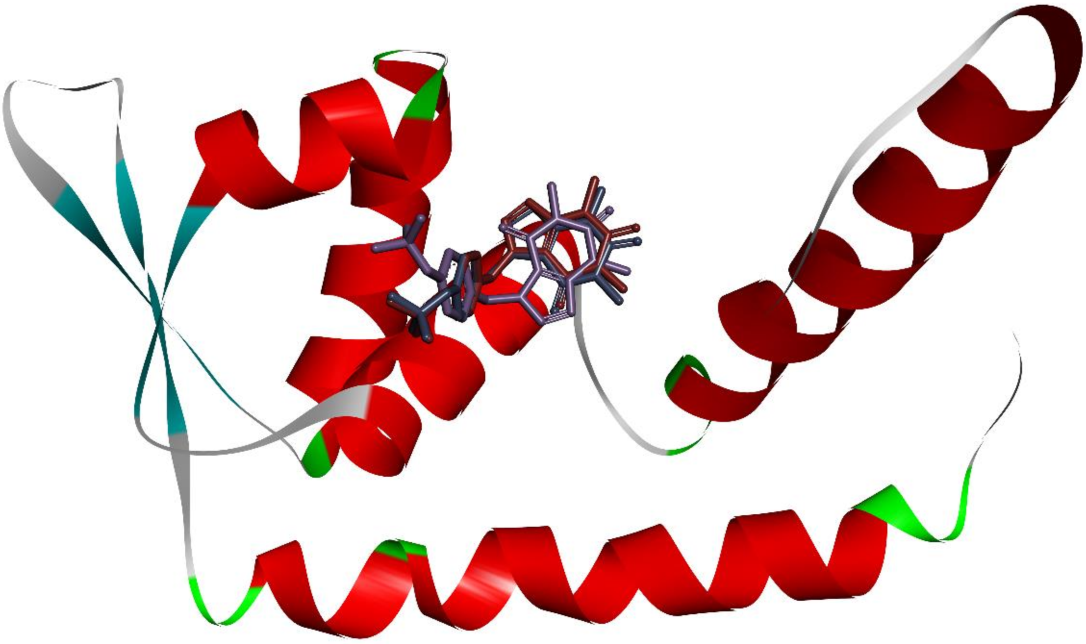

Figure S65. The overlaps of the molecular docking poses of **1e** against SarA .

Gene Symbol	Forward Primer (5' → 3')	Reverse Primer (5' → 3')
CASP3	GGAAGCGAATCAATGGACTCTGG	GCATCGACATCTGTACCAGACC
CASP8	AGAAGAGGGTCATCCTGGGAGA	TCAGGACTTCCTTCAAGGCTGC
CASP9	GTTTGAGGACCTTCGACCAGCT	CAACGTACCAGGAGCCACTCTT
BAX	TCAGGATGCGTCCACCAAGAAG	TGTGTCCACGGCGGCAATCATC
BAK1	TTACCGCCATCAGCAGGAACAG	GGAACTCTGAGTCATAGCGTCG
PUMA	TCCTCAGCCCTCCCTGTCAC	CCATTTCTGGGGCTCCAGGA
NOXA	CTGGAAGTCGAGTGTGCTACTC	TGAAGGAGTCCCCTCATGCAAG
CYCS	AAGGGAGGCAAGCACAAGACTG	CTCCATCAGTGTATCCTCTCCC
BCL2	ATCGCCCTGTGGATGACTGAGT	GCCAGGAGAAATCAAACAGAGGC
BCL-XL	GCCACTTACCTGAATGACCACC	AACCAGCGGTTGAAGCGTTCCT
MCL1	CCAAGAAAGCTGCATCGAACCAT	CAGCACATTCCTGATGCCACCT
TP53	CCTCAGCATCTTATCCGAGTGG	TGGATGGTGGTACAGTCAGAGC
CDKN1A (p21)	AGGTGGACCTGGAGACTCTCAG	TCCTCTTGGAGAAGATCAGCCG
FAS	GGACCCAGAATACCAAGTGCAG	GTTGCTGGTGAGTGTGCATTCC
GADD45A	CTGGAGGAAGTGCTCAGCAAAG	AGAGCCACATCTCTGTCGTCGT
ATF4	AACCTCATGGGTTCTCCAGCGA	CTCCAACATCCAATCTGTCCCG
GAPDH (Ref)	GTCTCCTCTGACTTCAACAGCG	ACCACCCTGTTGCTGTAGCCAA

Figure S66. Primer Sequences Used in Quantitative RT-PCR Analysis
